# NADPH-generating systems in bacteria and archaea

**DOI:** 10.3389/fmicb.2015.00742

**Published:** 2015-07-29

**Authors:** Sebastiaan K. Spaans, Ruud A. Weusthuis, John van der Oost, Servé W. M. Kengen

**Affiliations:** ^1^Laboratory of Microbiology, Wageningen UniversityWageningen, Netherlands; ^2^Bioprocess Engineering, Wageningen UniversityWageningen, Netherlands

**Keywords:** NADPH regeneration, pentose phosphate pathway, isocitrate dehydrogenase, malic enzyme, transhydrogenase, GAPN, ferredoxin:NADP^+^ oxidoreductase, hydrogenase

## Abstract

Reduced nicotinamide adenine dinucleotide phosphate (NADPH) is an essential electron donor in all organisms. It provides the reducing power that drives numerous anabolic reactions, including those responsible for the biosynthesis of all major cell components and many products in biotechnology. The efficient synthesis of many of these products, however, is limited by the rate of NADPH regeneration. Hence, a thorough understanding of the reactions involved in the generation of NADPH is required to increase its turnover through rational strain improvement. Traditionally, the main engineering targets for increasing NADPH availability have included the dehydrogenase reactions of the oxidative pentose phosphate pathway and the isocitrate dehydrogenase step of the tricarboxylic acid (TCA) cycle. However, the importance of alternative NADPH-generating reactions has recently become evident. In the current review, the major canonical and non-canonical reactions involved in the production and regeneration of NADPH in prokaryotes are described, and their key enzymes are discussed. In addition, an overview of how different enzymes have been applied to increase NADPH availability and thereby enhance productivity is provided.

## Introduction

Reduced nicotinamide adenine dinucleotide phosphate (NADPH) is an essential electron donor in all eukaryotes, bacteria, and archaea. Not only is NADPH vital for the generation of reactive oxygen species (ROS) (Foreman et al., 2003; Brown and Griendling, 2009; Bylund et al., 2010; Nakamura et al., 2012) and the anti-oxidative defense mechanisms of most organisms (Nordberg and Arnér, 2001; Minard and Mcalister-Henn, 2005; Singh et al., 2008), most importantly, it is also the driving force of most biosynthetic enzymatic reactions, including those responsible for the biosynthesis of all major cell components, such as DNA and lipids (Arnér and Holmgren, 2000; Nordberg and Arnér, 2001; Hügler et al., 2002; Koh et al., 2004; Minard and Mcalister-Henn, 2005; Singh et al., 2008; Miller and Auchus, 2011). Owing to this essential role in biosynthesis, NADPH availability has been of major interest to industry (Papagianni, 2012; Lee et al., 2013b). Many natural products of industrial importance are complex secondary metabolites, the production of which often involves NADPH-dependent enzymes. To synthesize such products using purified enzyme systems *in vitro* would require the addition of huge amounts of NADPH in order to sustain production. From an industrial point of view, this would be too expensive. Hence, *in situ* NADPH regeneration from its oxidized counterpart (NADP^+^) is required (Chenault et al., 1988; Wandrey, 2004; Wichmann and Vasic-Racki, 2005; Zhang and Xu, 2010; Uppada et al., 2014).

Regeneration of NADPH *in situ* can be achieved by various strategies, including chemical, electrochemical, photochemical, and enzyme-based methods (Chenault and Whitesides, 1987; Zhao and Van Der Donk, 2003; Wichmann and Vasic-Racki, 2005; Liu and Wang, 2007; Zhang and Xu, 2010; Uppada et al., 2014). NADPH can be regenerated enzymatically by complementing the *in vitro* system with additional enzymatic reactions or by using substrate-coupled reaction systems. The latter system employs enzymes that use both NADP^+^ and NADPH and that are able to catalyze the synthesis of the desired product from one substrate and cofactor regeneration with another substrate (Chenault et al., 1988; Van Der Donk and Zhao, 2003; Liu and Wang, 2007). However, reduced productivity compared to systems without *in situ* regeneration and problems associated with enzyme stability make these options unattractive.

Microbial *in vivo* production systems also provide *in situ* NADPH regeneration and have several advantages when compared to *in vitro* systems. For example, microbes are able to grow on inexpensive renewable feedstocks that provide the organisms with reductant for the regeneration of NADPH. They also contain numerous pathways, involving stable and highly specific enzymes, thus obviating the need for expensive enzyme purification. In addition, our knowledge of natural metabolic pathways is rapidly advancing, allowing for rational design toward product formation (Chemler et al., 2010; Siedler et al., 2011; Papagianni, 2012; Lee et al., 2013b). Therefore, it is not surprising that microbial conversion is the preferred method for the synthesis of a range of products.

With the possibility of engineering microbial metabolism to facilitate product formation, it became clear that NADPH availability remains a major hurdle in the efficient generation of many products. These products range from medicinal compounds (Chemler et al., 2010; Siedler et al., 2011; Zhao et al., 2011) and (essential) amino acids (Becker et al., 2007; Papagianni, 2012) to molecules used as biofuels (Asadollahi et al., 2009; Kim et al., 2011; Peralta-Yahya et al., 2012) and building blocks for biodegradable plastic (Kabir and Shimizu, 2003). Given its involvement in a multitude of crucial biological functions and its importance in biosynthesis, NADPH is without question an essential molecule. Hence, a key question arises: what are the major NADPH-generating reactions and systems?

Traditionally, the dehydrogenase reactions of the oxidative pentose phosphate pathway (oxPPP), the Entner–Doudoroff (ED) pathway, and the isocitrate dehydrogenase step of the tricarboxylic acid (TCA) cycle have been considered the major sources of NADPH. However, the importance of other NADPH-generating enzymes, such as transhydrogenases, glucose dehydrogenases, and non-phosphorylating glyceraldehyde 3-phosphate dehydrogenase (GAPN), is becoming clear, indicating that the traditional view is over-simplistic (Sauer et al., 2004; Matsubara et al., 2011; Brasen et al., 2014).

In this review, we describe the major canonical and non-canonical biochemical mechanisms that are involved in the production and regeneration of NADPH in prokaryotes and discuss the key enzymes involved. We have divided the mechanisms into those that are directly coupled to central carbon metabolism and those that are not (Table 1). In addition, we briefly address how different enzymes have been applied to increase NADPH availability and thereby enhance NADPH-dependent biotransformation processes.

**Table 1:** **Overview of major canonical and non-canonical NADPH-generating enzymes**.

	**Enzyme**	**EC:number**	**Pathway**	Bacteria^*^ (%)	Archaea^*^ (%)	**Additional cofactors**	Applied^**^	Δ_*r*_G^'m^^***^ (kJ/mol)
NADPH-generating enzymes coupled to central carbon metabolism	G6PDH	EC:1.1.1.49	oxPPP, ED	66	0	n.a.	yes	−2.3±2.6
	6PGDH	EC:1.1.1.44	oxPPP	62	27	n.a.	yes	−6.0±6.3
	IDH	EC:1.1.1.42	TCA cycle	82	59	n.a.	yes	−10.7±6.3
	ME	EC:1.1.1.40	Anaplerotic node	47	25	n.a.	no	−3.1±6.2
	GAPN	EC:1.2.1.9	EMP	12	31	n.a.	yes	−36.1±1.1
	NADP^+^-GAPDH	EC:1.2.1.13	EMP	n.d.	n.d.	n.a.	yes	25.9±1.0
	GDHs	EC:1.1.1.47,EC:1.1.1.119	Modified EDs	18	10	n.a.	yes	−2.4±2.2
				n.d.	n.d.	n.a.		
NADPH-generating enzymes not coupled to central carbon metabolism	STH	EC:1.6.1.1	n.a.	19	0	NADH, FAD	yes	1.0±0.7
	H^+^-TH	EC:1.6.1.2	n.a.	50	5	NADH	yes	1.0±0.7
	FNR	EC:1.18.1.2	n.a.	63	29	FAD or FMN, Fd_red_	no	−15.6±11.7
	SH	EC:1.12.1.3	n.a.	10	6	FAD or FMN	no	−16.5±5.9
	NADK	EC:2.7.1.23	n.a.	96	98	NTP or poly(P), NAD^+^ or NADH	yes	n.a.

* *Distribution of the enzyme in completely sequenced prokaryotic genomes. All available prokaryotic genomes were downloaded to Excel from the IMG database on 6/16/2015. The analysis included only finished genomes (198 archaeal and 3380 bacterial genomes) and only one representative genome from each genus (83 archaeal and 636 bacterial genomes), to correct for the many draft genomes and for the many sequences in closely related genomes, respectively. The obtained distribution is merely an indication of the true distribution of the respective enzymes among prokaryotes because the database is incomplete and gene functions are often automatically annotated*.

** *Use of the enzyme in metabolic engineering to increase NADPH availability and enhance biotransformation processes*.

*** *Gibbs free energies (Δ_r_G^′m^) calculated using the biochemical thermodynamics calculator eQuilibrator 2.0 (Flamholz et al., 2012; Noor et al., 2012, 2013), which calculates Δ_r_G^′m^. This term describes the change in Gibbs free energy due to a chemical reaction at a particular pH and ionic strength (subscript r), using 1 mM concentrations for all reactants (superscript m). Default settings (pH 7 and ionic strength 0.1 M) were used for all reactions*.

*n.a., not applicable; n.d., no data available in the IMG database; G6PDH, glucose-6-phosphate dehydrogenase; 6PGDH, 6-phosphogluconate dehydrogenase; IDH, isocitrate dehydrogenase; ME, malic enzyme; GAPN, non-phosphorylating glyceraldehyde 3-phosphate dehydrogenase; NADP^+^-GAPDH, NADP^+^-dependent phosphorylating glyceraldehyde 3-phosphate dehydrogenase; GDH, glucose dehydrogenase; STH, energy-independent soluble transhydrogenase; H^+^-TH, energy-dependent or proton-translocating, membrane-bound transhydrogenase; FNR, ferredoxin:NADP^+^ oxidoreductase; SH, cytosolic NADP^+^-reducing hydrogenase; NADK, NAD^+^ kinase*.

## Biosynthesis of NADP^+^

To maintain a sufficient quantity of NADP^+^ for the generation of NADPH, NADP^+^ biosynthesis is essential. While the necessity for NADP^+^ synthesis during cell proliferation is clear, the requirement for NADP^+^ synthesis in non-dividing cells might not be obvious. NADP(H) is generally thought of solely as a redox carrier that facilitates the transfer of electrons between two redox couples, a role that does not account for the need for constant resynthesis. The constant need to resynthesize NADP^+^ arises from its participation in other crucial biological processes (Agledal et al., 2010). In eukaryotes, for example, NADP^+^ serves as the substrate in the synthesis of nicotinic acid-adenine dinucleotide phosphate (NAADP), an important intracellular Ca^2+^-mobilizing messenger (Lee, 1997; Chini and De Toledo, 2002; Churamani et al., 2004; Patel et al., 2011). Although NAADP has also been found in bacteria (Churamani et al., 2004), its physiological relevance in these organisms remains to be established.

The enzymes responsible for NADP^+^ resynthesis, best studied in eukaryotes, mainly include NAD(P)^+^ nucleosidase (Vu et al., 1996; Ying, 2008), NADP^+^ phosphatase, and NADPH phosphatase. The enzymes in prokaryotes are less well-studied, but similar activities and proteins have been found in this domain (Mather and Knight, 1972; Everse et al., 1975; Davis, 1980; Penyige et al., 1996; Pollak et al., 2007; Kawai and Murata, 2008; Ghosh et al., 2010). NADP^+^ phosphatase and NADPH phosphatase, together with NAD(H) kinase, regulate the intracellular balance of NAD(H) and NADP(H) (Kawai and Murata, 2008). Like NADPH, NADH mainly serves to transfer electrons from one molecule to another. However, unlike NADPH, NADH is primarily involved in catabolic reactions (Ying, 2008). In addition to its redox function, NAD^+^ serves as a substrate for mono- and poly-ADP ribosylation, participates in histone deacetylation, and contributes to the production of the signaling molecule cyclic ADP-ribose. Most of these reactions have been characterized in eukaryotes, but the ribosylation reactions also play a role in toxin production by pathogenic bacteria (Ziegler, 2000; Pollak et al., 2007). The consumption of NADP^+^ is thus connected to the consumption of NAD^+^ and to the regulation of various major biological activities such as DNA repair, gene expression, apoptosis, nitrogen fixation, and calcium homeostasis (Ziegler, 2000; Pollak et al., 2007; Kawai and Murata, 2008; Ying, 2008; Agledal et al., 2010).

**Figure 1:**
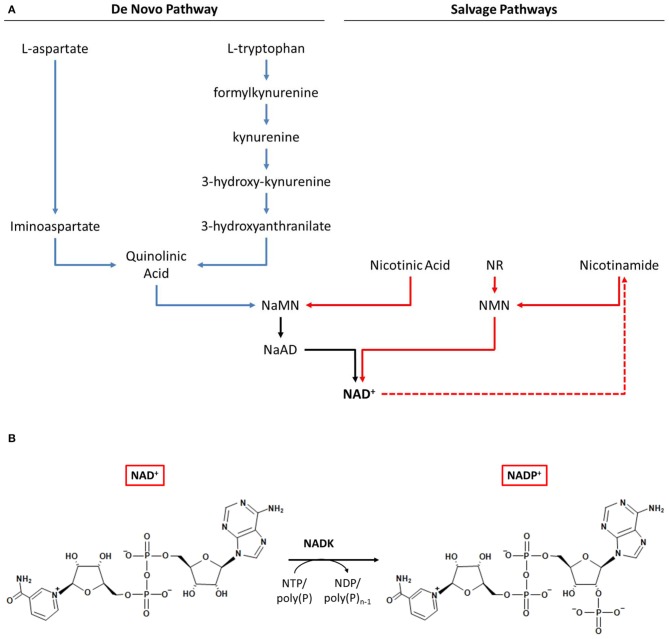
**NAD^+^ and NADP^+^ biosynthetic pathways in prokaryotes. (A)** *De novo* and salvage pathways for NAD^+^ biosynthesis. **(B)** NADP^+^ biosynthesis by NAD kinase. Abbreviations: NaMN, nicotinic acid mononucleotide; NaAD, nicotinic acid adenine dinucleotide; NR, nicotinamide riboside; NMN, nicotinamide mononucleotide; NAD, nicotinamide adenine dinucleotide; NADP, nicotinamide adenine dinucleotide phosphate; NADK, NAD kinase.

Given that the intracellular balance between NAD(H) and NADP(H) is regulated via the addition or removal of a phosphate group by NAD(H) kinase or NADP(H) phosphatase, respectively, it is clear that the biosynthesis of NAD^+^ plays a crucial role in the metabolism of NADP^+^. Two principal NAD^+^ biosynthesis pathways have been characterized: (1) the *de novo* pathway and (2) the salvage pathway (Magni et al., 1999, 2004; Begley et al., 2001). Both pathways have been reviewed recently (Pollak et al., 2007; Ying, 2008; Gazzaniga et al., 2009; Gossmann et al., 2012). In the *de novo* pathway, NAD^+^ is generated from quinolinic acid, which in prokaryotes is produced from either L-aspartate or L-tryptophan (Magni et al., 1999; Sakuraba et al., 2002; Kurnasov et al., 2003). In the salvage pathway, degradation products containing a pyridine ring, namely nicotinic acid and nicotinamide, are utilized to regenerate NAD^+^ (Figure 1A) (Magni et al., 1999; Gazzaniga et al., 2009).

A portion of the synthesized NAD^+^ can be converted into NADP^+^ by NAD kinase (NADK) (Figure 1B). In the NAD^+^ synthesis pathways, several variations exist and multiple enzymes are involved (Bi et al., 2011; Gossmann et al., 2012). In contrast, NADK is the sole enzyme able to generate NADP^+^ *de novo*. NAD kinase is found in archaea (Sakuraba et al., 2005), bacteria (Kawai et al., 2000, 2001; Ochiai et al., 2004), and eukaryotes (Tseng et al., 1979; Butler and Mcguinness, 1982) and has been proven to be essential in prokaryotes (Kobayashi et al., 2003; Sassetti et al., 2003). According to the literature, only one species not able to synthesize NADP^+^ from NAD^+^ has been identified: *Chlamydia trachomatis* (Kawai and Murata, 2008; Szaszák et al., 2011). The intracellular parasite appears to lack NADK and hence relies completely on the metabolism of its host cell.

According to Kawai and Murata (2008), NADK orthologs can be classified into three types according to their substrate specificity: (I) NADKs that utilize both ATP and inorganic polyphosphate (poly(P)) as a phosphoryl donor and phosphorylate both NAD^+^ and NADH. This type of NADK has been identified in gram-positive bacteria and archaea. (II) NADKs that utilize ATP but not poly(P) and phosphorylate both NAD^+^ and NADH. This type has been identified in eukaryotes. (III) NADKs that utilize ATP but not poly(P) and phosphorylate NAD^+^ but not NADH. The latter type of NADK has been identified in gram-negative bacteria. Because of its vital role in NADP^+^ synthesis, NADK has received much attention since its discovery in 1950 (Kornberg, 1950). Hence, many papers about its structure, function, and application are available, including reviews by Kawai and Murata (2008), Shi et al. (2009), and Agledal et al. (2010).

## Redox potential of NADP^+^/NADPH

The redox potential of NADP^+^/NADPH at standard physiological conditions is identical to that of NAD^+^/NADH (${\text{E}}_{0}^{\text{′}}$: −320 mV). However, the function of the cofactors is different. NADP^+^/NADPH is used for anabolic redox reactions, whereas NAD^+^/NADH is used for oxidation reactions. This is possible because NADP^+^/NADPH is generally maintained in a reduced state and NAD^+^/NADH in an oxidized state (Harold, 1986). The ratios for the electron carriers have been reported, and the values can differ by several orders of magnitude, depending on the organism and growth conditions. For example, reported NAD^+^/NADH values range from 3.74 to 31.3 for bacterial cells (Andersen and von Meyenburg, 1977; Thauer et al., 1977; Bennett et al., 2009; Amador-Noguez et al., 2011) and reach 1820 for mammalian cells (Veech et al., 1969), whereas NADP^+^/NADPH values range from 0.017 to 0.95 (Andersen and von Meyenburg, 1977; Thauer et al., 1977; Bennett et al., 2009; Amador-Noguez et al., 2011). This indicates that the actual redox potential of both redox couples can deviate significantly from the standard potential (i.e., generally more negative/positive than −320 mV for (NADP^+^/NADPH)/(NAD^+^/NADH), respectively). Such deviation affects the Gibbs energy of the NADPH-generating reactions and the feasibility of converting NADH into NADPH. To assess the feasibility of various NADPH-generating reactions, we calculated the Gibbs energies (Δ_*r*_G^′m^) using the biochemical thermodynamics calculator eQuilibrator 2.0 (Flamholz et al., 2012; Noor et al., 2012, 2013), setting all reactants at a physiological concentration of 1 mM (Table 1). However, even under these conditions, several reactions appeared to be at equilibrium or even slightly endergonic. At NADP^+^/NADPH ratios lower than 1, the reaction becomes even less feasible, indicating that the actual concentration of the reaction components must be substantially different for NADPH generation to proceed.

## Systems for NADPH generation

NADP^+^ synthesized by NADK or generated by NADPH-oxidizing reactions is eventually reduced to NADPH. To ensure that the cellular redox balance is maintained in the absence of *de novo* NADP^+^ synthesis and NADP^+^ consumption, the catabolic fluxes through the NADPH-regenerating reactions must be matched to the anabolic demand. However, this is generally not the case (Fuhrer and Sauer, 2009). In addition, the precise NADPH formation rate depends on fluxes through the generating pathways, which in turn vary with different growth conditions (Dauner et al., 2001; Zhao et al., 2004a; Kanai et al., 2011). Therefore, prokaryotes must have other network-wide biochemical mechanisms that maintain the cellular redox balance (Fuhrer and Sauer, 2009). The exact mechanisms are not fully understood and are beyond the scope of this review, but papers about the topic are available (Singh et al., 2008; Fuhrer and Sauer, 2009).

Although numerous reactions reduce NADP^+^ to NADPH (at least 143 reactions according to the MetaCyc database, accessed on 03/30/15), only a few have been thought to contribute significantly. However, new studies have demonstrated the importance of other NADPH-generating reactions. The present review provides a general overview of the known major NADPH-generating reactions and discusses the key enzymes involved. We have divided the reactions into (I) those that are directly coupled to central carbon metabolism and (II) those that are not (Table 1). The enzymes that comprise the first group are the oxPPP enzymes glucose-6-phosphate dehydrogenase and 6-phosphogluconate dehydrogenase; isocitrate dehydrogenase of the TCA cycle; malic enzyme; and three enzymes involved in non-canonical NADPH-generating reactions: phosphorylating GAP dehydrogenase (NADP^+^-GAPDH), non-phosphorylating GAP dehydrogenase (GAPN), and glucose dehydrogenase. The enzymes in the second group are transhydrogenases (NADH:NADP^+^), ferredoxin:NADP^+^ oxidoreductase, hydrogenases (H_2_:NADP^+^), and NAD(H) kinase, although the latter is a *de novo* NADP^+^/NADPH-synthesizing enzyme rather than an NADPH-regenerating enzyme. NAD(H) kinase is included because it plays an essential role in NADP^+^ biosynthesis and the regulation of the NAD(H)/NADP(H) balance offers a potential strategy for improving the biosynthesis of industrially valuable metabolites.

## NADPH-generating reactions coupled to central carbon metabolism

### Glucose-6-phosphate dehydrogenase and 6-phosphogluconate dehydrogenase

**Figure 2:**
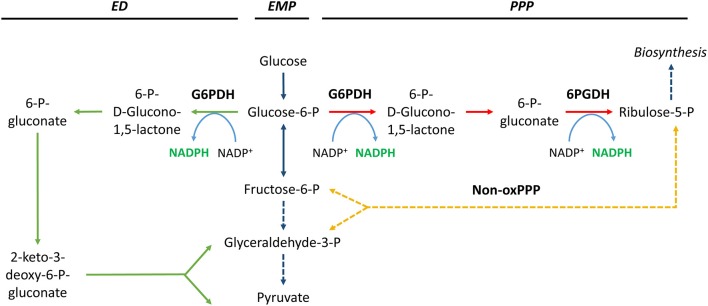
**NADPH generation by glucose-6-phosphate dehydrogenase (G6PDH) and 6-phosphogluconate dehydrogenase (6PGDH)**. Solid and dashed lines indicate single and lumped reactions, respectively. Pathway abbreviations: ED, Entner–Doudoroff pathway (in green); EMP, Embden–Meyerhof–Parnas pathway (in blue); PPP, pentose phosphate pathway (oxidative phase in red, non-oxidative phase in yellow).

The oxidative pentose phosphate pathway (oxPPP) enzymes glucose-6-phosphate dehydrogenase (G6PDH, EC:1.1.1.49) and 6-phosphogluconate dehydrogenase (6PGDH, EC:1.1.1.44) have a central role in metabolism in many microbes (Table 1). The enzymes are involved in the conversion of glucose-6-phosphate into ribulose-5-phosphate (Figure 2), a precursor of important molecules such as nucleic acids, and are generally considered a major source of NADPH. The importance of the oxPPP enzymes as a source of NADPH has been demonstrated in various organisms (Summers et al., 1995; Christiansen et al., 2002; Sauer et al., 2004). Flux through these enzymes reportedly increases when NADPH requirements are high (Obanye et al., 1996; Christiansen et al., 2002; Bartek et al., 2011; Celton et al., 2012) and decreases when NADPH requirements are low (Marx et al., 1999; Jonsbu et al., 2001). This is consistent with the anticipated anabolic role of the oxPPP as a supplier of pentoses and NADPH for biosynthesis. The key enzyme with regard to the control of PPP flux is thought to be G6PDH (Kletzien et al., 1994; Moritz et al., 2000; Saliola et al., 2012). However, in addition to its role in the oxPPP, G6PDH can also participate in the classical phosphorylated version of the Entner–Doudoroff (ED) pathway (Figure 2). The ED pathway can be considered an alternative to the Embden–Meyerhof–Parnas (EMP) glycolytic pathway, because both catabolize glucose to pyruvate (Conway, 1992; Sato and Atomi, 2011). The ED pathway is mainly present in prokaryotes, although some eukaryotes possess a functional ED pathway as well (Fabris et al., 2012). A generally held view is that the ED pathway is less important than the EMP pathway with respect to glucose catabolism. However, Fuhrer et al. have shown that this might be a misconception and that the ED pathway might be a major pathway for glucose catabolism even in species that possess both pathways (Fuhrer et al., 2005).

G6PDHs and 6PGDHs have been widely studied with respect to cofactor specificity. Although some bacterial G6PDHs and 6PGDHs show dual cofactor specificity (Stournaras et al., 1983; Sung and Lee, 2007; Fuhrer and Sauer, 2009) or have a strong preference for NAD^+^ (Stournaras et al., 1983; Sung and Lee, 2007) or other cofactors such as the flavin derivative F_420_ (Purwantini et al., 1997), most are NADP^+^-dependent (Levy, 1979; Fuhrer and Sauer, 2009; Olavarría et al., 2012). In addition, the dual cofactor specificity that some G6PDHs show is often observed *in vitro* under saturated conditions. However, physiological *in vivo* conditions often result in higher specificities for NADP^+^ (Fuhrer and Sauer, 2009). Therefore, G6PDHs and 6PGDHs are generally considered to catalyze the following reactions:

(1) $$\begin{array}{r} \text{glucose-6-phosphate }+\text{ NAD}{\text{P}}^{+}\;\to \\ \text{ 6-phospho-D}-\text{glucono}-1,5-\text{lactone }+\text{ NADPH }+\;{\text{H}}^{+}\\ {\text{Δ}}_{\text{r}}G^{\prime \text{m}}=-2.3\pm 2.6\text{ kJ}∕\text{mol} \end{array}$$

and

(2) $$\begin{array}{r} \text{6-phosphogluconate }+\text{ NAD}{\text{P}}^{+}\to \text{ribulose 5-phosphate }\\ \;+\text{ C}{\text{O}}_{2}\;+\text{ NADPH }+\;{\text{H}}^{+}\\ {\text{Δ}}_{\text{r}}G^{\prime \text{m}}=-6.0\pm 6.3\text{ kJ}∕\text{mol} \end{array}$$

Like eukaryotes, some prokaryotes possess multiple G6PDH (Butler et al., 2002; Singh et al., 2005; Sung and Lee, 2007) or 6PGDH isozymes (Zamboni et al., 2004). In general, these isozymes are similar, with the same cofactor preferences, but exceptions exist. *Mycobacterium smegmatis*, for example, has two G6PDH isozymes, one of which is NADP^+^-dependent. The other is F_420_-dependent and does not utilize NAD^+^ or NADP^+^ (Purwantini and Daniels, 1996). In addition, cell-free extracts of several other *Mycobacterium* and *Nocardia* species contain both F_420_-dependent and NADP^+^-dependent G6PDH activity (Purwantini et al., 1997), indicating that the genomes of these species likely encode both variants. Similarly, exceptions among 6PGDH isozymes have been identified. The genome of *Bacillus subtilis*, for example, encodes three functional 6PGDH isozymes; two prefer NAD^+^, and one prefers NADP^+^ (Zamboni et al., 2004). As discussed by Zamboni et al., the presence of isozymes with different cofactor preferences would, in principle, enable NADP^+^, NAD^+^, and F_420_ reduction in the oxPPP to adjust to the overall metabolic requirements of the cell (Zamboni et al., 2004). However, the exact functional roles of these isozymes remains to be determined.

The availability of NADPH is of principal importance for various industrially important classes of products, including amino acids, proteins, antibiotics, organic acids, and high-value metabolites. The apparent relationship between the rate of NADPH generation and actual carbon fluxes through the oxPPP suggests that the oxPPP can be a target for metabolic engineering for overproduction of NADPH. The oxPPP has been engineered to increase the NADPH/NADP^+^ ratio, through overexpression of oxPPP enzymes (Lim et al., 2002; Choi et al., 2003; Becker et al., 2007; Lee et al., 2007a) and through redirection of the carbon flux from glycolysis to the oxPPP, by disrupting phosphoglucose isomerase (Kabir and Shimizu, 2003; Marx et al., 2003; Chemler et al., 2010) or phosphofructokinase (Chin and Cirino, 2011; Wang et al., 2013d), overexpressing fructose 1,6-bisphosphatase (Becker et al., 2005), or introducing glucose dehydrogenase (Zhang et al., 2011). Both strategies have been applied successfully in various prokaryotes, but reduction in growth is a common side effect (Lim et al., 2002; Lee et al., 2003; Marx et al., 2003). However, the opposite effect was observed with an archaeal strain recently developed in our lab: introduction of the oxPPP increased the hydrogen yield and significantly improved the growth rate (unpublished data).

Although both strategies have been applied successfully in various prokaryotes, they are not effective in every organism (Poulsen et al., 2005; Smith et al., 2010). Moreover, as mentioned above, G6PDH is a key enzyme not only for control of the PPP flux but also for control of the ED pathway. Overexpressing G6PDH could therefore affect the flux of both pathways simultaneously. However, this is generally not observed. Cells apparently possess the ability to regulate fluxes to ensure a network-wide balancing of NADPH supply and demand (Nicolas et al., 2007; Fuhrer and Sauer, 2009).

The effects of G6PDH and 6PGDH disruption have also been investigated. The exact effects are strongly dependent on the organism, the genetic background of the parent strain, and the environmental growth conditions (Summers et al., 1995; Butler et al., 2002; Hua et al., 2003; Zamboni et al., 2004; Zhao et al., 2004b; Richhardt et al., 2013). However, in general, G6PDH-deficient and 6PGDH-deficient prokaryotes exhibit remarkably mild phenotypes. Their growth rates are similar to or somewhat lower than those of the parent strain, and they are more sensitive to oxidative stress. Flux through the oxPPP and ED pathways is lower in G6PDH-deficient strains. To compensate for this and to ensure a sufficient supply of anabolic precursors and NADPH, flux rerouting via the EMP pathway and the non-oxPPP is generally observed (Figure 2). In addition, glucose-grown mutants generally display enhanced TCA cycle activity. In contrast, acetate- or pyruvate-grown cells generally display decreased TCA cycle activity (Zhao et al., 2004a,b). Like G6PDH-deficient strains, 6PGDH-deficient strains exhibit a strong reduction in oxPPP flux, but not in ED pathway flux. In contrast, prokaryotes with a functional ED pathway generally respond to 6PGDH knockout by rerouting through the ED pathway and by reversing the direction of the non-oxPPP, slightly increasing the flux through the EMP pathway, and activating malic enzyme (Jiao et al., 2003; Zhao et al., 2004b). Prokaryotes that lack a functional ED pathway display a similar response, but instead of rerouting the flux through the ED pathway, they increase flux through the EMP pathway (Zamboni et al., 2004). Thus, in general, prokaryotes can compensate for disruption of G6PDH or 6PGDH.

### Isocitrate dehydrogenase

**Figure 3:**
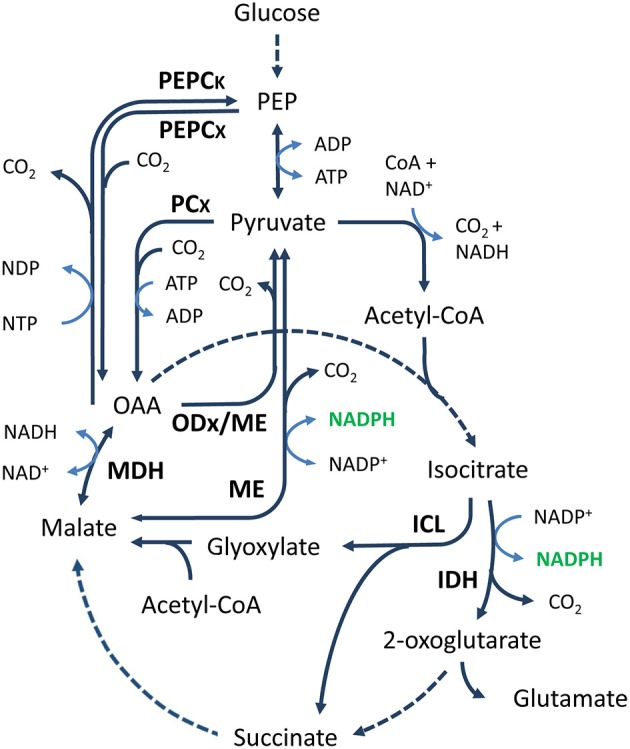
**General overview of the TCA cycle and anaplerotic node**. Solid and dashed arrows represent single and lumped enzymatic reactions, respectively. Malic enzyme is able to catalyze the decarboxylation of malate and OAA. Abbreviations for metabolites (normal text) and enzymes (bold text): PEP, phosphoenolpyruvate; OAA, oxaloacetate; PEPCK, phosphoenolpyruvate carboxykinase; PEPCx, phosphoenolpyruvate carboxylase; PCx, pyruvate carboxylase; ODx, oxaloacetate decarboxylase; MDH, malate dehydrogenase; ME, malic enzyme; ICL, isocitrate lyase; IDH, isocitrate dehydrogenase.

Isocitrate dehydrogenase (IDH, EC:1.1.1.42) is a component of the TCA cycle that catalyzes the decarboxylation of isocitrate to 2-oxoglutarate (also known as α-ketoglutarate), with the release of CO_2_ and NADPH (Figure 3). The IDH reaction is important for the generation of reducing power. Through the generation of 2-oxoglutarate, the reaction also links nitrogen and carbon metabolism and plays an important role in the cellular defense against oxidative damage and detoxification of ROS (Muro-Pastor et al., 2005; Mailloux et al., 2007; Paul et al., 2007). Together with isocitrate lyase, IDH is a branching point between the TCA cycle and the glyoxylate shunt (Figure 3), a pathway needed for growth on non-fermentative carbon sources such as acetate and ethanol. IDH activity is important in controlling the metabolic flux between both pathways and is affected by various regulatory factors, such as metal ions (Murakami et al., 1997; Yoon et al., 2003), PEP concentration (Ogawa et al., 2007), and phosphorylation/dephosphorylation (Walsh and Koshland, 1985; Cozzone, 1998). In addition, IDH is a component of the reductive tricarboxylic acid (RTCA) cycle. The RTCA cycle is a CO_2_ fixation pathway, present in some bacteria and archaea, in which four molecules of CO_2_ are fixed to produce one molecule of oxaloacetate (Shiba et al., 1985; Beh et al., 1993; Kanao et al., 2002). Obviously, IDH cannot act as a source of NADPH under these conditions.

In contrast to eukaryotes, prokaryotes are generally thought to possess only NADP^+^-dependent IDHs. Although this is true for most prokaryotes, a small but increasing number of reports have described bacterial and archaeal NAD^+^-dependent IDHs, such as the one found in *Hydrogenobacter thermophilus* (Lloyd and Weitzman, 1988; Chen and Jeong, 2000; Steen et al., 2001; Inoue et al., 2002; Aoshima et al., 2004; Stokke et al., 2007; Wang et al., 2011b, 2012, 2013a). Moreover, a survey of sequenced prokaryotic genomes by Zhu et al. identified nine additional species that likely contain NAD^+^-dependent IDHs (Zhu et al., 2005). However, although the IDH of *H. thermophilus* was originally described as an NAD^+^-dependent IDH, Aoshima and Igarashi later showed it was not a conventional decarboxylating IDH but a novel non-decarboxylating enzyme that catalyzes the conversion between isocitrate and oxalosuccinate. They therefore proposed that the enzyme should not be categorized as an IDH (Aoshima and Igarashi, 2008).

With the exception of the few NAD^+^-preferring IDHs, prokaryotic IDHs generally catalyze the following decarboxylating reaction:

(3) $$\begin{array}{r} \text{isocitrate }+\text{ NAD}{\text{P}}^{+}\to \text{2-oxoglutarate }+\text{ C}{\text{O}}_{2}\\ \;+\text{ NADPH }+\;{\text{H}}^{+}\\ {\text{Δ}}_{\text{r}}G^{\prime \text{m}}=-10.7\pm 6.3\text{ kJ}∕\text{mol} \end{array}$$

In addition to the cofactor NADP^+^, IDH also needs divalent cations, particularly Mg^2+^ or Mn^2+^. Although most IDHs have a strong requirement for either Mg^2+^ or Mn^2+^, substitution of other divalent cations, or even univalent ions such as Na^+^, is sometimes possible. However, in most cases, this leads to little or no activity (Murakami et al., 1997; Steen et al., 1997; Yoon et al., 2003; Banerjee et al., 2005).

Like G6PDH and 6PGDH, IDH is an important source of NADPH (Hua et al., 2003; Sauer et al., 2004). In wild-type *Escherichia coli* aerobically grown in chemostats under glucose- or ammonia-limited conditions, the IDH reaction was the major producer of NADPH, accounting for more than 60% of the total NADPH production (Hua et al., 2003). Accordingly, overexpression of IDH resulted in a 31% increase in the production of GDP-L-fucose, whose formation is NADPH-dependent. While this is a significant improvement, the biosynthesis of GDP-L-fucose in the IDH overexpression strain was not better than that in a G6PDH overexpression strain (Lee et al., 2011). This suggests that, under the applied conditions, IDH is not a better option than G6PDH for NADPH regeneration in biotransformation reactions (Lee et al., 2013b). The ability of IDH overexpression to enhance glutamate formation in *Corynebacterium glutamicum* has also been tested (Eikmanns et al., 1995). *C. glutamicum* secretes glutamate under certain conditions. Because IDH supplies 2-oxoglutarate, the direct precursor of glutamate, overexpression of IDH was an obvious choice. However, overexpression of IDH, even in combination with glutamate dehydrogenase, did not enhance glutamate production.

Disruption of IDH has also been investigated. The exact effects of IDH disruption were dependent on the specific conditions and species used, but some general trends were apparent (Mcdermott and Kahn, 1992; Muro-Pastor and Florencio, 1994; Eikmanns et al., 1995; Kabir and Shimizu, 2004; Becker et al., 2009). First, all IDH mutants investigated were glutamate auxotrophs. Second, although glutamate-containing cultivation media sustained the growth of IDH mutants, their growth was slower than that of the parental strains. The reduced specific growth rate was most likely due to a lower ATP/ADP ratio and a lower NADPH/NADP^+^ ratio, as shown in an *E. coli* IDH disruption strain grown on glucose under aerobic conditions (Kabir and Shimizu, 2004). Moreover, other significant effects observed in the mutant *E. coli* strain included upregulation of oxPPP enzymes and the anaplerotic glyoxylate pathway. Similarly, redirection from the TCA cycle to anaplerosis has also been reported for an IDH knockdown of *C. glutamicum* (Becker et al., 2009). However, with respect to citrate synthase, contradictory results have been obtained. Kabir and Shimizu (2004) found upregulation of citrate synthase, but an earlier study of *E. coli* IDH mutants (Lakshmi and Helling, 1976) and a study of *Rhizobium meliloti* (Mcdermott and Kahn, 1992) found that IDH mutants spontaneously gave rise to IDH-citrate synthase double mutants.

### Malic enzyme

The TCA cycle-associated malic enzyme (ME, EC:1.1.1.40) catalyzes the NAD(P)^+^-dependent oxidative decarboxylation of malate to pyruvate (Figure 3). Confusingly, malic enzyme is also known as “malate dehydrogenase (oxaloacetate-decarboxylating),” while true malate dehydrogenases, enzymes that catalyze the non-decarboxylating reaction between malate and oxaloacetate (Figure 3), are sometimes referred to as malic enzyme. In addition, various classes of ME that differ in their preference for NAD^+^ or NADP^+^ and in their ability to decarboxylate oxaloacetate have also been characterized (Frenkel, 1975). Enzymes in the first ME class (EC:1.1.1.38) use NAD^+^ and can decarboxylate oxaloacetate as well as malate. Enzymes in the second class (EC:1.1.1.39) also prefer NAD^+^, but they are unable to decarboxylate oxaloacetate. Enzymes in the third class (EC:1.1.1.40) are NADP^+^-dependent; they can catalyze the decarboxylation of malate and oxaloacetate. Moreover, MEs that do not fit the general classification scheme exactly, such as those from the gram-negative bacterium *Rhizobium meliloti* and the hyperthermophilic archaeon *Thermococcus kodakarensis*, have been described as well (Voegele et al., 1999; Fukuda et al., 2005). The latter, for example, prefers NADP^+^ but, unlike normal NADP^+^-dependent MEs, is not able to decarboxylate oxaloacetate.

Although malic enzymes (MEs) are widely distributed amongst prokaryotes (Table 1), most ME-containing species possess an ME that belongs to the third class (Sauer and Eikmanns, 2005; Lerondel et al., 2006) and catalyzes the following reaction:

(4) $$\begin{array}{r} \text{malate }+\text{ NAD}{\text{P}}^{+}\to \text{pyruvate }+\text{ C}{\text{O}}_{2}\;+\text{ NADPH }+\;{\text{H}}^{+}\\ {\text{Δ}}_{\text{r}}G^{\prime \text{m}}=-3.1\pm 6.2\text{ kJ}∕\text{mol} \end{array}$$

However, prokaryotes without an ME or with only an NAD^+^-dependent ME, such as lactic acid bacteria (London et al., 1971; Kawai et al., 1996; Landete et al., 2010; Espariz et al., 2011), or prokaryotes with both NAD^+^- and NADP^+^-dependent MEs have also been characterized (Iwakura et al., 1978; Voegele et al., 1999; Sauer and Eikmanns, 2005; Lerondel et al., 2006; Bologna et al., 2007). Moreover, Morimoto et al. recently showed that directed evolution could easily alter the cofactor preference of an ME from *T. kodakarensis* (Morimoto et al., 2014). Thus, NAD^+^/NADP^+^ specificity seems to depend only on a few specific, highly conserved residues (Kuo et al., 2000; Yang et al., 2002; Chang and Tong, 2003; Fukuda et al., 2005; Lerondel et al., 2006; Hsieh and Hung, 2009).

All MEs require a divalent cation as a cofactor. Maximum activity is generally observed in the presence of Mn^2+^, followed closely by Mg^2+^. Divalent cations such as Co^2+^ and Ni^2+^ are usually only partially able to replace Mn^2+^, while others such as Ca^2+^, Cu^2+^, or Sr^2+^ cannot replace Mn^2+^ at all (Gourdon et al., 2000; Fukuda et al., 2005; Wang et al., 2011a). In addition, the MEs of various prokaryotes also require a monovalent cation for activity, preferably ${\text{NH}}_{4}^{+}$ and K^+^ (Lamed and Zeikus, 1981; Garrido-Pertierra et al., 1983; Kawai et al., 1996; Driscoll and Finan, 1997; Gourdon et al., 2000).

In many aerobic and facultative anaerobic organisms, the TCA cycle-associated ME is part of the PEP-pyruvate-oxaloacetate node, also referred to as the anaplerotic node (Figure 3). This node represents the metabolic link between glycolysis/gluconeogenesis and the TCA cycle (Owen et al., 2002; Sauer and Eikmanns, 2005). As such, ME is involved in the interconversion of C4 and C3 compounds, which is important for maintaining the levels of TCA cycle intermediates (anaplerotic reactions) and for growth on C4 and C3 compounds and substrates that enter central metabolism via acetyl-CoA, such as acetate, fatty acids, and ethanol. Although ME can catalyze C3-carboxylation and C4-decarboxylation reactions, it is generally involved in the latter (decarboxylation of malate to pyruvate with the concomitant formation of NADPH) (Voegele et al., 1999; Gourdon et al., 2000; Sauer and Eikmanns, 2005; Lerondel et al., 2006; Wang et al., 2011a). However, examples of prokaryotic MEs without a preference for either (Fukuda et al., 2005) and MEs with a preference for the C3-carboxylation reaction have been identified (Matula et al., 1969). To determine the exact contribution of ME to the cellular NADPH pool, the physiological direction and flux through the enzyme needs to be established. However, regulation of the carbon flux at the anaplerotic node involves a complex interplay between the enzymes involved and depends on the growth conditions and species (Petersen et al., 2000; Dauner et al., 2001; Fischer and Sauer, 2003; Netzer et al., 2004). A detailed discussion of this complex regulation is beyond the scope of this review, but comprehensive reviews, such as those by Owen et al. (2002) and Sauer and Eikmanns (2005), are available.

To understand the physiological role of ME in the metabolism of prokaryotes, the disruption of ME and alternative pathways has been studied. The significance of the latter is clearly demonstrated by the absence of a detectable phenotype in *Campylobacter jejuni* and *E. coli* ME disruption strains (Velayudhan and Kelly, 2002; Kao et al., 2005). The lack of a clear phenotype can be explained by the presence of alternative pathways and enzymes, such as the malate dehydrogenase-phosphoenolpyruvate carboxykinase pathway (mdh-PEPCK) and isocitrate dehydrogenase, that can fulfill the pyruvate/PEP and NADPH requirements, respectively (Figure 3) (Hansen and Juni, 1975; Oh et al., 2002; Velayudhan and Kelly, 2002; Wang et al., 2011a). Because of the wide distribution of such alternatives in prokaryotes, it is likely that ME gene disruption in other species will yield similar results. Nevertheless, species in which ME gene disruption yields a phenotype, such as *Corynebacterium glutamicum, Bacillus subtilis*, and various lactic acid bacteria, have been identified (Gourdon et al., 2000; Lerondel et al., 2006; Landete et al., 2010; Espariz et al., 2011).

Analysis of various disruption strains created to understand the physiological role of ME in prokaryotes has shown that it is crucial to take the genetic background of a strain into consideration because the exact physiological role of ME differs from species to species (Riedel et al., 2001; Velayudhan and Kelly, 2002; Kao et al., 2005; Landete et al., 2010; Meyer and Stulke, 2013). Analysis has also shown that the effect of ME disruption can be subtle and/or heavily dependent on the cultivation conditions (Gourdon et al., 2000; Lerondel et al., 2006). A *C. glutamicum* ME disruption strain, for example, showed no detectable phenotype during growth on either acetate or glucose, but showed a significantly modified growth behavior during lactate metabolism (Gourdon et al., 2000). Moreover, the physiological role of NAD^+^- and NADP^+^-dependent MEs appears to be different (Lerondel et al., 2006; Bologna et al., 2007; Landete et al., 2010; Espariz et al., 2011; Wang et al., 2011a; Meyer and Stulke, 2013). Whereas NAD^+^-dependent MEs generally play a role in malate catabolism, NADP^+^-dependent MEs function either as gluconeogenic enzymes, by supplying pyruvate from C_4_-dicarboxylic acids, or as NADPH-generating systems needed for various biosynthetic purposes. It is interesting in this respect that the concerted action of ME, MDH, and pyruvate carboxylase (PCx) can act as a kind of transhydrogenase. The combined action of these enzymes enables the ATP-dependent conversion of pyruvate to OAA and malate and back to pyruvate, while converting NADH into NADPH (Figure 3) (Sauer and Eikmanns, 2005). A similar reaction sequence can involve PEP carboxylase (PEPCx) instead of pyruvate carboxylase, which also enables conversion of NADH into NADPH. This so-called malate shunt was recently described in *Clostridium thermocellum* (Zhou et al., 2013).

NAD^+^- and NADP^+^-dependent MEs have also been used in metabolic engineering approaches to improve specific biotechnological applications. However, to our knowledge, no prokaryotic ME has been used to increase intracellular NADPH levels, as has been done in the yeast *Saccharomyces cerevisiae* (Moreira Dos Santos et al., 2004; Matsuda et al., 2013). Instead, prokaryotic MEs have been overexpressed for the formation of C_4_-dicarboxylic acids from glucose or pyruvate (Stols and Donnelly, 1997; Hong and Lee, 2001; Shin et al., 2007; Zheng et al., 2009). These acids are important intermediates in the production of tetrapyrroles (Shin et al., 2007) and amino acids (Sahm et al., 2000), for example, and they can be chemically converted into common intermediates in the petrochemical industry. Hence, C_4_-dicarboxylic acids are important intermediates in the production of commodity chemicals from renewable carbohydrate feedstocks (Jain et al., 1989; Szmant, 1989). However, to produce these acids from glucose or pyruvate, one has to reverse the physiological direction of ME. This has been accomplished in different ways (Stols and Donnelly, 1997; Hong and Lee, 2001; Zheng et al., 2009).

### NADP^+^-dependent glyceraldehyde-3-phosphate dehydrogenases

NADP^+^-dependent glyceraldehyde-3-phosphate dehydrogenases belong to the aldehyde dehydrogenase (ALDH) superfamily, a divergently related group of enzymes widely distributed among all three domains of life (Sophos et al., 2001; Sophos and Vasiliou, 2003). Enzymes in this group metabolize a broad spectrum of endogenous and exogenous aldehydes, which are oxidized to the corresponding carboxylic acid, using NAD^+^ or NADP^+^ as a cofactor (Lindahl, 1992; Vasiliou et al., 2000):

$$\begin{array}{l} \text{RCHO }\left(\text{aldehyde}\right)\;+\text{ NAD}{\text{P}}^{+}\;+\;{\text{H}}_{2}\text{O}\to \text{RCOOH }\left(\text{acid}\right)\\ \;+\text{ NADPH }+\;{\text{H}}^{+} \end{array}$$

As such, the enzymes play essential roles in processes such as intermediary metabolism and detoxification (Talfournier et al., 2011; Esser et al., 2013). Although many ALDHs are NADP^+^-dependent, only the NADP^+^-dependent glyceraldehyde-3-phosphate dehydrogenases will be addressed here, because these enzymes contribute significantly to NADPH levels in certain prokaryotes. Moreover, extensive reviews of the ALDH superfamily are available (Sophos et al., 2001; Sophos and Vasiliou, 2003; Marchitti et al., 2008; Jackson et al., 2011).

**Figure 4:**
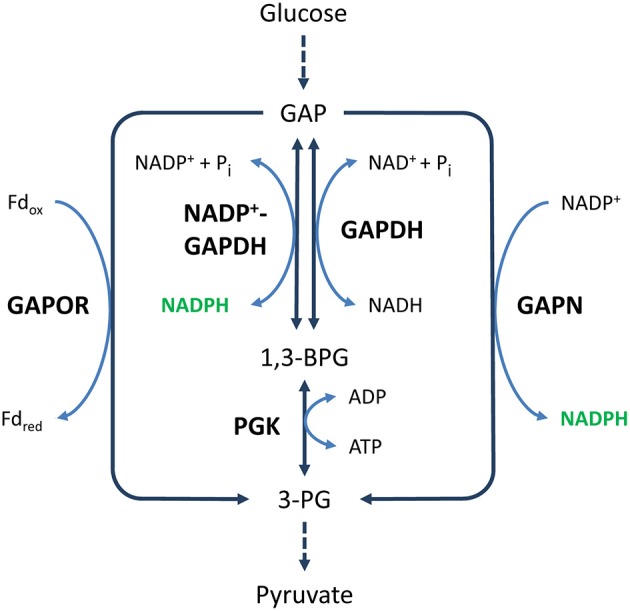
**NADPH generation by GAP dehydrogenases**. Solid and dashed arrows represent single and lumped enzymatic reactions, respectively. Abbreviations for metabolites (normal text) and enzymes (bold text): GAP, glyceraldehyde 3-phosphate; 1,3-BPG, 1,3-bisphosphoglycerate; 3-PG, 3-phosphoglycerate; GAPOR, GAP:ferredoxin oxidoreductase; GAPDH, glyceraldehyde-3-phosphate dehydrogenase; GAPN, non-phosphorylating GAPDH; PGK, phosphoglycerate kinase.

While most glyceraldehyde-3-phosphate dehydrogenases (GAPDH, EC:1.2.1.12) are NAD^+^-dependent enzymes responsible for the reversible oxidation of GAP into 1,3-bisphosphoglycerate (1,3-BPG) as part of the classical EMP pathway, NADP^+^-dependent variants exist as well. In fact, there are two different types of NADP^+^-dependent GAPDHs, a phosphorylating type (NADP^+^-GAPDH, EC:1.2.1.13), producing 1,3-BPG, and a non-phosphorylating type (GAPN, EC:1.2.1.9), unidirectionally producing 3-phosphoglycerate (3-PG) (Figure 4). However, the term NADP^+^-GAPDH is used inconsistently in different studies to denote either the phosphorylating or non-phosphorylating type or both types, thus generating confusion.

GAPN was originally thought to be present only in photosynthetic eukaryotes (Kelly and Gibbs, 1973; Mateos and Serrano, 1992). However, early reports of GAPN activity in *Streptococcus mutans* were later confirmed by the characterization of the first prokaryotic GAPN (Crow and Wittenberger, 1979; Boyd et al., 1995). Since then, GAPN has also been found in various (hyper)thermophilic archaea, such as *Thermococcus kodakarensis, Thermoproteus tenax*, and *Sulfolobus solfataricus* (Brunner et al., 1998; Ettema et al., 2008; Matsubara et al., 2011), and several gram-positive bacteria, such as *Clostridium acetobutylicum, Streptococcus pyogenes*, and *Bacillus halodurans* (Table 1) (Iddar et al., 2002, 2003, 2005; Sophos and Vasiliou, 2003; Agledal et al., 2010). GAPN catalyzes the irreversible, non-phosphorylating oxidation of GAP to 3-PG with the concurrent reduction of NADP^+^ to NADPH:

(5) $$\begin{array}{r} \text{GAP }+\text{ NAD}{\text{P}}^{+}\;+\;{\text{H}}_{2}\text{O}\to \text{3-phosphoglycerate }+\text{ NADPH}\\ \;+\;{\text{H}}^{+}\\ {\text{Δ}}_{\text{r}}G^{\prime \text{m}}=-36.1\pm 1.1\text{ kJ}∕\text{mol} \end{array}$$

In this reaction, 1,3-BPG is not formed, and the oxidation of GAP is not coupled to the generation of ATP (Figure 4). Such modifications of the classical EMP or ED pathway are regularly found in archaea (Ahmed et al., 2005; Siebers and Schonheit, 2005; Ettema et al., 2008; Matsubara et al., 2011; Brasen et al., 2014). Interestingly, GAPN has been reported to have a key function in the regulation of these modified pathways (Brunner et al., 1998; Brunner and Hensel, 2001; Lorentzen et al., 2004; Ettema et al., 2008).

With the exception of the GAPN in *T. tenax*, which has a preference for NAD^+^, prokaryotic GAPNs, in general, have a clear preference for NADP^+^. However, although the GAPN of *T. tenax* was shown to be NAD^+^-dependent, it was later shown to be allosterically regulated, shifting its cofactor preference from NAD^+^ to NADP^+^ in the presence of activators such as F6P and G1P (Lorentzen et al., 2004). Based on sequence similarity, similar allosteric regulation might also exist for GAPNs in a number of other hyperthermophiles (Lorentzen et al., 2004; Ito et al., 2012). Aside from NADP^+^, GAPNs do not require other cofactors or metals.

While the physiological significance of GAPN might vary between organisms, the enzyme is an important and essential source of NADPH in the bacterium *S. mutans* and the archaeon *T. kodakarensis*, respectively (Boyd et al., 1995; Matsubara et al., 2011; Arutyunov et al., 2013). Moreover, the ability to use GAPN as a source of NADPH has been demonstrated in a mutant *E. coli* strain in which the endogenous NAD^+^-dependent glyceraldehyde-3-phosphate dehydrogenase (NAD^+^-GAPDH) was replaced by the GAPN of *S. mutans*. Transcriptional analysis of this strain revealed upregulation of the transhydrogenase and downregulation of the PPP and TCA cycle, which together indicate a response to avoid NADPH excess (Centeno-Leija et al., 2013). In addition, GAPN has been used successfully in metabolic engineering efforts intended to improve the production of metabolites whose biosynthesis depends on NADPH, such as L-lysine or poly-3-hydroxybutyrate, or to decrease the formation of undesired by-products such as glycerol in industrial ethanol production strains (Takeno et al., 2010; Guo et al., 2011; Centeno-Leija et al., 2014).

In contrast to GAPN, NADP^+^-GAPDH produces 1,3-BPG. It is therefore more similar to NAD^+^-GAPDH (Figure 4).

(6) $$\begin{array}{r} \text{GAP }+\text{ NAD}{\text{P}}^{+}\;+\text{ Pi}\to 1,3-\text{bisphosphoglycerate}\\ \;+\text{ NADPH }+\;{\text{H}}^{+}\\ {\text{Δ}}_{\text{r}}G^{\prime \text{m}}=25.9\pm 1.0\text{ kJ}∕\text{mol} \end{array}$$

The generation of 1,3-BPG is an important difference between the reactions catalyzed by NADP^+^-GAPDH and GAPN, because further oxidation of 1,3-BPG to 3-PG generates ATP. Moreover, while the oxidation of GAP by NADP^+^-GAPDH is reversible, the oxidation by GAPN is not. Hence, NADP^+^-GAPDH, in contrast to GAPN, is active in both glycolysis and gluconeogenesis. However, in prokaryotes that also contain alternative GAP oxidation enzymes, such as NAD^+^-GAPDH, GAPN, or GAP:ferredoxin oxidoreductase (GAPOR) (Figure 4), NADP^+^-GAPDH is primarily involved in gluconeogenesis and does not appear to play a role in generating NADPH (Schäfer and Schönheit, 1993; Koksharova et al., 1998; Fillinger et al., 2000; Brunner et al., 2001; Matsubara et al., 2011; Ito et al., 2012).

As with GAPN, the significance of NADP^+^-GAPDH as a source of NADPH has been demonstrated. Overexpression of NADP^+^-GAPDH or replacement of the native NAD^+^-GAPDH with the NADP-variant improved the yield of NADPH-dependent products such as lycopene, ϵ-caprolactone, L-ornithine, or coenzyme Q_10_ (Martinez et al., 2008; Huang et al., 2011; Jiang et al., 2013; Wang et al., 2013c). Moreover, metabolic flux analysis of the exchange mutant revealed that the oxPPP branch and TCA fluxes were significantly reduced, presumably to avoid NADPH excess (Martinez et al., 2008). In addition, an attempt in *C. glutamicum* to alter the cofactor specificity of native NAD^+^-GAPDH to NADP^+^ through protein engineering resulted in a 60% increase in lysine production (Bommareddy et al., 2014).

### NADP^+^-dependent glucose dehydrogenase

**Figure 5:**
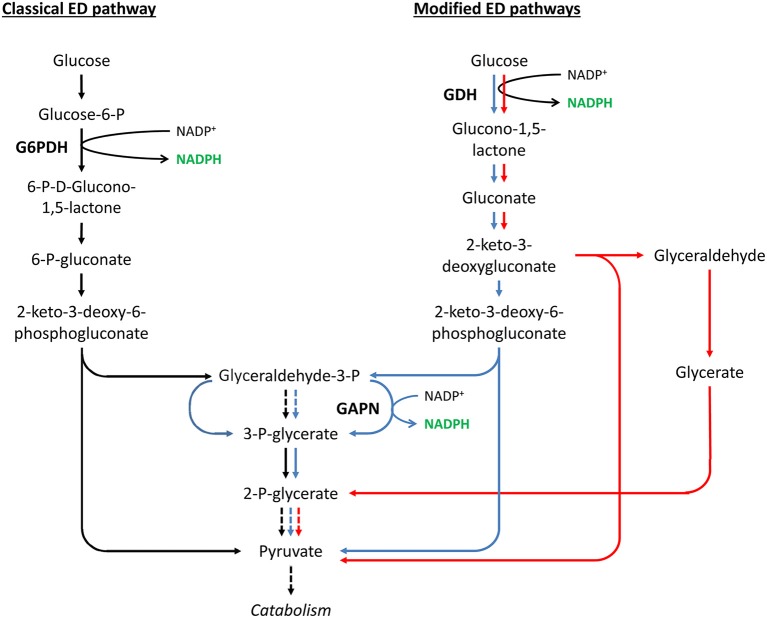
**Classical ED pathway compared to modified ED pathways**. Solid and dashed arrows represent single and lumped enzymatic reactions, respectively. Shown are the classical ED pathway (in black), the semiphosphorylated ED pathway (in blue), the non-phosphorylated ED pathway (in red), and the branched ED pathway that includes the semi- and non-phosphorylated ED pathways. Only the glucose degradation directions are shown. Abbreviated enzymes (in bold text): G6PDH, glucose-6-phosphate dehydrogenase; GAPN, non-phosphorylating glyceraldehyde-3-phosphate dehydrogenase; GDH, glucose dehydrogenase.

Similar to GAPN and GAPDH, other non-canonical NADP^+^-dependent enzymes, such as NADP^+^-dependent glucose dehydrogenase (NADP^+^-GDH), might make a larger contribution to NADPH generation than previously thought. Glucose dehydrogenases (GDHs, EC:1.1.1.47 and EC:1.1.1.119) are responsible for the first step of the modified ED pathways found in some archaea and bacteria (Table 1), i.e., the semiphosphorylated ED, the non-phosphorylated ED, and the branched ED pathways (Figure 5) (Brasen et al., 2014). GDHs are responsible for a main difference between modified ED pathways and the classical ED pathway. GDHs catalyze the direct oxidation of glucose into gluconolactone. In contrast, in the classical ED, glucose is phosphorylated before its oxidation into 6-P-gluconolactone (Brasen et al., 2014).

(7) $$\begin{array}{r} \text{L}-\text{glucose }+\text{ NAD}{\text{P}}^{+}\to \text{L-glucono-1,5-lactone }+\text{ NADPH}\\ \;+{\text{H}}^{+}\\ {\text{Δ}}_{\text{r}}G^{\prime \text{m}}=-2.4\pm 2.2\text{ kJ}∕\text{mol} \end{array}$$

Two different classes of GDHs are capable of using NADP^+^ as cofactor: EC:1.1.1.47, which shows dual cosubstrate specificity with a preference for NADP^+^, and EC:1.1.1.119, which is strictly dependent on NADP^+^. GDHs belonging to these groups have been found in extremely halophilic and thermoacidophilic archaea, as well as in bacteria such as *Bacillus subtilis, Bacillus megaterium*, and *Gluconobacter oxydans* (Pauly and Pfleiderer, 1975; Adachi and Ameyama, 1981; Lampel et al., 1986; Brasen et al., 2014).

NADP^+^-GDHs have been used for NADPH regeneration in various production systems, such as the *in vitro* synthesis of poly(3-hydroxybutyrate) or L-leucovorin (Eguchi et al., 1992; Satoh et al., 2003). In addition, NADP^+^-GDHs are important components of many commercial glucose-sensing assays. As such, they have been utilized for blood glucose monitoring (Ferri et al., 2011). Moreover, NADP^+^-GDHs have also been used as NADPH regenerators in various whole-cell, or *in vivo*, production systems. The GDHs of *B. megaterium* and *B. subtilis*, for example, have been expressed in several *E. coli* strains for the synthesis of various products, including indigo, *S*-sulfoxide, and ethyl (R/S)-4-chloro-3-hydroxybutanoate (Kataoka et al., 1998, 1999; Kizaki et al., 2001; Yun et al., 2005; Lu and Mei, 2007; Xu et al., 2007; Park et al., 2010; Zhang et al., 2011).

## NADPH-generating reactions not coupled to carbon metabolism

### Transhydrogenases

**Figure 6:**
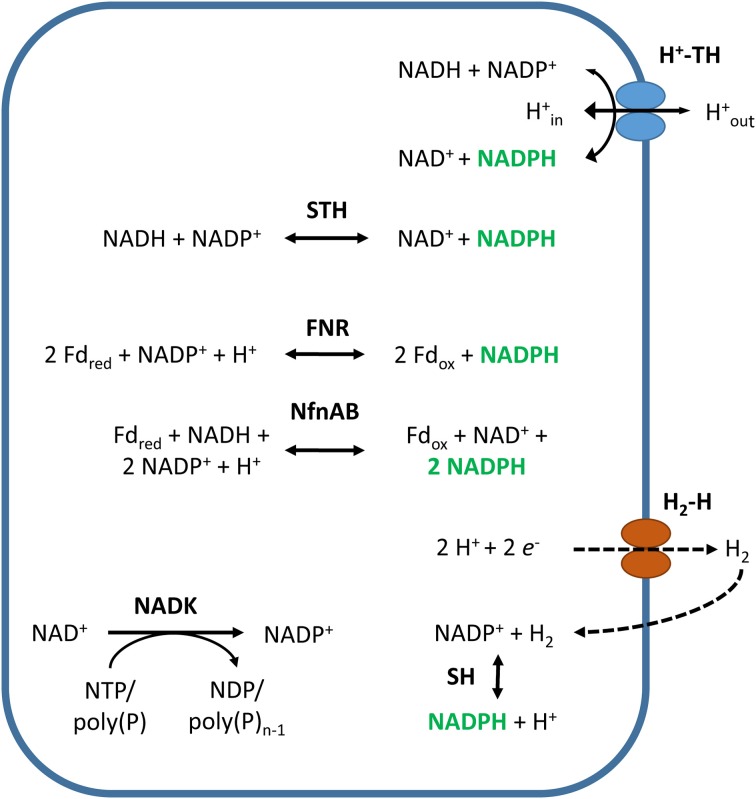
**NADPH-generating reactions not coupled to carbon metabolism**. Abbreviated enzymes (in bold): H_2_-H, hydrogen-evolving hydrogenase; SH, cytosolic NADP^+^-reducing hydrogenase; NfnAB, electron-bifurcating NADH-dependent reduced ferredoxin:NADP^+^ oxidoreductase; FNR, ferredoxin:NADP^+^ oxidoreductase; STH, energy-independent soluble transhydrogenase; H^+^-TH, energy-dependent or proton-translocating, membrane-bound transhydrogenase.

Pyridine nucleotide transhydrogenase (TH) directly catalyzes the reversible hydride transfer between NAD(H) and NADP(H). There are two different isoforms: the energy-independent soluble transhydrogenase (STH, EC:1.6.1.1) and the energy-dependent, or proton-translocating, membrane-bound transhydrogenase (H^+^-TH, EC:1.6.1.2) (Figure 6). Soluble transhydrogenases from *Gammaproteobacteria* such as *Escherichia coli, Azotobacter vinelandii, Pseudomonas fluorescens*, and *Pseudomonas aeruginosa* have been studied in some detail (Cohen and Kaplan, 1970; Voordouw et al., 1979; French et al., 1997; Boonstra et al., 1999). In contrast, proton-translocating, membrane-bound transhydrogenases have been investigated extensively, reviewed in (Bizouarn et al., 2000, 2002; Jackson, 2003, 2012; Pedersen et al., 2008; Jackson and Obiozo, 2009; Lee et al., 2013b). They are widely distributed in the mitochondria of eukaryotes and in certain bacteria, but are very rare in archaea (Table 1) (Jackson and Obiozo, 2009; Jackson, 2012). Bacteria with multiple copies of the transhydrogenase genes are not uncommon, and some species, particularly among the *Enterobacteriaceae*, contain both isoforms (Sauer et al., 2004).

The two transhydrogenase isoforms transfer electrons between NAD(H) and NADP(H) in the following reaction:

(8) $$\begin{array}{r} \text{NADH }+\text{ NAD}{\text{P}}^{+}↔\text{NA}{\text{D}}^{+}\;+\text{ NADPH}\\ {\text{Δ}}_{\text{r}}G^{\prime \text{m}}=1.0\pm 0.7\text{ kJ}∕\text{mol} \end{array}$$

However, in contrast to STHs, H^+^-THs couple the electron transfer reaction to the translocation of a proton across the membrane, according to the reaction:

(9) $$\begin{array}{r} {\text{H}}_{\text{out}}^{+}+\text{NADH }+\text{ NAD}{\text{P}}^{+}↔{\text{H}}_{\text{in}}^{+}\;+\text{ NA}{\text{D}}^{+}\;+\text{ NADPH} \end{array}$$

Where “out” and “in” denote the periplasmic space and cytosol, respectively. Although the reaction is in principle reversible, the electrochemical proton gradient (Δ*p*) across the membrane under most physiological conditions strongly favors NADPH formation (Hoek and Rydstrom, 1988; Bizouarn et al., 2000; Jackson, 2003; Pedersen et al., 2008).

Since the discovery of transhydrogenases, there has been much debate about their physiological role (Bragg et al., 1972; Voordouw et al., 1983; Harold, 1986; Hoek and Rydstrom, 1988; Jackson et al., 1998; Pedersen et al., 2008). Given the type of reaction they catalyze, transhydrogenases might play an important role in maintaining the redox balance within microorganisms. Various studies have shown that several bacterial species require STH for growth under metabolic conditions with excess NADPH formation (Canonaco et al., 2001; Hua et al., 2003; Sauer et al., 2004; Zhao et al., 2008; Fuhrer and Sauer, 2009). Moreover, mutant bacteria suffering from a disturbed redox balance due to an increased NADPH concentration often benefit from the heterologous expression of STH (Boonstra et al., 2000; Angermayr et al., 2012; Qi et al., 2014; Reddy et al., 2015). These observations support the idea that the physiological role of STH is to convert NADPH into NADH to prevent an excess of NADPH (Voordouw et al., 1983), a notion that is generally accepted and is supported by actual NAD^+^/NADH and NADP^+^/NADPH ratios (see above), which indicate that the formation of NADPH from NADH will generally require energy input.

However, Sanchez et al. have reported that the NADPH-requiring production of poly(3-hydroxybutyrate) in *E. coli* benefitted from the overexpression of STH (Sanchez et al., 2006). This result is contrary to the generally accepted physiological role of STH and suggests that the enzyme catalyzes the opposite reaction. Moreover, recent reports have shown that STH is able to catalyze the opposite reaction and that overexpression of the enzyme can improve NADPH-dependent production yields (Lee et al., 2010; Decorosi et al., 2011; Jan et al., 2013; He et al., 2014). Additionally, although STH disruption and overexpression mutants often have disturbed or improved redox balances, respectively, similar mutants that do not show any effect have also been described (Chin et al., 2009; Yamauchi et al., 2014). Together, these examples show that we have yet to achieve a thorough understanding of the physiological role of STH and that its role is strongly dependent on species, culture conditions, and genotypes.

Similarly, the physiological role of H^+^-TH has been debated in the literature. It has often been reported that the H^+^-TH isoform is a major source of NADPH, needed for the biosynthesis of amino acids, for example, or the reduction of glutathione (required to minimize oxidative damage caused by free radicals generated in the respiratory chain) (Bragg et al., 1972; Voordouw et al., 1983; Hoek and Rydstrom, 1988; Hickman et al., 2002; Hua et al., 2003; Sauer et al., 2004; Fuhrer and Sauer, 2009; Imam et al., 2013; He et al., 2014). In *E. coli*, H^+^-TH has been shown to provide about 40% of the total NADPH during growth on glucose in batch cultures (Sauer et al., 2004). Moreover, its expression is induced when there is demand for NADPH, and it is required for optimal growth on carbon sources whose metabolism does not directly generate NADPH (Sauer et al., 2004; Imam et al., 2013). Additionally, Fuhrer and Sauer calculated that it is very unlikely, from a thermodynamic point of view, that H^+^-THs catalyze NADPH oxidation. They concluded that the NADP^+^ reduction catalyzed by H^+^-THs is irreversible under physiological conditions in various bacterial species (Fuhrer and Sauer, 2009). Reports such as these contribute to the general view that the physiological role of H^+^-TH is reduction of NADP^+^ at the expense of NADH. However, other reports suggest that H^+^-TH catalyzes the opposite reaction or show that disruption or overexpression has no measurable effect (Chin et al., 2009; Jan et al., 2013; Yamauchi et al., 2014).

THs require the cofactors NAD(H) and NADP(H) for activity. In addition, and in contrast to H^+^-THs, STHs also require a flavin (flavin adenine dinucleotide, FAD) cofactor (Middleditch et al., 1972; Boonstra et al., 1999; Cao et al., 2011). THs generally do no required other cofactors or metal ions. In fact, metal ions have even been reported to inhibit TH activity (Whitehead et al., 2005, 2009; Cao et al., 2011). Nevertheless, submillimolar concentrations of Ca^2+^ and Mg^2+^ have been reported to stimulate the H^+^-THs of *Rhodobacter capsulatus* and *E. coli* (Cotton et al., 1989; Lever et al., 1991; Chang et al., 1992). Similarly, saturating concentrations of Ca^2+^ have been reported to release the phosphate-induced inhibition of the NADP^+^ reducing reaction and to cause full activation of the enzyme in *Azotobacter vinelandii* and *Pseudomonas aeruginosa*, respectively (Hojeberg and Rydstrom, 1977; Voordouw et al., 1980).

Because of their ability to generate NADPH, STHs and H^+^-THs have been overexpressed to improve the production yields of NADPH-dependent metabolites. For example, overexpression of STH increased NADPH-dependent poly(3-hydroxybutyrate) production by 82% in an engineered *E. coli* strain (Sanchez et al., 2006). Moreover, the overexpression of STH in two other engineered *E. coli* strains increased (S)-2-chloropropionate and thymidine production 150 and 100%, respectively (Lee et al., 2010; Jan et al., 2013). Similarly, overexpression of H^+^-TH has significantly improved the yields of various NADPH-dependent products, such as chiral alcohols, isobutanol, and 3-hydroxypropionic acid in *E. coli* (Weckbecker and Hummel, 2004; Bastian et al., 2011; Rathnasingh et al., 2012; Shi et al., 2013) and L-valine and L-lysine in *C. glutamicum* (Kabus et al., 2007; Bartek et al., 2011).

### Ferredoxin:NADP^+^ oxidoreductase

Ferredoxin:NADP^+^ oxidoreductases (FNRs, EC:1.18.1.2) are ubiquitous flavoenzymes (Table 1) that catalyze the reversible transfer of reducing equivalents between the one-electron carrier ferredoxin (Fd) and the two-electron carrying NADP(H), according to the following reaction (Figure 6):

(10) $$\begin{array}{r} 2\text{F}{\text{d}}_{\text{red}}\;+\text{ NAD}{\text{P}}^{+}\;+\;{\text{H}}^{+}↔2\text{F}{\text{d}}_{\text{ox}}\;+\text{ NADPH}\\ {\text{Δ}}_{\text{r}}G^{\prime \text{m}}=-15.6\pm 11.7\text{ kJ}∕\text{mol} \end{array}$$

FNRs are present in all three domains of life (Ma and Adams, 2001a; Ceccarelli et al., 2004; Santangelo et al., 2011; Yan et al., 2014). They are mainly known for their essential role in photosynthetic organisms (plants, algae, and cyanobacteria), in which they connect the Fd_red_-generating reactions of photosynthesis to NADPH-requiring carbon assimilation (Shin and Arnon, 1965; Goss and Hanke, 2014). In addition to enabling the flux of electrons from the photosystem into other metabolic pathways, FNRs might also avert this efflux of electrons and mediate the return of Fd-bound electrons to the plastoquinone pool of the photosynthetic system. By doing so, FNRs contribute to the so-called cyclic electron flow, which is essential for balancing the ATP/NADPH ratio generated by the photosystems (Munekage et al., 2004; Shikanai, 2007; Kramer and Evans, 2011; Mulo, 2011; Goss and Hanke, 2014).

In non-photosynthetic tissues and organisms, FNRs participate in the electron transfer chains of various other metabolic processes such as nitrogen fixation, sulfate assimilation, isoprenoid biosynthesis, osmotic and oxidative stress responses, and iron-sulfur cluster biogenesis (Arakaki et al., 1997; Krapp et al., 2002; Seeber et al., 2005; Giro et al., 2006; Lee et al., 2007b; Balconi et al., 2009; Tang et al., 2010; Lewis et al., 2013). However, in contrast to the FNRs involved in photosynthesis, the physiological direction of FNRs involved in these processes is toward the production of Fd_red_. As a result, FNRs are sometimes classified as autotrophic (all photosynthetic FNRs) and heterotrophic (all other FNRs) (Arakaki et al., 1997), which, from a protein-family point of view, is not entirely correct.

FNRs are generally classified into two phylogenetically and structurally unrelated protein families that are subdivided into four subclasses: the plant-type FNRs comprising the plastidic-type and bacterial-type subclasses and the glutathione (GR)-type FNRs comprising the mitochondrial adrenodoxin reductases (AdR-like) and oxygenase-coupled NADH:ferredoxin reductases (ONFR-like) (Carrillo and Ceccarelli, 2003; Ceccarelli et al., 2004; Karplus and Faber, 2004; Aliverti et al., 2008; Musumeci et al., 2012). In addition, a third type of FNR has been reported, the so-called thioredoxin reductase-like FNR (TRLF), which functions as an FNR but has structural homology to NADPH-dependent thioredoxin reductases (Seo and Sakurai, 2002; Seo et al., 2004; Komori et al., 2010; Muraki et al., 2010; Yan et al., 2014). Interestingly, a novel type of FNR has recently been reported, the electron-bifurcating NADH-dependent reduced ferredoxin:NADP^+^ oxidoreductase (NfnAB), which couples the exergonic reduction of NADP^+^ with Fd_red_ and the endergonic reduction of NADP^+^ with NADH in a reversible reaction (reaction 11 and Figure 6) (Wang et al., 2010; Huang et al., 2012; Rydzak et al., 2014). Genome analyses have revealed the *NfnAB* genes are present in many anaerobic bacteria and archaea (Huang et al., 2012; Buckel and Thauer, 2013), a finding that might reflect the importance of a postulated NfnAB function: NfnAB and other electron-bifurcating reactions have been implicated as a third mode of energy conservation, in addition to substrate level phosphorylation and electron transport phosphorylation (Buckel and Thauer, 2013).

(11) $$\begin{array}{r} \text{F}{\text{d}}_{\text{red}}\;+\text{ NADH }+\;2\text{NAD}{\text{P}}^{+}\;+\;{\text{H}}^{+}↔\text{F}{\text{d}}_{\text{ox}}\;+\text{ NA}{\text{D}}^{+}\\ \;+2\text{NADPH} \end{array}$$

Despite what the names imply, both subdivisions of the plant-type FNRs (plastidic-type and bacterial-type) as well as FNRs of the GR-type, TRLF-type, and “NfnAB-type” are found in prokaryotes (Ceccarelli et al., 2004; Aliverti et al., 2008; Komori et al., 2010). Unfortunately, the literature does not always make clear the class to which an FNR belongs, generating confusion and making it difficult to judge the true NADPH-generating potential of FNRs found in different prokaryotes. Nevertheless, under normal physiological conditions, it appears that only the plastidic-type FNRs, found in cyanobacteria, for example, and the novel “NfnAB-type” have significant effects on NADPH availability in prokaryotes.

The reaction catalyzed by the different FNR types is in principle reversible, as demonstrated by various *in vitro* studies, as well as by *in vivo* studies in cyanobacteria and the thermophilic bacterium *Hydrogenobacter thermophilus* TK-6. Cyanobacteria contain a single FNR that is responsible for NADP^+^ reduction in vegetative cells as well as for Fd reduction in heterocysts (Razquin et al., 1996). A similar situation exists in *H. thermophilus* TK-6, where a single FNR catalyzes either the forward or reverse reaction depending on the specific type of Fd involved in the reaction. A [4Fe-4S]-containing Fd is involved in the forward (NADP^+^ reducing) reaction, whereas a [2Fe-2S]-containing Fd is involved in the reverse reaction (Ikeda et al., 2009). Such insights might facilitate the development of rational engineering approaches aimed at gaining directional control of the reaction. Elucidating the reaction mechanisms of various FNR-types is a topic of many studies and has been reviewed extensively (Arakaki et al., 1997; Onda et al., 2000; Aliverti et al., 2001, 2008; Carrillo and Ceccarelli, 2003; Medina and Gomez-Moreno, 2004; Martinez-Julvez et al., 2009; Yeom et al., 2009; Musumeci et al., 2012).

FNRs, in general, contain a non-covalently bound flavin (FAD) cofactor as a prosthetic group, but a flavin mononucleotide (FMN)-containing FNR has also been characterized (Ikeda et al., 2009). The FAD group can alternate between an oxidized, one-electron-reduced semiquinone and a fully reduced hydroquinone state, thereby mediating the reversible electron change between the one-electron carrier Fd and the two-electron carrying NADP(H) (Carrillo and Ceccarelli, 2003). The iron-sulfur-containing Fd can sometimes be substituted by the flavin mononucleotide (FMN)-containing flavodoxin (under iron-depleted conditions in some bacteria) or, in the case of the GR-type FNRs, by the mitochondrial iron-sulfur-containing adrenodoxin (Yamazaki and Ichikawa, 1990; Ceccarelli et al., 2004; Sancho, 2006; Ewen et al., 2011). Moreover, a novel rubrerythrin-like protein, named ferriperoxin (Fpx), has been described that exhibits NADPH- and FNR-dependent peroxidase activity in the thermophilic hydrogen-oxidizing bacterium *H. thermophilus* (Sato et al., 2012). Whereas most FNRs are NADP(H)-dependent, an NAD(H)-dependent variant also exists, the ONFR-like subtype of the GR-type FNRs (EC:1.18.1.3) (Aliverti et al., 2008). However, because this group of FNRs is not involved in NADPH availability, it will not be discussed further in this review.

### NADP^+^-dependent hydrogenase

Hydrogenases (H_2_ases) are metalloenzymes that catalyze the reversible oxidation of hydrogen gas into two protons and two electrons:

$$\begin{array}{l} {\text{H}}_{2}↔2{\text{H}}^{+}\;+\;2{\text{e}}^{-} \end{array}$$

H_2_ases enable organisms to utilize H_2_ as a source of reducing power or to use protons as terminal electron acceptors, generating H_2_. According to the metal atoms at their active site, H_2_ases can be classified into three types: [NiFe]-H_2_ases, [FeFe]-H_2_ases, and [Fe]-H_2_ases. The latter were formerly known as metal-free or iron-sulfur-free H_2_ases; they are involved in CO_2_ reduction with H_2_ to methane and are only found in methanogenic archaea (Lyon et al., 2004; Vignais and Colbeau, 2004; Shima and Thauer, 2007; Trchounian et al., 2012; Trchounian and Gary Sawers, 2014; Peters et al., 2015). [NiFe]-H_2_ases are generally responsible for H_2_ oxidation and are widely distributed among both aerobic and anaerobic bacteria and archaea; they have been identified in 28.9 and 57.8% of the available complete bacterial and archaeal genomes, respectively. [FeFe]-H_2_ases, in contrast, are generally responsible for H_2_ evolution and are only found in anaerobic bacteria; they have been identified in 9.8% of the available complete bacterial genomes (Peters et al., 2015). Alignment of the sequence motifs that coordinate the active site, separates [NiFe]-H_2_ases into at least four, and possibly five, functional groups that are, in general, consistent with the physiological roles of the enzymes (Vignais et al., 2001; Vignais and Colbeau, 2004; Vignais and Billoud, 2007; Peters et al., 2015). Thus, H_2_ases are a diverse group of enzymes. They are cytosolic or membrane bound, involved in H_2_ oxidation and/or evolution, and able to use a variety of electron acceptors and donors. It is beyond the scope of this review to address all of the different types of H_2_ases. We will focus on those involved in NADPH generation in prokaryotes. For more information about H_2_ases in general, the reader is referred to various excellent reviews, such as those by Vignais and Colbeau (2004), Shima and Thauer (2007), and Peters et al. (2015).

H_2_ases that use the cofactor NADP^+^ as an electron acceptor are not common, but have been identified in some bacteria and archaea (Table 1). Cytosolic NADP^+^-reducing hydrogenases (EC:1.12.1.3, SH) are present in the sulfate-reducing bacterium *Desulfovibrio fructosovorans* and the hyperthermophilic archaea *Pyrococcus furiosus* and *Thermococcus kodakarensis* (Malki et al., 1995; Kanai et al., 2003; Nouailler et al., 2006; Van Haaster et al., 2008). Moreover, cytosolic bidirectional NAD(P)^+^-H_2_ases and a membrane-bound NAD(P)^+^-H_2_ase, known to use both NAD^+^ and NADP^+^ as substrates, have been found in cyanobacteria and *Klebsiella pneumonia*, respectively (Steuber et al., 1999; Schmitz et al., 2002; Wells et al., 2011). Many of these H_2_ases recycle the H_2_ produced by H_2_-evolving H_2_ases by coupling the oxidation of H_2_ to the reduction of NADP^+^ (Figure 6) (Steuber et al., 1999; Silva et al., 2000; Van Haaster et al., 2008; Kanai et al., 2011; Santangelo et al., 2011; Schut et al., 2012), according to the following reaction:

(12) $$\begin{array}{r} {\text{H}}_{2}\;+\text{ NAD}{\text{P}}^{+}↔{\text{H}}^{+}\;+\text{ NADPH}\\ {\text{Δ}}_{\text{r}}G^{\prime \text{m}}=-16.5\pm 5.9\text{ kJ}∕\text{mol} \end{array}$$

H_2_ recycling might have a distinct energetic advantage because the NADP^+^-reducing H_2_ase could provide reductant in the form of NADPH without interfering with the energy balance through electron transport phosphorylation (Schut et al., 2012). However, various studies have shown that disruption of NADP^+^-reducing H_2_ases has minor effects, suggesting that the role of the enzymes is not that important for overall growth (Casalot et al., 2002; Kanai et al., 2011; Lipscomb et al., 2011; Santangelo et al., 2011; Schut et al., 2012; Lauterbach et al., 2013).

Like most H_2_ases, NADP^+^-reducing H_2_ases require metal ions and cofactors for their activity. They contain multiple iron–sulfur clusters ([Fe–S]), which are coordinated in the active site by carbon monoxide (CO) and cyanide (CN^−^) groups. Moreover, various H_2_ases capable of reducing NADP^+^ belong to the [NiFe]-H_2_ases, making the availability of Ni essential as well (Ma and Adams, 2001b; Kanai et al., 2003; Germer et al., 2009; Peters et al., 2015). In addition, the enzymes have been reported to contain flavin cofactors: FAD in the case of *T. kodakarensis* and *P. furiosus* (Ma et al., 2000; Ma and Adams, 2001b; Kanai et al., 2003) and FMN in the case of *D. fructosovorans* (Malki et al., 1995).

The requirement for these different ligands, metals, and cofactors makes the biosynthesis of multisubunit H_2_ases a complex and dynamic process, involving various maturation proteins (Bock et al., 2006; Forzi and Sawers, 2007; Watanabe et al., 2012; Peters et al., 2015). It is thus no surprise that reports about the practical applications of NADP^+^-reducing H_2_ases for NADPH generation are limited. Nevertheless, the potential of these enzymes to improve the production yields of NADPH-dependent metabolites has been demonstrated. For example, two prochiral model substrates, acetophenone and (2*S*)-hydroxy-1-phenyl-propanone, were quantitatively reduced to the corresponding (*S*)-alcohol and (1*R*,2*S*)-diol by an NADPH-dependent alcohol dehydrogenase in an *in vitro* system containing H_2_ and the partially purified NADP^+^-reducing H_2_ase of *P. furiosus* (Mertens et al., 2003). Although *in vitro* examples like this demonstrate the potential of NADP^+^-H_2_ases, expression of heterologous [NiFe]-H_2_ases outside of a closely related host is still challenging (English et al., 2009). It often results in an inactive enzyme or activity that is detectable only *in vitro* (Voordouw et al., 1987; Grzeszik et al., 1997; Sun et al., 2010). Nevertheless, successful expression of heterologous [NiFe]-H_2_ase has been reported (Wells et al., 2011).

### NAD^+^ kinase and NADH kinase

NAD^+^ kinase (NADK, EC:2.7.1.23) catalyzes the conversion of NAD^+^ to NADP^+^. It is the sole enzyme leading to *de novo* NADP^+^ biosynthesis (Figures 1, 6). As such, NADK is essential for NADP^+^ and NADPH availability. Together with NADP^+^ phosphatase (NADPase), which is thought to catalyze the opposite reaction (the conversion of NADP^+^ to NAD^+^), NADK is crucial for the regulation of the intracellular balance of NAD(H) and NADP(H) (Kawai and Murata, 2008; Shi et al., 2009). Although NADPase activity has been detected in various organisms (Reidl et al., 2000; Kawai et al., 2004), to our knowledge, no enzyme that functions solely as an NADPase has been isolated to date. However, some enzymes that exhibit NADPase activity in addition to another activity have been isolated, such as the bifunctional NADP^+^ phosphatase/NAD^+^ kinase, discovered in the archaeon *Methanococcus jannaschii* (Kawai et al., 2005; Fukuda et al., 2007).

NADK activity was enriched in extracts from the yeast *Saccharomyces cerevisiae* for the first time in 1950 (Kornberg, 1950) and has since been purified from various organisms (Mcguinness and Butler, 1985; Magni et al., 1999). However, it was not until 2000 that the gene encoding NADK was identified by Kawai et al. in *Micrococcus flavus* and *Mycobacterium tuberculosis* H37Rv (Kawai et al., 2000). Since then, NADKs from various organisms have been studied in detail, and although the exact physiological functions, related pathways, and regulatory mechanisms have not always clearly been elucidated, some reviews of NADK structure, function, and potential applications are available (Magni et al., 2006; Pollak et al., 2007; Kawai and Murata, 2008; Shi et al., 2009; Agledal et al., 2010).

NADKs are ubiquitous enzymes (Table 1). Homologs, according to literature, can be found in almost all eukaryotes and prokaryotes, with the exception of the intracellular parasite *Chlamydia trachomatis* (Grose et al., 2006; Kawai and Murata, 2008; Szaszák et al., 2011). The discrepancy between the distribution shown in Table 1 and that described in the literature might reflect the rapidly increasing availability of prokaryotic genomes and errors inherent to automated genome annotation (Bakke et al., 2009). Functionally and structurally, NADKs are weakly related to other kinases, suggesting that they belong to a kinase superfamily (Labesse et al., 2002). With the exception of the NADKs of gram-negative bacteria, the enzymes accept NAD^+^ and its reduced form, NADH (Kawai and Murata, 2008). Nevertheless, they are generally referred to as NAD^+^ kinases (EC:2.7.1.23) because they usually have a strong preference for the oxidized form. NADKs with a preference for the reduced form exist as well (EC:2.7.1.86) (Outten and Culotta, 2003; Strand et al., 2003; Turner et al., 2005), but have not to our knowledge been found in prokaryotes.

Aside from NAD(H), NADKs also need a phosphoryl donor for catalysis. The preferred phosphoryl donor often is a nucleoside triphosphate, particularly ATP, but many bacterial and archaeal NADKs can also utilize the ancient inorganic energy carrier poly(P) (Kawai et al., 2000, 2005; Garavaglia et al., 2003; Sakuraba et al., 2005). In addition, some bacterial enzymes can utilize ADP or glucose-6-phosphate (Bark et al., 1993; Lindner et al., 2010). However, the use of glucose-6-phosphate as a phosphoryl donor has not been investigated further. Finally, NADKs often also require divalent cations such as Mg^2+^, Mn^2+^, Zn^2+^, or Ca^2+^ for their activity (Kawai et al., 2000, 2001; Garavaglia et al., 2003; Sakuraba et al., 2005). The NADK of *Corynebacterium glutamicum*, however, does not require divalent cations when using ATP as phosphoryl donor, but the enzyme is stimulated in their presence. When using poly(P) as phosphoryl donor, divalent cations are required for activity (Lindner et al., 2010).

According to the phosphoryl donor specificity, NADK orthologs are subdivided into several types. The classification typically applied divides the NADK orthologs into: (I) poly(P)/ATP NADKs, which can use poly(P) and ATP as phosphoryl donors and (II) ATP NADKs, which are specific for ATP (Mori et al., 2005; Nakamichi et al., 2013). Poly(P)/ATP NADKs have been identified in gram-positive bacteria such as *Mycobacterium tuberculosis, Micrococcus flatus*, and *Bacillus subtilis* (Kawai et al., 2000; Garavaglia et al., 2003) and in archaea such as *Methanococcus jannaschii* and *Pyrococcus horikoshii* (Kawai et al., 2005; Sakuraba et al., 2005). ATP NADKs have been found in gram-negative α- and γ-Proteobacteria such as *Sphingomonas* sp. A1, *Salmonella enterica*, and *E. coli* and in eukaryotes (Kawai et al., 2001; Ochiai et al., 2004; Grose et al., 2006; Nakamichi et al., 2013). A slightly different classification system is sometimes applied, dividing the NADK orthologs into: (I) ATP NADKs, which are strictly dependent on ATP, (II) NTP NADKs, which use ATP in addition to other nucleoside triphosphates, and (III) poly(P)/NTP NADKs, which use nucleoside triphosphates and poly(P) (Shi et al., 2009). The existence of these slightly different classification systems can make it difficult to ascertain the type of NADK being discussed. However, because no strict ATP NADKs (as defined in the latter system) have been identified in prokaryotes to date, it is not an issue in this review. The reaction catalyzed by NADKs can be described as either:

(13) $$\begin{array}{r} \text{NA}{\text{D}}^{+}\;+\text{ nucleoside triphosphate }\left(\text{NTP},\text{ preferably ATP}\right)\to \\ \text{ NAD}{\text{P}}^{+}\;+\text{ NDP} \end{array}$$

or

(14) $$\begin{array}{r} \text{NA}{\text{D}}^{+}\;+\text{ NTP}∕\text{poly}\left(\text{P}\right)\to \text{NAD}{\text{P}}^{+}\;+\text{ NDP}∕\text{poly}{\left(\text{P}\right)}_{\text{n}-1} \end{array}$$

NADK is the sole enzyme leading to *de novo* NADP^+^ biosynthesis. It has a central role in the regulation of the NAD(H)/NADP(H) balance because it can directly phosphorylate NADH to form NADPH or cooperate with various NADP^+^ reducing enzymes to form NAD(H)/NADP(H) metabolic networks that ultimately convert NADH into NADPH (Singh et al., 2007, 2008; Shi et al., 2009). In view of the importance of NADK, it is not surprising that disruption of NADK is lethal in various prokaryotes (Gerdes et al., 2002; Thanassi et al., 2002; Kobayashi et al., 2003; Sassetti et al., 2003; Zalacain et al., 2003; Grose et al., 2006; Poncet-Montange et al., 2007).

Because of its essential role in NADP^+^ biosynthesis and the regulation of the NAD(H)/NADP(H) balance, NADKs have attracted attention as a potential strategy by which to improve the biosynthesis of industrially valuable metabolites. Several studies have shown that overexpression of NADK-encoding genes can increase the production yields of various NADPH-dependent biosynthetic pathways, such as poly(3-hydroxybutyrate) production and thymidine production in *E. coli* (Lee et al., 2009, 2010; Li et al., 2009), lysine and isoleucine production in *C. glutamicum* (Lindner et al., 2010; Shi et al., 2012), and arginine production in *Corynebacterium crenatum* (Rahman et al., 2012). These results suggest that increased NADP^+^ availability, resulting from NADK overexpression, disturbs the intracellular redox balance, subsequently affecting the expression of NADP^+^-reducing enzymes (Lee et al., 2009; Li et al., 2009; Lee et al., 2010). However, a comparable attempt to improve isobutanol production in *E. coli* showed that NADK overexpression alone is not always sufficient and that the co-(over)expression of an NADP^+^ reducing enzyme might be required to increase NADPH availability (Shi et al., 2013). Similarly, combined overexpression of the NADK gene from *E. coli* and the NADP^+^-GAPDH gene from *Bacillus subtilis* in *E. coli* increased 2-chloropropionic acid production yield (Wang et al., 2013c). In addition to increasing NADPH availability indirectly by overexpressing NADK, a more direct approach has also been applied. For example, the production yields of GDP-L-fucose and ε-caprolactone in *E. coli* were increased by introducing the NADH kinase (EC:2.7.1.86) gene from the yeast *S. cerevisiae* (Lee et al., 2013a).

## Final remarks and conclusion

NADPH is an essential reducing agent in all organisms. It plays an important role in various biological processes and is the driving force behind numerous biosynthetic enzymatic reactions of importance to industry. However, efficient NADPH regeneration is often a bottleneck that limits the productivity of such biotransformation processes. A thorough understanding of the reactions involved in the generation of NADPH is thus essential to increase the availability of NADPH and enhance productivity in engineered production strains.

This review has provided an overview of the major canonical NADPH-generating reactions as well as recently described NADPH-generating reactions (Table 1), and it has summarized how these different reactions have been applied to improve NADPH availability. However, additional NADPH-generating enzymes, such as gluconate 5-dehydrogenase or the recently discovered NADP^+^-specific electron-bifurcating [FeFe]-hydrogenase might also contribute to the overall NADPH supply (Klasen et al., 1995; Wang et al., 2013b). Similarly, enzymes generally considered to consume NADPH or enzymes newly developed to regenerate NADPH, such as glutamate dehydrogenase and F420-dependent NADP^+^ reductases or phosphite dehydrogenase, respectively, can occasionally become important NADPH suppliers (Berk and Thauer, 1997; Lee et al., 2013b; Yokooji et al., 2013).

Although various NADPH-generating enzymes have been overexpressed successfully, no general strategy that guarantees improved NADPH supply exists. Cells usually contain various integrated, network-wide, redox balancing systems, such that engineering of NADPH-generating pathways can result in unexpected metabolic perturbations. Thus, strategies often need to be optimized individually, taking the culture conditions and genetic background of the strains into consideration. Future research involving a systems biology approach is thus required to improve our understanding of the individual NADPH-generating enzymes and their effect on various metabolic backgrounds, in order to predict which approaches will increase NADPH availability in various biotransformation processes.

### Conflict of interest statement

The authors declare that the research was conducted in the absence of any commercial or financial relationships that could be construed as a potential conflict of interest.

## References

[B1] Adachi O. Ameyama M. (1981). d-Glucose dehydrogenase from *Gluconobacter suboxydans*: solubilization, purification and characterization. Agric. Biol. Chem. 45, 159–163.

[B2] Agledal L. Niere M. Ziegler M. (2010). The phosphate makes a difference: cellular functions of NADP. Redox Rep. 15, 2–10. 10.1179/174329210X12650506623122 20196923 PMC7067316

[B3] Ahmed H. Ettema T. J. G. Tjaden B. Geerling A. C. M. van der Oost J. Siebers B. (2005). The semi-phosphorylative Entner-Doudoroff pathway in hyperthermophilic archaea: a re-evaluation. Biochem. J. 390, 529–540. 10.1042/BJ20041711 15869466 PMC1198933

[B4] Aliverti A. Faber R. Finnerty C. M. Ferioli C. Pandini V. Negri A.. (2001). Biochemical and crystallographic characterization of ferredoxin-NADP(+) reductase from nonphotosynthetic tissues. Biochemistry 40, 14501–14508. 10.1021/bi011224c 11724563

[B5] Aliverti A. Pandini V. Pennati A. de Rosa M. Zanetti G. (2008). Structural and functional diversity of ferredoxin-NADP+ reductases. Arch. Biochem. Biophys. 474, 283–291. 10.1016/j.abb.2008.02.014 18307973

[B6] Amador-Noguez D. Brasg I. A. Feng X. J. Roquet N. Rabinowitz J. D. (2011). Metabolome remodeling during the acidogenic-solventogenic transition in *Clostridium acetobutylicum*. Appl. Environ. Microbiol. 77, 7984–7997. 10.1128/AEM.05374-11 21948824 PMC3209008

[B7] Andersen K. B. von Meyenburg K. (1977). Charges of nicotinamide adenine nucleotides and adenylate energy charge as regulatory parameters of the metabolism in *Escherichia coli*. J. Biol. Chem. 252, 4151–4156. 16925

[B8] Angermayr S. A. Paszota M. Hellingwerf K. J. (2012). Engineering a cyanobacterial cell factory for production of lactic acid. Appl. Environ. Microbiol. 78, 7098–7106. 10.1128/AEM.01587-12 22865063 PMC3457509

[B9] Aoshima M. Igarashi Y. (2008). Nondecarboxylating and decarboxylating isocitrate dehydrogenases: oxalosuccinate reductase as an ancestral form of isocitrate dehydrogenase. J. Bacteriol. 190, 2050–2055. 10.1128/JB.01799-07 18203822 PMC2258884

[B10] Aoshima M. Ishii M. Igarashi Y. (2004). A novel biotin protein required for reductive carboxylation of 2-oxoglutarate by isocitrate dehydrogenase in *Hydrogenobacter thermophilus* TK-6. Mol. Microbiol. 51, 791–798. 10.1046/j.1365-2958.2003.03863.x 14731279

[B11] Arakaki A. K. Ceccarelli E. A. Carrillo N. (1997). Plant-type ferredoxin-NADP+ reductases: a basal structural framework and a multiplicity of functions. FASEB J. 11, 133–140. 9039955 10.1096/fasebj.11.2.9039955

[B12] Arnér E. S. J. Holmgren A. (2000). Physiological functions of thioredoxin and thioredoxin reductase. Eur. J. Biochem. 267, 6102–6109. 10.1046/j.1432-1327.2000.01701.x 11012661

[B13] Arutyunov D. Schmalhausen E. Orlov V. Rahuel-Clermont S. Nagradova N. Branlant G.. (2013). An unusual effect of NADP+ on the thermostability of the nonphosphorylating glyceraldehyde-3-phosphate dehydrogenase from *Streptococcus mutans*. Biochem. Cell Biol. 91, 295–302. 10.1139/bcb-2012-0104 24032678

[B14] Asadollahi M. A. Maury J. Patil K. R. Schalk M. Clark A. Nielsen J. (2009). Enhancing sesquiterpene production in *Saccharomyces cerevisiae* through *in silico* driven metabolic engineering. Metab. Eng. 11, 328–334. 10.1016/j.ymben.2009.07.001 19619667

[B15] Bakke P. Carney N. Deloache W. Gearing M. Ingvorsen K. Lotz M.. (2009). Evaluation of three automated genome annotations for *Halorhabdus utahensis*. PLoS ONE 4:e6291. 10.1371/journal.pone.0006291 19617911 PMC2707008

[B16] Balconi E. Pennati A. Crobu D. Pandini V. Cerutti R. Zanetti G.. (2009). The ferredoxin-NADP+ reductase/ferredoxin electron transfer system of *Plasmodium falciparum*. FEBS J. 276, 3825–3836. 10.1111/j.1742-4658.2009.07100.x 19523113

[B17] Banerjee S. Nandyala A. Podili R. Katoch V. Hasnain S. (2005). Comparison of *Mycobacterium tuberculosis* isocitrate dehydrogenases (ICD-1 and ICD-2) reveals differences in coenzyme affinity, oligomeric state, pH tolerance and phylogenetic affiliation. BMC Biochem. 6, 1–14. 10.1186/1471-2091-6-20 16194279 PMC1260013

[B18] Bark K. Kampfer P. Sponner A. Dott W. (1993). Polyphosphate-dependent enzymes in some coryneform bacteria isolated from sewage sludge. FEMS Microbiol. Lett. 107, 133–138. 10.1111/j.1574-6968.1993.tb06019.x 8386121

[B19] Bartek T. Blombach B. Lang S. Eikmanns B. J. Wiechert W. Oldiges M.. (2011). Comparative 13C metabolic flux analysis of pyruvate dehydrogenase complex-deficient, L-valine-producing *Corynebacterium glutamicum*. Appl. Environ. Microbiol. 77, 6644–6652. 10.1128/AEM.00575-11 21784914 PMC3187166

[B20] Bastian S. Liu X. Meyerowitz J. T. Snow C. D. Chen M. M. Y. Arnold F. H. (2011). Engineered ketol-acid reductoisomerase and alcohol dehydrogenase enable anaerobic 2-methylpropan-1-ol production at theoretical yield in *Escherichia coli*. Metab. Eng. 13, 345–352. 10.1016/j.ymben.2011.02.004 21515217

[B21] Becker J. Klopprogge C. Herold A. Zelder O. Bolten C. J. Wittmann C. (2007). Metabolic flux engineering of l-lysine production in *Corynebacterium glutamicum*—over expression and modification of G6P dehydrogenase. J. Biotechnol. 132, 99–109. 10.1016/j.jbiotec.2007.05.026 17624457

[B22] Becker J. Klopprogge C. Schroder H. Wittmann C. (2009). Metabolic engineering of the tricarboxylic acid cycle for improved lysine production by *Corynebacterium glutamicum*. Appl. Environ. Microbiol. 75, 7866–7869. 10.1128/AEM.01942-09 19820141 PMC2794105

[B23] Becker J. Klopprogge C. Zelder O. Heinzle E. Wittmann C. (2005). Amplified expression of fructose 1,6-Bisphosphatase in *Corynebacterium glutamicum* increases *in vivo* flux through the pentose phosphate pathway and lysine production on different carbon sources. Appl. Environ. Microbiol. 71, 8587–8596. 10.1128/AEM.71.12.8587-8596.2005 16332851 PMC1317465

[B24] Begley T. P. Kinsland C. Mehl R. A. Osterman A. Dorrestein P. Gerald Litwack T. B. (2001). The biosynthesis of nicotinamide adenine dinucleotides in bacteria. Vitam. Horm. 61, 103–119. 10.1016/s0083-6729(01)61003-3 11153263

[B25] Beh M. Strauss G. Huber R. Stetter K.-O. Fuchs G. (1993). Enzymes of the reductive citric acid cycle in the autotrophic eubacterium *Aquifex pyrophilus* and in the archaebacterium *Thermoproteus neutrophilus*. Arch. Microbiol. 160, 306–311. 10.1007/BF00292082

[B26] Bennett B. D. Kimball E. H. Gao M. Osterhout R. Van Dien S. J. Rabinowitz J. D. (2009). Absolute metabolite concentrations and implied enzyme active site occupancy in *Escherichia coli*. Nat. Chem. Biol. 5, 593–599. 10.1038/nchembio.186 19561621 PMC2754216

[B27] Berk H. Thauer R. K. (1997). Function of coenzyme F420-dependent NADP reductase in methanogenic archaea containing an NADP-dependent alcohol dehydrogenase. Arch. Microbiol. 168, 396–402. 10.1007/s002030050514 9325428

[B28] Bi J. Wang H. Xie J. (2011). Comparative genomics of NAD(P) biosynthesis and novel antibiotic drug targets. J. Cell. Physiol. 226, 331–340. 10.1002/jcp.22419 20857400

[B29] Bizouarn T. Althage M. Pedersen A. Tigerström A. Karlsson J. Johansson C. (2002). The organization of the membrane domain and its interaction with the NADP(H)-binding site in proton-translocating transhydrogenase from *E. coli*. Biochim. Biophys. Acta Bioenerg. 1555, 122–127. 10.1016/S0005-2728(02)00266-9 12206903

[B30] Bizouarn T. Meuller J. Axelsson M. Rydström J. (2000). The transmembrane domain and the proton channel in proton-pumping transhydrogenases. Biochim. Biophys. Acta Bioenerg. 1459, 284–290. 10.1016/S0005-2728(00)00163-8 11004441

[B31] Bock A. King P. W. Blokesch M. Posewitz M. C. (2006). Maturation of hydrogenases. Adv. Microb. Physiol. 51, 1–71. 10.1016/S0065-2911(06)51001-X 17091562

[B32] Bologna F. P. Andreo C. S. Drincovich M. F. (2007). *Escherichia coli* malic enzymes: two isoforms with substantial differences in kinetic properties, metabolic regulation, and structure. J. Bacteriol. 189, 5937–5946. 10.1128/JB.00428-07 17557829 PMC1952036

[B33] Bommareddy R. R. Chen Z. Rappert S. Zeng A.-P. (2014). A de novo NADPH generation pathway for improving lysine production of *Corynebacterium glutamicum* by rational design of the coenzyme specificity of glyceraldehyde 3-phosphate dehydrogenase. Metab. Eng. 25, 30–37. 10.1016/j.ymben.2014.06.005 24953302

[B34] Boonstra B. French C. E. Wainwright I. Bruce N. C. (1999). The udhA gene of *Escherichia coli* encodes a soluble pyridine nucleotide transhydrogenase. J. Bacteriol. 181, 1030–1034. 9922271 10.1128/jb.181.3.1030-1034.1999 PMC93474

[B35] Boonstra B. Rathbone D. A. French C. E. Walker E. H. Bruce N. C. (2000). Cofactor regeneration by a soluble pyridine nucleotide transhydrogenase for biological production of hydromorphone. Appl. Environ. Microbiol. 66, 5161–5166. 10.1128/AEM.66.12.5161-5166.2000 11097884 PMC92438

[B36] Boyd D. A. Cvitkovitch D. G. Hamilton I. R. (1995). Sequence, expression, and function of the gene for the nonphosphorylating, NADP-dependent glyceraldehyde-3-phosphate dehydrogenase of *Streptococcus mutans*. J. Bacteriol. 177, 2622–2627. 7751269 10.1128/jb.177.10.2622-2627.1995 PMC176930

[B37] Bragg P. D. Davies P. L. Hou C. (1972). Function of energy-dependent transhydrogenase in *Escherichia coli*. Biochem. Biophys. Res. Commun. 47, 1248–1255. 10.1016/0006-291X(72)90969-2 4337747

[B38] Brasen C. Esser D. Rauch B. Siebers B. (2014). Carbohydrate metabolism in Archaea: current insights into unusual enzymes and pathways and their regulation. Microbiol. Mol. Biol. Rev. 78, 89–175. 10.1128/MMBR.00041-13 24600042 PMC3957730

[B39] Brown D. I. Griendling K. K. (2009). Nox proteins in signal transduction. Free Radic. Biol. Med. 47, 1239–1253. 10.1016/j.freeradbiomed.2009.07.023 19628035 PMC2763943

[B40] Brunner N. A. Brinkmann H. Siebers B. Hensel R. (1998). NAD+-dependent glyceraldehyde-3-phosphate dehydrogenase from *Thermoproteus tenax*. The first identified archaeal member of the aldehyde dehydrogenase superfamily is a glycolytic enzyme with unusual regulatory properties. J. Biol. Chem. 273, 6149–6156. 10.1074/jbc.273.11.6149 9497334

[B41] Brunner N. A. Hensel R. (2001). Nonphosphorylating glyceraldehyde-3-phosphate dehydrogenase from *Thermoproteus tenax*. Methods Enzymol. 331, 117–131. 10.1016/S0076-6879(01)31051-0 11265454

[B42] Brunner N. A. Siebers B. Hensel R. (2001). Role of two different glyceraldehyde-3-phosphate dehydrogenases in controlling the reversible Embden-Meyerhof-Parnas pathway in *Thermoproteus tenax*: regulation on protein and transcript level. Extremophiles 5, 101–109. 10.1007/s007920100181 11354453

[B43] Buckel W. Thauer R. K. (2013). Energy conservation via electron bifurcating ferredoxin reduction and proton/Na(+) translocating ferredoxin oxidation. Biochim. Biophys. Acta 1827, 94–113. 10.1016/j.bbabio.2012.07.002 22800682

[B44] Butler J. R. Mcguinness E. T. (1982). *Candida utilis* NAD+ kinase: purification, properties and affinity gel studies. Int. J. Biochem. 14, 839–844. 10.1016/0020-711X(82)90106-9 6290285

[B45] Butler M. J. Bruheim P. Jovetic S. Marinelli F. Postma P. W. Bibb M. J. (2002). Engineering of primary carbon metabolism for improved antibiotic production in *Streptomyces lividans*. Appl. Environ. Microbiol. 68, 4731–4739. 10.1128/AEM.68.10.4731-4739.2002 12324314 PMC126421

[B46] Bylund J. Brown K. L. Movitz C. Dahlgren C. Karlsson A. (2010). Intracellular generation of superoxide by the phagocyte NADPH oxidase: how, where, and what for? Free Radic. Biol. Med. 49, 1834–1845. 10.1016/j.freeradbiomed.2010.09.016 20870019

[B47] Canonaco F. Hess T. A. Heri S. Wang T. Szyperski T. Sauer U. (2001). Metabolic flux response to phosphoglucose isomerase knock-out in *Escherichia coli* and impact of overexpression of the soluble transhydrogenase UdhA. FEMS Microbiol. Lett. 204, 247–252. 10.1111/j.1574-6968.2001.tb10892.x 11731130

[B48] Cao Z. Song P. Xu Q. Su R. Zhu G. (2011). Overexpression and biochemical characterization of soluble pyridine nucleotide transhydrogenase from *Escherichia coli*. FEMS Microbiol. Lett. 320, 9–14. 10.1111/j.1574-6968.2011.02287.x 21545646

[B49] Carrillo N. Ceccarelli E. A. (2003). Open questions in ferredoxin-NADP+ reductase catalytic mechanism. Eur. J. Biochem. 270, 1900–1915. 10.1046/j.1432-1033.2003.03566.x 12709048

[B50] Casalot L. De Luca G. Dermoun Z. Rousset M. de Philip P. (2002). Evidence for a fourth hydrogenase in *Desulfovibrio fructosovorans*. J. Bacteriol. 184, 853–856. 10.1128/JB.184.3.853-856.2002 11790758 PMC139505

[B51] Ceccarelli E. A. Arakaki A. K. Cortez N. Carrillo N. (2004). Functional plasticity and catalytic efficiency in plant and bacterial ferredoxin-NADP(H) reductases. Biochim. Biophys. Acta 1698, 155–165. 10.1016/j.bbapap.2003.12.005 15134648

[B52] Celton M. Goelzer A. Camarasa C. Fromion V. Dequin S. (2012). A constraint-based model analysis of the metabolic consequences of increased NADPH oxidation in *Saccharomyces cerevisiae*. Metab. Eng. 14, 366–379. 10.1016/j.ymben.2012.03.008 22709677

[B53] Centeno-Leija S. Huerta-Beristain G. Giles-Gomez M. Bolivar F. Gosset G. Martinez A. (2014). Improving poly-3-hydroxybutyrate production in *Escherichia coli* by combining the increase in the NADPH pool and acetyl-CoA availability. Antonie Van Leeuwenhoek 105, 687–696. 10.1007/s10482-014-0124-5 24500003

[B54] Centeno-Leija S. Utrilla J. Flores N. Rodriguez A. Gosset G. Martinez A. (2013). Metabolic and transcriptional response of *Escherichia coli* with a NADP(+)-dependent glyceraldehyde 3-phosphate dehydrogenase from *Streptococcus mutans*. Antonie Van Leeuwenhoek 104, 913–924. 10.1007/s10482-013-0010-6 23989925

[B55] Chang D. Y. Hou C. Bragg P. D. (1992). Anomalous effect of uncouplers on respiratory chain-linked transhydrogenation in *Escherichia coli* membranes: evidence for a localized proton pathway? Arch. Biochem. Biophys. 293, 246–253. 10.1016/0003-9861(92)90392-A 1311161

[B56] Chang G.-G. Tong L. (2003). Structure and function of malic enzymes, a new class of oxidative decarboxylases. Biochemistry 42, 12721–12733. 10.1021/bi035251+ 14596586

[B57] Chemler J. A. Fowler Z. L. Mchugh K. P. Koffas M. A. G. (2010). Improving NADPH availability for natural product biosynthesis in *Escherichia coli* by metabolic engineering. Metab. Eng. 12, 96–104. 10.1016/j.ymben.2009.07.003 19628048

[B58] Chen R. Jeong S. S. (2000). Functional prediction: identification of protein orthologs and paralogs. Protein Sci. 9, 2344–2353. 10.1110/ps.9.12.2344 11206056 PMC2144510

[B59] Chenault H. K. Simon E. S. Whitesides G. M. (1988). Cofactor regeneration for enzyme-catalysed synthesis. Biotechnol. Genet. Eng. Rev. 6, 221–270. 10.1080/02648725.1988.10647849 3071373

[B60] Chenault H. K. Whitesides G. M. (1987). Regeneration of nicotinamide cofactors for use in organic synthesis. Appl. Biochem. Biotechnol. 14, 147–197. 10.1007/BF02798431 3304160

[B61] Chin J. W. Cirino P. C. (2011). Improved NADPH supply for xylitol production by engineered *Escherichia Coli* with glycolytic mutations. Biotechnol. Prog. 27, 333–341. 10.1002/btpr.559 21344680

[B62] Chin J. W. Khankal R. Monroe C. A. Maranas C. D. Cirino P. C. (2009). Analysis of NADPH supply during xylitol production by engineered *Escherichia coli*. Biotechnol. Bioeng. 102, 209–220. 10.1002/bit.22060 18698648

[B63] Chini E. N. De Toledo F. G. S. (2002). Nicotinic acid adenine dinucleotide phosphate: a new intracellular second messenger? Am. J. Physiol. Cell Physiol. 282, C1191–C1198. 10.1152/ajpcell.00475.2001 11997232

[B64] Choi J.-C. Shin H.-D. Lee Y.-H. (2003). Modulation of 3-hydroxyvalerate molar fraction in poly(3-hydroxybutyrate-3-hydroxyvalerate) using *Ralstonia eutropha* transformant co-amplifying phbC and NADPH generation-related zwf genes. Enzyme Microb. Technol. 32, 178–185. 10.1016/S0141-0229(02)00274-0

[B65] Christiansen T. Christensen B. Nielsen J. (2002). Metabolic network analysis of *Bacillus clausii* on minimal and semirich medium using (13)C-labeled glucose. Metab. Eng. 4, 159–169. 10.1006/mben.2001.0219 12009795

[B66] Churamani D. Carrey E. A. Dickinson G. D. Patel S. (2004). Determination of cellular nicotinic acid-adenine dinucleotide phosphate (NAADP) levels. Biochem. J. 380, 449–454. 10.1042/BJ20031754 14984366 PMC1224178

[B67] Cohen P. T. Kaplan N. O. (1970). Purification and properties of the pyridine nucleotide transhydrogenase from *Pseudomonas aeruginosa*. J. Biol. Chem. 245, 2825–2836. 4393131

[B68] Conway T. (1992). The entner-doudoroff pathway: history, physiology and molecular biology. FEMS Microbiol. Rev. 9, 1–27. 10.1111/j.1574-6968.1992.tb05822.x 1389313

[B69] Cotton N. P. Lever T. M. Nore B. F. Jones M. R. Jackson J. B. (1989). The coupling between protonmotive force and the NAD(P)+ transhydrogenase in chromatophores from photosynthetic bacteria. Eur. J. Biochem. 182, 593–603. 10.1111/j.1432-1033.1989.tb14868.x 2546762

[B70] Cozzone A. J. (1998). Regulation of acetate metabolism by protein phosphorylation in enteric bacteria. Annu. Rev. Microbiol. 52, 127–164. 10.1146/annurev.micro.52.1.127 9891796

[B71] Crow V. L. Wittenberger C. L. (1979). Separation and properties of NAD+- and NADP+-dependent glyceraldehyde-3-phosphate dehydrogenases from *Streptococcus mutans*. J. Biol. Chem. 254, 1134–1142. 33184

[B72] Dauner M. Storni T. Sauer U. (2001). *Bacillus subtilis* metabolism and energetics in carbon-limited and excess-carbon chemostat culture. J. Bacteriol. 183, 7308–7317. 10.1128/JB.183.24.7308-7317.2001 11717290 PMC95580

[B73] Davis W. B. (1980). Identification of a nicotinamide adenine dinucleotide glycohydrolase and an associated inhibitor in isoniazid-susceptible and -resistant *Mycobacterium phlei*. Antimicrob. Agents Chemother. 17, 663–668. 10.1128/AAC.17.4.663 6249194 PMC283849

[B74] Decorosi F. Lori L. Santopolo L. Tatti E. Giovannetti L. Viti C. (2011). Characterization of a Cr(VI)-sensitive *Pseudomonas corrugata* 28 mutant impaired in a pyridine nucleotide transhydrogenase gene. Res. Microbiol. 162, 747–755. 10.1016/j.resmic.2011.06.014 21807093

[B75] Driscoll B. T. Finan T. M. (1997). Properties of NAD(+)- and NADP(+)-dependent malic enzymes of *Rhizobium (Sinorhizobium) meliloti* and differential expression of their genes in nitrogen-fixing bacteroids. Microbiology 143(Pt 2), 489–498. 10.1099/00221287-143-2-489 9043124

[B76] Eguchi T. Kuge Y. Inoue K. Yoshikawa N. Mochida K. Uwajima T. (1992). NADPH regeneration by glucose dehydrogenase from *Gluconobacter scleroides* for l-leucovorin synthesis. Biosci. Biotechnol. Biochem. 56, 701–703. 10.1271/bbb.56.701 1368340

[B77] Eikmanns B. J. Rittmann D. Sahm H. (1995). Cloning, sequence analysis, expression, and inactivation of the *Corynebacterium glutamicum* icd gene encoding isocitrate dehydrogenase and biochemical characterization of the enzyme. J. Bacteriol. 177, 774–782. 7836312 10.1128/jb.177.3.774-782.1995 PMC176656

[B78] English C. M. Eckert C. Brown K. Seibert M. King P. W. (2009). Recombinant and *in vitro* expression systems for hydrogenases: new frontiers in basic and applied studies for biological and synthetic H2 production. Dalton Trans. 9970–9978. 10.1039/b913426n 19904422

[B79] Espariz M. Repizo G. Blancato V. Mortera P. Alarcon S. Magni C. (2011). Identification of malic and soluble oxaloacetate decarboxylase enzymes in *Enterococcus faecalis*. FEBS J. 278, 2140–2151. 10.1111/j.1742-4658.2011.08131.x 21518252

[B80] Esser D. Kouril T. Talfournier F. Polkowska J. Schrader T. Bräsen C.. (2013). Unraveling the function of paralogs of the aldehyde dehydrogenase super family from *Sulfolobus solfataricus*. Extremophiles 17, 205–216. 10.1007/s00792-012-0507-3 23296511

[B81] Ettema T. J. G. Ahmed H. Geerling A. C. M. Van Der Oost J. Siebers B. (2008). The non-phosphorylating glyceraldehyde-3-phosphate dehydrogenase (GAPN) of *Sulfolobus solfataricus*: a key-enzyme of the semi-phosphorylative branch of the Entner-Doudoroff pathway. Extremophiles 12, 75–88. 10.1007/s00792-007-0082-1 17549431

[B82] Everse J. Griffin J. B. Kaplan N. O. (1975). The pyridine nucleosidase from *Bacillus subtilis*. Kinetic properties and enzyme-inhibitor interactions. Arch. Biochem. Biophys. 169, 714–723. 10.1016/0003-9861(75)90216-7 241299

[B83] Ewen K. M. Kleser M. Bernhardt R. (2011). Adrenodoxin: the archetype of vertebrate-type [2Fe–2S] cluster ferredoxins. Biochim. Biophys. Acta Proteins Proteomics 1814, 111–125. 10.1016/j.bbapap.2010.06.003 20538075

[B84] Fabris M. Matthijs M. Rombauts S. Vyverman W. Goossens A. Baart G. J. E. (2012). The metabolic blueprint of *Phaeodactylum tricornutum* reveals a eukaryotic Entner-Doudoroff glycolytic pathway. Plant J. 70, 1004–1014. 10.1111/j.1365-313X.2012.04941.x 22332784

[B85] Ferri S. Kojima K. Sode K. (2011). Review of glucose oxidases and glucose dehydrogenases: a bird's eye view of glucose sensing enzymes. J. Diabetes Sci. Technol. 5, 1068–1076. 10.1177/193229681100500507 22027299 PMC3208862

[B86] Fillinger S. Boschi-Muller S. Azza S. D. Dervyn E. Branlant G. Aymerich S. (2000). Two Glyceraldehyde-3-phosphate dehydrogenases with opposite physiological roles in a nonphotosynthetic bacterium. J. Biol. Chem. 275, 14031–14037. 10.1074/jbc.275.19.14031 10799476

[B87] Fischer E. Sauer U. (2003). A novel metabolic cycle catalyzes glucose oxidation and anaplerosis in hungry *Escherichia coli*. J. Biol. Chem. 278, 46446–46451. 10.1074/jbc.M307968200 12963713

[B88] Flamholz A. Noor E. Bar-Even A. Milo R. (2012). eQuilibrator—the biochemical thermodynamics calculator. Nucleic Acids Res. 40, D770–D775. 10.1093/nar/gkr874 22064852 PMC3245061

[B89] Foreman J. Demidchik V. Bothwell J. H. F. Mylona P. Miedema H. Torres M. A.. (2003). Reactive oxygen species produced by NADPH oxidase regulate plant cell growth. Nature 422, 442–446. 10.1038/nature01485 12660786

[B90] Forzi L. Sawers R. G. (2007). Maturation of [NiFe]-hydrogenases in *Escherichia coli*. Biometals 20, 565–578. 10.1007/s10534-006-9048-5 17216401

[B91] French C. E. Boonstra B. Bufton K. A. Bruce N. C. (1997). Cloning, sequence, and properties of the soluble pyridine nucleotide transhydrogenase of *Pseudomonas fluorescens*. J. Bacteriol. 179, 2761–2765. 9098078 10.1128/jb.179.8.2761-2765.1997 PMC179029

[B92] Frenkel R. (1975). Regulation and physiological functions of malic enzymes. Curr. Top. Cell. Regul. 9, 157–181. 10.1016/B978-0-12-152809-6.50012-3 235406

[B93] Fuhrer T. Fischer E. Sauer U. (2005). Experimental identification and quantification of glucose metabolism in seven bacterial species. J. Bacteriol. 187, 1581–1590. 10.1128/JB.187.5.1581-1590.2005 15716428 PMC1064017

[B94] Fuhrer T. Sauer U. (2009). Different biochemical mechanisms ensure network-wide balancing of reducing equivalents in microbial metabolism. J. Bacteriol. 191, 2112–2121. 10.1128/JB.01523-08 19181802 PMC2655529

[B95] Fukuda C. Kawai S. Murata K. (2007). NADP(H) Phosphatase activities of archaeal inositol monophosphatase and eubacterial 3′-Phosphoadenosine 5′-Phosphate phosphatase. Appl. Environ. Microbiol. 73, 5447–5452. 10.1128/AEM.02703-06 17616624 PMC2042097

[B96] Fukuda W. Ismail Y. S. Fukui T. Atomi H. Imanaka T. (2005). Characterization of an archaeal malic enzyme from the hyperthermophilic archaeon *Thermococcus kodakaraensis* KOD1. Archaea 1, 293–301. 10.1155/2005/250757 15876562 PMC2685551

[B97] Garavaglia S. Galizzi A. Rizzi M. (2003). Allosteric regulation of *Bacillus subtilis* NAD kinase by quinolinic acid. J. Bacteriol. 185, 4844–4850. 10.1128/JB.185.16.4844-4850.2003 12897004 PMC166466

[B98] Garrido-Pertierra A. Martinez Marcos C. Martin Fernandez M. Ruiz-Amil M. (1983). Properties and function of malate enzyme from *Pseudomonas putida*. Biochimie 65, 629–635. 10.1016/S0300-9084(84)80026-7 6673742

[B99] Gazzaniga F. Stebbins R. Chang S. Z. Mcpeek M. A. Brenner C. (2009). Microbial NAD metabolism: lessons from comparative genomics. Microbiol. Mol. Biol. Rev. 73, 529–541. 10.1128/MMBR.00042-08 19721089 PMC2738131

[B100] Gerdes S. Y. Scholle M. D. D'souza M. Bernal A. Baev M. V. Farrell M.. (2002). From genetic footprinting to antimicrobial drug targets: examples in cofactor biosynthetic pathways. J. Bacteriol. 184, 4555–4572. 10.1128/JB.184.16.4555-4572.2002 12142426 PMC135229

[B101] Germer F. Zebger I. Saggu M. Lendzian F. Schulz R. Appel J. (2009). Overexpression, isolation, and spectroscopic characterization of the bidirectional [NiFe] hydrogenase from *Synechocystis* sp. PCC 6803. J. Biol. Chem. 284, 36462–36472. 10.1074/jbc.M109.028795 19801638 PMC2794762

[B102] Ghosh J. Anderson P. J. Chandrasekaran S. Caparon M. G. (2010). Characterization of *Streptococcus pyogenes* beta-NAD+ glycohydrolase: re-evaluation of enzymatic properties associated with pathogenesis. J. Biol. Chem. 285, 5683–5694. 10.1074/jbc.M109.070300 20018886 PMC2820796

[B103] Giro M. Carrillo N. Krapp A. R. (2006). Glucose-6-phosphate dehydrogenase and ferredoxin-NADP(H) reductase contribute to damage repair during the soxRS response of *Escherichia coli*. Microbiology 152, 1119–1128. 10.1099/mic.0.28612-0 16549675

[B104] Goss T. Hanke G. (2014). The end of the line: can ferredoxin and ferredoxin NADP(H) oxidoreductase determine the fate of photosynthetic electrons? Curr. Protein Pept. Sci. 15, 385–393. 10.2174/1389203715666140327113733 24678667 PMC4030315

[B105] Gossmann T. I. Ziegler M. Puntervoll P. De Figueiredo L. F. Schuster S. Heiland I. (2012). NAD+ biosynthesis and salvage - a phylogenetic perspective. FEBS J. 279, 3355–3363. 10.1111/j.1742-4658.2012.08559.x 22404877

[B106] Gourdon P. Baucher M. F. Lindley N. D. Guyonvarch A. (2000). Cloning of the malic enzyme gene from *Corynebacterium glutamicum* and role of the enzyme in lactate metabolism. Appl. Environ. Microbiol. 66, 2981–2987. 10.1128/AEM.66.7.2981-2987.2000 10877795 PMC92100

[B107] Grose J. H. Joss L. Velick S. F. Roth J. R. (2006). Evidence that feedback inhibition of NAD kinase controls responses to oxidative stress. Proc. Natl. Acad. Sci. U.S.A. 103, 7601–7606. 10.1073/pnas.0602494103 16682646 PMC1472491

[B108] Grzeszik C. Lubbers M. Reh M. Schlegel H. G. (1997). Genes encoding the NAD-reducing hydrogenase of *Rhodococcus opacus* MR11. Microbiology 143(Pt 4), 1271–1286. 10.1099/00221287-143-4-1271 9141690

[B109] Guo Z.-P. Zhang L. Ding Z.-Y. Wang Z.-X. Shi G.-Y. (2011). Improving ethanol productivity by modification of glycolytic redox factor generation in glycerol-3-phosphate dehydrogenase mutants of an industrial ethanol yeast. J. Ind. Microbiol. Biotechnol. 38, 935–943. 10.1007/s10295-010-0864-9 20824484

[B110] Hansen E. J. Juni E. (1975). Isolation of mutants of *Escherichia coli* lacking NAD- and NADP-linked malic. Biochem. Biophys. Res. Commun. 65, 559–566. 10.1016/S0006-291X(75)80183-5 238534

[B111] Harold F. M. (1986). The Vital Force: A Study of Bioenergetics. New York, NY: W. H. Freeman.

[B112] He L. Xiao Y. Gebreselassie N. Zhang F. Antoniewicz M. R. Tang Y. J.. (2014). Central metabolic responses to the overproduction of fatty acids in *Escherichia coli* based on 13C-metabolic flux analysis. Biotechnol. Bioeng. 111, 575–585. 10.1002/bit.25124 24122357 PMC5901677

[B113] Hickman J. W. Barber R. D. Skaar E. P. Donohue T. J. (2002). Link between the membrane-bound pyridine nucleotide transhydrogenase and glutathione-dependent processes in *Rhodobacter sphaeroides*. J. Bacteriol. 184, 400–409. 10.1128/JB.184.2.400-409.2002 11751816 PMC139586

[B114] Hoek J. B. Rydstrom J. (1988). Physiological roles of nicotinamide nucleotide transhydrogenase. Biochem. J. 254, 1–10. 3052428 10.1042/bj2540001 PMC1135030

[B115] Hojeberg B. Rydstrom J. (1977). Ca2+-dependent allosteric regulation of nicotinamide nucleotide transhydrogenase from *Pseudomonas aeruginosa*. Eur. J. Biochem. 77, 235–241. 10.1111/j.1432-1033.1977.tb11662.x 19248

[B116] Hong S. H. Lee S. Y. (2001). Metabolic flux analysis for succinic acid production by recombinant *Escherichia coli* with amplified malic enzyme activity. Biotechnol. Bioeng. 74, 89–95. 10.1002/bit.1098 11369997

[B117] Hsieh J.-Y. Hung H.-C. (2009). Engineering of the cofactor specificities and isoform-specific inhibition of malic enzyme. J. Biol. Chem. 284, 4536–4544. 10.1074/jbc.M807008200 19091740

[B118] Hua Q. Yang C. Baba T. Mori H. Shimizu K. (2003). Responses of the central metabolism in *Escherichia coli* to phosphoglucose isomerase and glucose-6-phosphate dehydrogenase knockouts. J. Bacteriol. 185, 7053–7067. 10.1128/JB.185.24.7053-7067.2003 14645264 PMC296241

[B119] Huang H. Wang S. Moll J. Thauer R. K. (2012). Electron bifurcation involved in the energy metabolism of the acetogenic bacterium *Moorella thermoacetica* growing on glucose or H2 plus CO2. J. Bacteriol. 194, 3689–3699. 10.1128/JB.00385-12 22582275 PMC3393501

[B120] Huang M. Wang Y. Liu J. Mao Z. (2011). Multiple strategies for metabolic engineering of *Escherichia coli* for efficient production of coenzyme. Chin. J. Chem. Eng. 19, 316–326. 10.1016/S1004-9541(11)60171-7

[B121] Hügler M. Menendez C. Schägger H. Fuchs G. (2002). Malonyl-coenzyme a reductase from *Chloroflexus aurantiacus*, a key enzyme of the 3-Hydroxypropionate cycle for autotrophic CO2 Fixation. J. Bacteriol. 184, 2404–2410. 10.1128/JB.184.9.2404-2410.2002 11948153 PMC134993

[B122] Iddar A. Valverde F. Assobhei O. Serrano A. Soukri A. (2005). Widespread occurrence of non-phosphorylating glyceraldehyde-3-phosphate dehydrogenase among gram-positive bacteria. Int. Microbiol. 8, 251–258. 16562377

[B123] Iddar A. Valverde F. Serrano A. Soukri A. (2002). Expression, purification, and characterization of recombinant nonphosphorylating NADP-dependent glyceraldehyde-3-phosphate dehydrogenase from *Clostridium acetobutylicum*. Protein Expr. Purif. 25, 519–526. 10.1016/S1046-5928(02)00032-3 12182834

[B124] Iddar A. Valverde F. Serrano A. Soukri A. (2003). Purification of recombinant non-phosphorylating NADP-dependent glyceraldehyde-3-phosphate dehydrogenase from *Streptococcus pyogenes* expressed in *E. coli*. Mol. Cell. Biochem. 247, 195–203. 10.1023/A:1024112027440 12841648

[B125] Ikeda T. Nakamura M. Arai H. Ishii M. Igarashi Y. (2009). Ferredoxin-NADP reductase from the thermophilic hydrogen-oxidizing bacterium, *Hydrogenobacter thermophilus* TK-6. FEMS Microbiol. Lett. 297, 124–130. 10.1111/j.1574-6968.2009.01667.x 19552713

[B126] Imam S. Noguera D. R. Donohue T. J. (2013). Global insights into energetic and metabolic networks in *Rhodobacter sphaeroides*. BMC Syst. Biol. 7:89. 10.1186/1752-0509-7-89 24034347 PMC3849096

[B127] Inoue H. Tamura T. Ehara N. Nishito A. Nakayama Y. Maekawa M.. (2002). Biochemical and molecular characterization of the NAD(+)-dependent isocitrate dehydrogenase from the chemolithotroph *Acidithiobacillus thiooxidans*. FEMS Microbiol. Lett. 214, 127–132. 10.1111/j.1574-6968.2002.tb11335.x 12204383

[B128] Ito F. Chishiki H. Fushinobu S. Wakagi T. (2012). Comparative analysis of two glyceraldehyde-3-phosphate dehydrogenases from a thermoacidophilic archaeon, *Sulfolobus tokodaii*. FEBS Lett. 586, 3097–3103. 10.1016/j.febslet.2012.07.059 22841742

[B129] Iwakura M. Tokushige M. Katsuki H. Muramatsu S. (1978). Studies on regulatory functions of malic enzymes. V. Comparative studies of malic enzymes in bacteria. J. Biochem. 83, 1387–1394. 96110 10.1093/oxfordjournals.jbchem.a132048

[B130] Jackson B. Brocker C. Thompson D. C. Black W. Vasiliou K. Nebert D. W.. (2011). Update on the aldehyde dehydrogenase gene (ALDH) superfamily. Hum. Genomics 5, 283–303. 10.1186/1479-7364-5-4-283 21712190 PMC3392178

[B131] Jackson J. B. (2003). Proton translocation by transhydrogenase. FEBS Lett. 545, 18–24. 10.1016/S0014-5793(03)00388-0 12788487

[B132] Jackson J. B. (2012). A review of the binding-change mechanism for proton-translocating transhydrogenase. Biochim. Biophys. Acta 1817, 1839–1846. 10.1016/j.bbabio.2012.04.006 22538293

[B133] Jackson J. B. Obiozo U. M. (2009). Proton-translocating transhydrogenase in photosynthetic bacteria, in The Purple Phototrophic Bacteria, eds Hunter C. N. Daldal F. Thurnauer M. Beatty J. T. (Dordrecht: Springer), 495–508.

[B134] Jackson J. B. Quirk P. G. Cotton N. P. Venning J. D. Gupta S. Bizouarn T.. (1998). Interdomain hydride transfer in proton-translocating transhydrogenase. Biochim. Biophys. Acta 1365, 79–86. 10.1016/S0005-2728(98)00046-2 9693725

[B135] Jain M. K. Datta R. Zeikus J. G. (1989). High-value organic acids fermentation - emerging processes and products, in Bioprocess Engineering: The First Generation, ed Ghose T. K. (Chichester: Ellis Horwood Ltd), 366–389.

[B136] Jan J. Martinez I. Wang Y. Bennett G. N. San K.-Y. (2013). Metabolic engineering and transhydrogenase effects on NADPH availability in *Escherichia coli*. Biotechnol. Prog. 29, 1124–1130. 10.1002/btpr.1765 23794523

[B137] Jiang L.-Y. Zhang Y.-Y. Li Z. Liu J.-Z. (2013). Metabolic engineering of *Corynebacterium glutamicum* for increasing the production of L-ornithine by increasing NADPH availability. J. Ind. Microbiol. Biotechnol. 40, 1143–1151. 10.1007/s10295-013-1306-2 23836141

[B138] Jiao Z. Baba T. Mori H. Shimizu K. (2003). Analysis of metabolic and physiological responses to gnd knockout in *Escherichia coli* by using C-13 tracer experiment and enzyme activity measurement. FEMS Microbiol. Lett. 220, 295–301. 10.1016/S0378-1097(03)00133-2 12670695

[B139] Jonsbu E. Christensen B. Nielsen J. (2001). Changes of *in vivo* fluxes through central metabolic pathways during the production of nystatin by *Streptomyces noursei* in batch culture. Appl. Microbiol. Biotechnol. 56, 93–100. 10.1007/s002530100613 11499953

[B140] Kabir M. M. Shimizu K. (2003). Fermentation characteristics and protein expression patterns in a recombinant *Escherichia coli* mutant lacking phosphoglucose isomerase for poly(3-hydroxybutyrate) production. Appl. Microbiol. Biotechnol. 62, 244–255. 10.1007/s00253-003-1257-z 12883871

[B141] Kabir M. M. Shimizu K. (2004). Metabolic regulation analysis of icd-gene knockout *Escherichia coli* based on 2D electrophoresis with MALDI-TOF mass spectrometry and enzyme activity measurements. Appl. Microbiol. Biotechnol. 65, 84–96. 10.1007/s00253-004-1627-1 15221231

[B142] Kabus A. Georgi T. Wendisch V. F. Bott M. (2007). Expression of the *Escherichia coli* pntAB genes encoding a membrane-bound transhydrogenase in *Corynebacterium glutamicum* improves L-lysine formation. Appl. Microbiol. Biotechnol. 75, 47–53. 10.1007/s00253-006-0804-9 17216441

[B143] Kanai T. Ito S. Imanaka T. (2003). Characterization of a cytosolic NiFe-hydrogenase from the hyperthermophilic archaeon *Thermococcus kodakaraensis* KOD1. J. Bacteriol. 185, 1705–1711. 10.1128/JB.185.5.1705-1711.2003 12591889 PMC148058

[B144] Kanai T. Matsuoka R. Beppu H. Nakajima A. Okada Y. Atomi H.. (2011). Distinct physiological roles of the three [NiFe]-hydrogenase orthologs in the hyperthermophilic archaeon *Thermococcus kodakarensis*. J. Bacteriol. 193, 3109–3116. 10.1128/JB.01072-10 21515783 PMC3133214

[B145] Kanao T. Kawamura M. Fukui T. Atomi H. Imanaka T. (2002). Characterization of isocitrate dehydrogenase from the green sulfur bacterium *Chlorobium limicola*. A carbon dioxide-fixing enzyme in the reductive tricarboxylic acid cycle. Eur. J. Biochem./FEBS 269, 1926–1931. 10.1046/j.1432-1033.2002.02849.x 11952794

[B146] Kao K. C. Tran L. M. Liao J. C. (2005). A global regulatory role of gluconeogenic genes in *Escherichia coli* revealed by transcriptome network analysis. J. Biol. Chem. 280, 36079–36087. 10.1074/jbc.M508202200 16141204

[B147] Karplus P. A. Faber H. R. (2004). Structural aspects of plant ferredoxin: NADP(+) oxidoreductases. Photosyn. Res. 81, 303–315. 10.1023/B:PRES.0000036884.57303.2e 16034534

[B148] Kataoka M. Sri Rohani L. P. Wada M. Kita K. Yanase H. Urabe I.. (1998). *Escherichia coli* transformant expressing the glucose dehydrogenase gene from *Bacillus megaterium* as a cofactor regenerator in a chiral alcohol production system. Biosci. Biotechnol. Biochem. 62, 167–169. 10.1271/bbb.62.167 9501530

[B149] Kataoka M. Yamamoto K. Kawabata H. Wada M. Kita K. Yanase H.. (1999). Stereoselective reduction of ethyl 4-chloro-3-oxobutanoate by *Escherichia coli* transformant cells coexpressing the aldehyde reductase and glucose dehydrogenase genes. Appl. Microbiol. Biotechnol. 51, 486–490. 10.1007/s002530051421 10341431

[B150] Kawai S. Fukuda C. Mukai T. Murata K. (2005). MJ0917 in archaeon *Methanococcus jannaschii* is a novel NADP phosphatase/NAD kinase. J. Biol. Chem. 280, 39200–39207. 10.1074/jbc.M506426200 16192277

[B151] Kawai S. Mori S. Mukai T. Hashimoto W. Murata K. (2001). Molecular characterization of *Escherichia coli* NAD kinase. Eur. J. Biochem. 268, 4359–4365. 10.1046/j.1432-1327.2001.02358.x 11488932

[B152] Kawai S. Mori S. Mukai T. Murata K. (2004). Cytosolic NADP phosphatases I and II from *Arthrobacter* sp. strain KM: implication in regulation of NAD+/NADP+ balance. J. Basic Microbiol. 44, 185–196. 10.1002/jobm.200310362 15162392

[B153] Kawai S. Mori S. Mukai T. Suzuki S. Yamada T. Hashimoto W.. (2000). Inorganic Polyphosphate/ATP-NAD Kinase of *Micrococcus flavus* and *Mycobacterium tuberculosis* H37Rv. Biochem. Biophys. Res. Commun. 276, 57–63. 10.1006/bbrc.2000.3433 11006082

[B154] Kawai S. Murata K. (2008). Structure and function of NAD kinase and NADP phosphatase: key enzymes that regulate the intracellular balance of NAD(H) and NADP(H). Biosci. Biotechnol. Biochem. 72, 919–930. 10.1271/bbb.70738 18391451

[B155] Kawai S. Suzuki H. Yamamoto K. Inui M. Yukawa H. Kumagai H. (1996). Purification and characterization of a malic enzyme from the ruminal bacterium *Streptococcus bovis* ATCC 15352 and cloning and sequencing of its gene. Appl. Environ. Microbiol. 62, 2692–2700. 8702261 10.1128/aem.62.8.2692-2700.1996 PMC168054

[B156] Kelly G. J. Gibbs M. (1973). Nonreversible d-glyceraldehyde 3-phosphate dehydrogenase of plant tissues. Plant Physiol. 52, 111–118. 10.1104/pp.52.2.111 16658509 PMC366450

[B157] Kim Y. M. Cho H.-S. Jung G. Y. Park J. M. (2011). Engineering the pentose phosphate pathway to improve hydrogen yield in recombinant *Escherichia coli*. Biotechnol. Bioeng. 108, 2941–2946. 10.1002/bit.23259 21732330

[B158] Kizaki N. Yasohara Y. Hasegawa J. Wada M. Kataoka M. Shimizu S. (2001). Synthesis of optically pure ethyl (S)-4-chloro-3-hydroxybutanoate by *Escherichia coli* transformant cells coexpressing the carbonyl reductase and glucose dehydrogenase genes. Appl. Microbiol. Biotechnol. 55, 590–595. 10.1007/s002530100599 11414326

[B159] Klasen R. Bringer-Meyer S. Sahm H. (1995). Biochemical characterization and sequence analysis of the gluconate:NADP 5-oxidoreductase gene from *Gluconobacter oxydans*. J. Bacteriol. 177, 2637–2643. 7751271 10.1128/jb.177.10.2637-2643.1995 PMC176932

[B160] Kletzien R. F. Harris P. K. Foellmi L. A. (1994). Glucose-6-phosphate dehydrogenase: a “housekeeping” enzyme subject to tissue-specific regulation by hormones, nutrients, and oxidant stress. FASEB J. 8, 174–181. 8119488 10.1096/fasebj.8.2.8119488

[B161] Kobayashi K. Ehrlich S. D. Albertini A. Amati G. Andersen K. K. Arnaud M.. (2003). Essential *Bacillus subtilis* genes. Proc. Natl. Acad. Sci. U.S.A. 100, 4678–4683. 10.1073/pnas.0730515100 12682299 PMC153615

[B162] Koh H.-J. Lee S.-M. Son B.-G. Lee S.-H. Ryoo Z. Y. Chang K.-T.. (2004). Cytosolic NADP+-dependent isocitrate dehydrogenase plays a key role in lipid metabolism. J. Biol. Chem. 279, 39968–39974. 10.1074/jbc.M402260200 15254034

[B163] Koksharova O. Schubert M. Shestakov S. Cerff R. (1998). Genetic and biochemical evidence for distinct key functions of two highly divergent GAPDH genes in catabolic and anabolic carbon flow of the *cyanobacterium Synechocystis* sp. PCC 6803. Plant Mol. Biol. 36, 183–194. 10.1023/A:1005925732743 9484473

[B164] Komori H. Seo D. Sakurai T. Higuchi Y. (2010). Crystal structure analysis of *Bacillus subtilis* ferredoxin-NADP(+) oxidoreductase and the structural basis for its substrate selectivity. Protein Sci. 19, 2279–2290. 10.1002/pro.508 20878669 PMC3009396

[B165] Kornberg A. (1950). Enzymatic synthesis of triphosphopyridine nucleotide. J. Biol. Chem. 182, 805–813.

[B166] Kramer D. M. Evans J. R. (2011). The importance of energy balance in improving photosynthetic productivity. Plant Physiol. 155, 70–78. 10.1104/pp.110.166652 21078862 PMC3075755

[B167] Krapp A. R. Rodriguez R. E. Poli H. O. Paladini D. H. Palatnik J. F. Carrillo N. (2002). The flavoenzyme ferredoxin (flavodoxin)-NADP(H) reductase modulates NADP(H) homeostasis during the soxRS response of *Escherichia coli*. J. Bacteriol. 184, 1474–1480. 10.1128/JB.184.5.1474-1480.2002 11844783 PMC134851

[B168] Kuo C. C. Tsai L. C. Chin T. Y. Chang G. G. Chou W. Y. (2000). Lysine residues 162 and 340 are involved in the catalysis and coenzyme binding of NADP(+)-dependent malic enzyme from pigeon. Biochem. Biophys. Res. Commun. 270, 821–825. 10.1006/bbrc.2000.2502 10772909

[B169] Kurnasov O. Goral V. Colabroy K. Gerdes S. Anantha S. Osterman A.. (2003). NAD biosynthesis: identification of the tryptophan to quinolinate pathway in bacteria. Chem. Biol. 10, 1195–1204. 10.1016/j.chembiol.2003.11.011 14700627

[B170] Labesse G. Douguet D. Assairi L. Gilles A. M. (2002). Diacylglyceride kinases, sphingosine kinases and NAD kinases: distant relatives of 6-phosphofructokinases. Trends Biochem. Sci. 27, 273–275. 10.1016/S0968-0004(02)02093-5 12069781

[B171] Lakshmi T. M. Helling R. B. (1976). Selection for citrate synthase deficiency in icd mutants of *Escherichia coli*. J. Bacteriol. 127, 76–83. 776950 10.1128/jb.127.1.76-83.1976 PMC233035

[B172] Lamed R. Zeikus J. G. (1981). Thermostable, ammonium-activated malic enzyme of *Clostridium thermocellum*. Biochim. Biophys. Acta 660, 251–255. 10.1016/0005-2744(81)90167-4 7284402

[B173] Lampel K. A. Uratani B. Chaudhry G. R. Ramaley R. F. Rudikoff S. (1986). Characterization of the developmentally regulated *Bacillus subtilis* glucose dehydrogenase gene. J. Bacteriol. 166, 238–243. 3082854 10.1128/jb.166.1.238-243.1986 PMC214582

[B174] Landete J. M. Garcia-Haro L. Blasco A. Manzanares P. Berbegal C. Monedero V.. (2010). Requirement of the *Lactobacillus casei* MaeKR two-component system for L-malic acid utilization via a malic enzyme pathway. Appl. Environ. Microbiol. 76, 84–95. 10.1128/AEM.02145-09 19897756 PMC2798650

[B175] Lauterbach L. Lenz O. Vincent K. A. (2013). H(2)-driven cofactor regeneration with NAD(P)(+)-reducing hydrogenases. FEBS J. 280, 3058–3068. 10.1111/febs.12245 23497170

[B176] Lee H. C. (1997). Mechanisms of calcium signaling by cyclic ADP-ribose and NAADP. Physiol. Rev. 77, 1133–1164. 9354813 10.1152/physrev.1997.77.4.1133

[B177] Lee H. C. Kim J. S. Jang W. Kim S. Y. (2009). Thymidine production by overexpressing NAD+ kinase in an *Escherichia coli* recombinant strain. Biotechnol. Lett. 31, 1929–1936. 10.1007/s10529-009-0097-z 19774345

[B178] Lee H. C. Kim J. S. Jang W. Kim S. Y. (2010). High NADPH/NADP+ ratio improves thymidine production by a metabolically engineered *Escherichia coli* strain. J. Biotechnol. 149, 24–32. 10.1016/j.jbiotec.2010.06.011 20600382

[B179] Lee J.-N. Shin H.-D. Lee Y.-H. (2003). Metabolic engineering of pentose phosphate pathway in *Ralstonia eutropha* for enhanced biosynthesis of poly-β-hydroxybutyrate. Biotechnol. Prog. 19, 1444–1449. 10.1021/bp034060v 14524705

[B180] Lee W.-H. Chin Y.-W. Han N. S. Kim M.-D. Seo J.-H. (2011). Enhanced production of GDP-L-fucose by overexpression of NADPH regenerator in recombinant *Escherichia coli*. Appl. Microbiol. Biotechnol. 91, 967–976. 10.1007/s00253-011-3271-x 21538115

[B181] Lee W.-H. Kim J.-W. Park E.-H. Han N. S. Kim M.-D. Seo J.-H. (2013a). Effects of NADH kinase on NADPH-dependent biotransformation processes in *Escherichia coli*. Appl. Microbiol. Biotechnol. 97, 1561–1569. 10.1007/s00253-012-4431-3 23053084

[B182] Lee W. H. Kim M. D. Jin Y. S. Seo J. H. (2013b). Engineering of NADPH regenerators in *Escherichia coli* for enhanced biotransformation. Appl. Microbiol. Biotechnol. 97, 2761–2772. 10.1007/s00253-013-4750-z 23420268

[B183] Lee W.-H. Park J.-B. Park K. Kim M.-D. Seo J.-H. (2007a). Enhanced production of ε-caprolactone by overexpression of NADPH-regenerating glucose 6-phosphate dehydrogenase in recombinant *Escherichia coli* harboring cyclohexanone monooxygenase gene. Appl. Microbiol. Biotechnol. 76, 329–338. 10.1007/s00253-007-1016-7 17541782

[B184] Lee Y. Yeom J. Kang Y.-S. Kim J. Sung J.-S. Jeon C. O.. (2007b). Molecular characterization of fprB (ferredoxin-NADP+ reductase) in *Pseudomonas putida* KT2440. J. Microbiol. Biotechnol. 17, 1504–1512. 18062229

[B185] Lerondel G. Doan T. Zamboni N. Sauer U. Aymerich S. (2006). YtsJ has the major physiological role of the four paralogous malic enzyme isoforms in *Bacillus subtilis*. J. Bacteriol. 188, 4727–4736. 10.1128/JB.00167-06 16788182 PMC1482987

[B186] Lever T. M. Palmer T. Cunningham I. J. Cotton N. P. Jackson J. B. (1991). Purification and properties of the H(+)-nicotinamide nucleotide transhydrogenase from *Rhodobacter capsulatus*. Eur. J. Biochem. 197, 247–255. 10.1111/j.1432-1033.1991.tb15905.x 1849819

[B187] Levy H. R. (1979). Glucose-6-phosphate dehydrogenases. Adv. Enzymol. Relat. Areas Mol. Biol. 48, 97–192. 10.1002/9780470122938.ch3 367106

[B188] Lewis T. A. Glassing A. Harper J. Franklin M. J. (2013). Role for ferredoxin:NAD(P)H oxidoreductase (FprA) in sulfate assimilation and siderophore biosynthesis in Pseudomonads. J. Bacteriol. 195, 3876–3887. 10.1128/JB.00528-13 23794620 PMC3754593

[B189] Li Z.-J. Cai L. Wu Q. Chen G.-Q. (2009). Overexpression of NAD kinase in recombinant *Escherichia col*i harboring the phbCAB operon improves poly(3-hydroxybutyrate) production. Appl. Microbiol. Biotechnol. 83, 939–947. 10.1007/s00253-009-1943-6 19357844

[B190] Lim S.-J. Jung Y.-M. Shin H.-D. Lee Y.-H. (2002). Amplification of the NADPH-related genes zwf and gnd for the oddball biosynthesis of PHB in an *E. coli* transformant harboring a cloned phbCAB operon. J. Biosci. Bioeng. 93, 543–549. 10.1016/S1389-1723(02)80235-3 16233247

[B191] Lindahl R. (1992). Aldehyde dehydrogenases and their role in carcinogenesis. Crit. Rev. Biochem. Mol. Biol. 27, 283–335. 10.3109/10409239209082565 1521460

[B192] Lindner S. N. Niederholtmeyer H. Schmitz K. Schoberth S. M. Wendisch V. F. (2010). Polyphosphate/ATP-dependent NAD kinase of *Corynebacterium glutamicum*: biochemical properties and impact of ppnK overexpression on lysine production. Appl. Microbiol. Biotechnol. 87, 583–593. 10.1007/s00253-010-2481-y 20180116

[B193] Lipscomb G. L. Stirrett K. Schut G. J. Yang F. Jenney F. E. Jr. Scott R. A.. (2011). Natural competence in the hyperthermophilic archaeon *Pyrococcus furiosus* facilitates genetic manipulation: construction of markerless deletions of genes encoding the two cytoplasmic hydrogenases. Appl. Environ. Microbiol. 77, 2232–2238. 10.1128/AEM.02624-10 21317259 PMC3067412

[B194] Liu W. Wang P. (2007). Cofactor regeneration for sustainable enzymatic biosynthesis. Biotechnol. Adv. 25, 369–384. 10.1016/j.biotechadv.2007.03.002 17459647

[B195] Lloyd A. J. Weitzman D. (1988). Purification and characterization of NAD-linked isocitrate dehydrogenase from *Methylophilus methylotrophus*. Biochem. Soc. Trans. 16, 871–872. 10.1042/bst0160871

[B196] London J. Meyer E. Y. Kulczyk S. (1971). Comparative biochemical and immunological study of malic enzyme from two species of lactic acid bacteria: evolutionary implications. J. Bacteriol. 106, 126–137. 4323962 10.1128/jb.106.1.126-137.1971 PMC248652

[B197] Lorentzen E. Hensel R. Knura T. Ahmed H. Pohl E. (2004). Structural Basis of allosteric regulation and substrate specificity of the non-phosphorylating glyceraldehyde 3-phosphate dehydrogenase from *Thermoproteus tenax*. J. Mol. Biol. 341, 815–828. 10.1016/j.jmb.2004.05.032 15288789

[B198] Lu Y. Mei L. (2007). Co-expression of P450 BM3 and glucose dehydrogenase by recombinant *Escherichia coli* and its application in an NADPH-dependent indigo production system. J. Ind. Microbiol. Biotechnol. 34, 247–253. 10.1007/s10295-006-0193-1 17171348

[B199] Lyon E. J. Shima S. Buurman G. Chowdhuri S. Batschauer A. Steinbach K.. (2004). UV-A/blue-light inactivation of the 'metal-free' hydrogenase (Hmd) from methanogenic archaea. Eur. J. Biochem. 271, 195–204. 10.1046/j.1432-1033.2003.03920.x 14686932

[B200] Ma K. Adams M. W. (2001a). Ferredoxin:NADP oxidoreductase from *Pyrococcus furiosus*. Meth. Enzymol. 334, 40–45. 10.1016/S0076-6879(01)34456-7 11398480

[B201] Ma K. Adams M. W. W. (2001b). Hydrogenases I and II from *Pyrococcus furiosus*, in Methods in Enzymology, eds Michael R. M. K. Adams W. W. (Academic Press), 208–216. 10.1016/s0076-6879(01)31059-5 11265463

[B202] Ma K. Weiss R. Adams M. W. W. (2000). Characterization of hydrogenase II from the hyperthermophilic archaeon *Pyrococcus furiosus* and assessment of its role in sulfur reduction. J. Bacteriol. 182, 1864–1871. 10.1128/JB.182.7.1864-1871.2000 10714990 PMC101868

[B203] Magni G. Amici A. Emanuelli M. Orsomando G. Raffaelli N. Ruggieri S. (2004). Enzymology of NAD+ homeostasis in man. Cell. Mol. Life Sci. 61, 19–34. 10.1007/s00018-003-3161-1 14704851 PMC11138864

[B204] Magni G. Amici A. Emanuelli M. Raffaelli N. Ruggieri S. (1999). Enzymology of NAD+ synthesis. Adv. Enzymol. Relat. Areas Mol. Biol. 73, 135–182. 10.1002/9780470123195.ch5 10218108

[B205] Magni G. Orsomando G. Raffaelli N. (2006). Structural and functional properties of NAD kinase, a key enzyme in NADP biosynthesis. Mini Rev. Med. Chem. 6, 739–746. 10.2174/138955706777698688 16842123

[B206] Mailloux R. J. Bériault R. Lemire J. Singh R. Chénier D. R. Hamel R. D.. (2007). The tricarboxylic acid cycle, an ancient metabolic network with a novel twist. PLoS ONE 2:e690. 10.1371/journal.pone.0000690 17668068 PMC1930152

[B207] Malki S. Saimmaime I. De Luca G. Rousset M. Dermoun Z. Belaich J. P. (1995). Characterization of an operon encoding an NADP-reducing hydrogenase in *Desulfovibrio fructosovorans*. J. Bacteriol. 177, 2628–2636. 7751270 10.1128/jb.177.10.2628-2636.1995 PMC176931

[B208] Marchitti S. A. Brocker C. Stagos D. Vasiliou V. (2008). Non-P450 aldehyde oxidizing enzymes: the aldehyde dehydrogenase superfamily. Exp. Opin. Drug Metab. Toxicol. 4, 697–720. 10.1517/17425255.4.6.697 18611112 PMC2658643

[B209] Martinez I. Zhu J. Lin H. Bennett G. N. San K.-Y. (2008). Replacing *Escherichia coli* NAD-dependent glyceraldehyde 3-phosphate dehydrogenase (GAPDH) with a NADP-dependent enzyme from *Clostridium acetobutylicum* facilitates NADPH dependent pathways. Metab. Eng. 10, 352–359. 10.1016/j.ymben.2008.09.001 18852061

[B210] Martinez-Julvez M. Medina M. Velazquez-Campoy A. (2009). Binding thermodynamics of ferredoxin:NADP+ reductase: two different protein substrates and one energetics. Biophys. J. 96, 4966–4975. 10.1016/j.bpj.2009.02.061 19527656 PMC2712046

[B211] Marx A. Eikmanns B. J. Sahm H. De Graaf A. A. Eggeling L. (1999). Response of the central metabolism in *Corynebacterium glutamicum* to the use of an NADH-dependent glutamate dehydrogenase. Metab. Eng. 1, 35–48. 10.1006/mben.1998.0106 10935753

[B212] Marx A. Hans S. Mockel B. Bathe B. De Graaf A. A. Mccormack A. C.. (2003). Metabolic phenotype of phosphoglucose isomerase mutants of *Corynebacterium glutamicum*. J. Biotechnol. 104, 185–197. 10.1016/S0168-1656(03)00153-6 12948638

[B213] Mateos M. Serrano A. (1992). Occurrence of phosphorylating and non-phosphorylating NADP+-dependent glyceraldehyde-3-phosphate dehydrogenases in photosynthetic organisms. Plant Sci. 84, 163–170. 10.1016/0168-9452(92)90130-E1764059

[B214] Mather L. H. Knight M. (1972). A heat-stable nicotinamide-adenine dinucleotide glycohydrolase from *Pseudomonas putida* KB1. Partial purification and some properties of the enzyme and an inhibitory protein. Biochem. J. 129, 141–152. 4345853 10.1042/bj1290141 PMC1174050

[B215] Matsubara K. Yokooji Y. Atomi H. Imanaka T. (2011). Biochemical and genetic characterization of the three metabolic routes in *Thermococcus kodakarensis* linking glyceraldehyde 3-phosphate and 3-phosphoglycerate. Mol. Microbiol. 81, 1300–1312. 10.1111/j.1365-2958.2011.07762.x 21736643

[B216] Matsuda F. Ishii J. Kondo T. Ida K. Tezuka H. Kondo A. (2013). Increased isobutanol production in *Saccharomyces cerevisiae* by eliminating competing pathways and resolving cofactor imbalance. Microb. Cell Fact. 12:119. 10.1186/1475-2859-12-119 24305546 PMC3866936

[B217] Matula T. I. Mcdonald I. J. Martin S. M. (1969). CO2 fixation by malic enzyme in a species of Micrococcus. Biochem. Biophys. Res. Commun. 34, 795–802. 10.1016/0006-291X(69)90250-2 4388574

[B218] Mcdermott T. R. Kahn M. L. (1992). Cloning and mutagenesis of the *Rhizobium meliloti* isocitrate dehydrogenase gene. J. Bacteriol. 174, 4790–4797. 1320616 10.1128/jb.174.14.4790-4797.1992 PMC206277

[B219] Mcguinness E. T. Butler J. R. (1985). NAD+ kinase–a review. Int. J. Biochem. 17, 1–11. 10.1016/0020-711X(85)90079-5 2987053

[B220] Medina M. Gomez-Moreno C. (2004). Interaction of Ferredoxin-NADP(+) Reductase with its substrates: optimal interaction for efficient electron transfer. Photosyn. Res. 79, 113–131. 10.1023/B:PRES.0000015386.67746.2c 16228387

[B221] Mertens R. Greiner L. Van Den Ban E. C. D. Haaker H. B. C. M. Liese A. (2003). Practical applications of hydrogenase I from *Pyrococcus furiosus* for NADPH generation and regeneration. J. Mol. Catal. B 24–25, 39–52. 10.1016/S1381-1177(03)00071-7

[B222] Meyer F. M. Stulke J. (2013). Malate metabolism in *Bacillus subtilis*: distinct roles for three classes of malate-oxidizing enzymes. FEMS Microbiol. Lett. 339, 17–22. 10.1111/1574-6968.12041 23136871

[B223] Middleditch L. E. Atchison R. W. Chung A. E. (1972). Pyridine nucleotide transhydrogenase from *Azotobacter vinelandii*. Some aspects of its structure. J. Biol. Chem. 247, 6802–6809. 4404238

[B224] Miller W. L. Auchus R. J. (2011). The molecular biology, biochemistry, and physiology of human steroidogenesis and its disorders. Endocr. Rev. 32, 81–151. 10.1210/er.2010-0013 21051590 PMC3365799

[B225] Minard K. I. Mcalister-Henn L. (2005). Sources of NADPH in yeast vary with carbon source. J. Biol. Chem. 280, 39890–39896. 10.1074/jbc.M509461200 16179340

[B226] Moreira Dos Santos M. Raghevendran V. Kotter P. Olsson L. Nielsen J. (2004). Manipulation of malic enzyme in *Saccharomyces cerevisiae* for increasing NADPH production capacity aerobically in different cellular compartments. Metab. Eng. 6, 352–363. 10.1016/j.ymben.2004.06.002 15491864

[B227] Mori S. Kawai S. Shi F. Mikami B. Murata K. (2005). Molecular conversion of NAD kinase to NADH kinase through single amino acid residue substitution. J. Biol. Chem. 280, 24104–24112. 10.1074/jbc.M502518200 15855156

[B228] Morimoto Y. Honda K. Ye X. Okano K. Ohtake H. (2014). Directed evolution of thermotolerant malic enzyme for improved malate production. J. Biosci. Bioeng. 117, 147–152. 10.1016/j.jbiosc.2013.07.005 23932397

[B229] Moritz B. Striegel K. De Graaf A. A. Sahm H. (2000). Kinetic properties of the glucose-6-phosphate and 6-phosphogluconate dehydrogenases from *Corynebacterium glutamicum* and their application for predicting pentose phosphate pathway flux *in vivo*. Eur. J. Biochem. 267, 3442–3452. 10.1046/j.1432-1327.2000.01354.x 10848959

[B230] Mulo P. (2011). Chloroplast-targeted ferredoxin-NADP(+) oxidoreductase (FNR): structure, function and location. Biochim. Biophys. Acta 1807, 927–934. 10.1016/j.bbabio.2010.10.001 20934402

[B231] Munekage Y. Hashimoto M. Miyake C. Tomizawa K.-I. Endo T. Tasaka M.. (2004). Cyclic electron flow around photosystem I is essential for photosynthesis. Nature 429, 579–582. 10.1038/nature02598 15175756

[B232] Murakami K. Iwata S. Haneda M. Yoshino M. (1997). Role of metal cations in the regulation of NADP-linked isocitrate dehydrogenase from porcine heart. Biometals 10, 169–174. 10.1023/A:1018395510334 9243796

[B233] Muraki N. Seo D. Shiba T. Sakurai T. Kurisu G. (2010). Asymmetric dimeric structure of ferredoxin-NAD(P)+ oxidoreductase from the green sulfur bacterium *Chlorobaculum tepidum*: implications for binding ferredoxin and NADP+. J. Mol. Biol. 401, 403–414. 10.1016/j.jmb.2010.06.024 20600130

[B234] Muro-Pastor M. I. Florencio F. J. (1994). NADP (+)-isocitrate dehydrogenase from the *Cyanobacterium Anabaena* sp. strain PCC 7120: purification and characterization of the enzyme and cloning, sequencing, and disruption of the icd gene. J. Bacteriol. 176, 2718–2726. 8169222 10.1128/jb.176.9.2718-2726.1994 PMC205413

[B235] Muro-Pastor M. I. Reyes J. C. Florencio F. J. (2005). Ammonium assimilation in cyanobacteria. Photosyn. Res. 83, 135–150. 10.1007/s11120-004-2082-7 16143848

[B236] Musumeci M. A. Ceccarelli E. A. Catalano-Dupuy D. L. (2012). The plant–type ferredoxin-NADP+ reductases, in Advances in Photosynthesis - Fundamental Aspects, ed Najafpour D. M. (Rijeka: InTech), 539–562. 10.5772/28665

[B237] Nakamichi Y. Yoshioka A. Kawai S. Murata K. (2013). Conferring the ability to utilize inorganic polyphosphate on ATP-specific NAD kinase. Sci. Rep. 3, 2632. 10.1038/srep02632 24022322 PMC3769651

[B238] Nakamura M. Bhatnagar A. Sadoshima J. (2012). Overview of pyridine nucleotides review series. Circ. Res. 111, 604–610. 10.1161/CIRCRESAHA.111.247924 22904040 PMC3523884

[B239] Netzer R. Krause M. Rittmann D. Peters-Wendisch P. G. Eggeling L. Wendisch V. F.. (2004). Roles of pyruvate kinase and malic enzyme in *Corynebacterium glutamicum* for growth on carbon sources requiring gluconeogenesis. Arch. Microbiol. 182, 354–363. 10.1007/s00203-004-0710-4 15375646

[B240] Nicolas C. Kiefer P. Letisse F. Kromer J. Massou S. Soucaille P.. (2007). Response of the central metabolism of *Escherichia coli* to modified expression of the gene encoding the glucose-6-phosphate dehydrogenase. FEBS Lett. 581, 3771–3776. 10.1016/j.febslet.2007.06.066 17631881

[B241] Noor E. Bar-Even A. Flamholz A. Lubling Y. Davidi D. Milo R. (2012). An integrated open framework for thermodynamics of reactions that combines accuracy and coverage. Bioinformatics 28, 2037–2044. 10.1093/bioinformatics/bts317 22645166 PMC3400964

[B242] Noor E. Haraldsdóttir H. S. Milo R. Fleming R. M. T. (2013). Consistent estimation of gibbs energy using component contributions. PLoS Comput. Biol. 9:e1003098. 10.1371/journal.pcbi.1003098 23874165 PMC3708888

[B243] Nordberg J. Arnér E. S. J. (2001). Reactive oxygen species, antioxidants, and the mammalian thioredoxin system. Free Radic. Biol. Med. 31, 1287–1312. 10.1016/S0891-5849(01)00724-9 11728801

[B244] Nouailler M. Morelli X. Bornet O. Chetrit B. Dermoun Z. Guerlesquin F. (2006). Solution structure of HndAc: a thioredoxin-like domain involved in the NADP-reducing hydrogenase complex. Protein Sci. 15, 1369–1378. 16731971 10.1110/ps.051916606 PMC2242533

[B245] Obanye A. I. C. Hobbs G. Gardner D. C. J. Oliver S. G. (1996). Correlation between carbon flux through the pentose phosphate pathway and production of the antibiotic methylenomycin in *Streptomyces coelicolor* A3(2). Microbiology (Reading Engl). 142, 133–137. 10.1099/13500872-142-1-133 33657736

[B246] Ochiai A. Mori S. Kawai S. Murata K. (2004). Overexpression, purification, and characterization of ATP-NAD kinase of *Sphingomonas* sp. A1. Protein Expr. Purif. 36, 124–130. 10.1016/j.pep.2004.03.012 15177293

[B247] Ogawa T. Murakami K. Mori H. Ishii N. Tomita M. Yoshin M. (2007). Role of phosphoenolpyruvate in the NADP-isocitrate dehydrogenase and isocitrate lyase reaction in *Escherichia coli*. J. Bacteriol. 189, 1176–1178. 10.1128/JB.01628-06 17142397 PMC1797289

[B248] Oh M.-K. Rohlin L. Kao K. C. Liao J. C. (2002). Global expression profiling of acetate-grown *Escherichia coli*. J. Biol. Chem. 277, 13175–13183. 10.1074/jbc.M110809200 11815613

[B249] Olavarría K. Valdés D. Cabrera R. (2012). The cofactor preference of glucose-6-phosphate dehydrogenase from *Escherichia coli* – modeling the physiological production of reduced cofactors. FEBS J. 279, 2296–2309. 10.1111/j.1742-4658.2012.08610.x 22519976

[B250] Onda Y. Matsumura T. Kimata-Ariga Y. Sakakibara H. Sugiyama T. Hase T. (2000). Differential interaction of maize root ferredoxin:NADP(+) oxidoreductase with photosynthetic and non-photosynthetic ferredoxin isoproteins. Plant Physiol. 123, 1037–1045. 10.1104/pp.123.3.1037 10889253 PMC59067

[B251] Outten C. E. Culotta V. C. (2003). A novel NADH kinase is the mitochondrial source of NADPH in *Saccharomyces cerevisiae*. EMBO J. 22, 2015–2024. 10.1093/emboj/cdg211 12727869 PMC156083

[B252] Owen O. E. Kalhan S. C. Hanson R. W. (2002). The key role of anaplerosis and cataplerosis for citric acid cycle function. J. Biol. Chem. 277, 30409–30412. 10.1074/jbc.R200006200 12087111

[B253] Papagianni M. (2012). Recent advances in engineering the central carbon metabolism of industrially important bacteria. Microb. Cell Fact. 11:50. 10.1186/1475-2859-11-50 22545791 PMC3461431

[B254] Park H. J. Jung J. Choi H. Uhm K.-N. Kim H. K. (2010). Enantioselective bioconversion using *Escherichia coli* cells expressing *Saccharomyces cerevisiae* reductase and *Bacillus subtilis* glucose dehydrogenase. J. Microbiol. Biotechnol. 20, 1300–1306. 10.4014/jmb.1003.03025 20890095

[B255] Patel S. Ramakrishnan L. Rahman T. Hamdoun A. Marchant J. S. Taylor C. W.. (2011). The endo-lysosomal system as an NAADP-sensitive acidic Ca(2+) store: role for the two-pore channels. Cell Calcium 50, 157–167. 10.1016/j.ceca.2011.03.011 21529939 PMC3160778

[B256] Paul L. Mishra P. Blumenthal R. Matthews R. (2007). Integration of regulatory signals through involvement of multiple global regulators: control of the *Escherichia coli* gltBDF operon by Lrp, IHF, Crp, and ArgR. BMC Microbiol. 7:2. 10.1186/1471-2180-7-2 17233899 PMC1784095

[B257] Pauly H. E. Pfleiderer G. (1975). D-glucose dehydrogenase from *Bacillus megaterium* M 1286: purification, properties and structure. Hoppe-Seyler's Z. Physiol. Chem. 356, 1613–1623. 10.1515/bchm2.1975.356.2.1613 2530

[B258] Pedersen A. Karlsson G. B. Rydstrom J. (2008). Proton-translocating transhydrogenase: an update of unsolved and controversial issues. J. Bioenerg. Biomembr. 40, 463–473. 10.1007/s10863-008-9170-x 18972197

[B259] Penyige A. Deak E. Kalmanczhelyi A. Barabas G. (1996). Evidence of a role for NAD+-glycohydrolase and ADP-ribosyltransferase in growth and differentiation of *Streptomyces griseus* NRRL B-2682: inhibition by m-aminophenylboronic acid. Microbiology 142(Pt 8), 1937–1944. 10.1099/13500872-142-8-1937 8800814

[B260] Peralta-Yahya P. P. Zhang F. Del Cardayre S. B. Keasling J. D. (2012). Microbial engineering for the production of advanced *Biofuels* 488, 320–328. 10.1038/nature11478 22895337

[B261] Peters J. W. Schut G. J. Boyd E. S. Mulder D. W. Shepard E. M. Broderick J. B.. (2015). [FeFe]- and [NiFe]-hydrogenase diversity, mechanism, and maturation. Biochim. Biophys. Acta Mol. Cell Res. 1853, 1350–1369. 10.1016/j.bbamcr.2014.11.021 25461840

[B262] Petersen S. De Graaf A. A. Eggeling L. Möllney M. Wiechert W. Sahm H. (2000). *In vivo* quantification of parallel and bidirectional fluxes in the anaplerosis of *Corynebacterium glutamicum*. J. Biol. Chem. 275, 35932–35941. 10.1074/jbc.M908728199 10946002

[B263] Pollak N. Dölle C. Ziegler M. (2007). The power to reduce: pyridine nucleotides – small molecules with a multitude of functions. Biochem. J. 402, 205–218. 10.1042/BJ20061638 17295611 PMC1798440

[B264] Poncet-Montange G. Assairi L. Arold S. Pochet S. Labesse G. (2007). NAD kinases use substrate-assisted catalysis for specific recognition of NAD. J. Biol. Chem. 282, 33925–33934. 10.1074/jbc.M701394200 17686780

[B265] Purwantini E. Daniels L. (1996). Purification of a novel coenzyme F420-dependent glucose-6-phosphate dehydrogenase from *Mycobacterium smegmatis*. J. Bacteriol. 178, 2861–2866. 8631674 10.1128/jb.178.10.2861-2866.1996 PMC178021

[B266] Purwantini E. Gillis T. P. Daniels L. (1997). Presence of F420-dependent glucose-6-phosphate dehydrogenase in *Mycobacterium* and *Nocardia* species, but absence from *Streptomyces* and *Corynebacterium* species and methanogenic Archaea. FEMS Microbiol. Lett. 146, 129–134. 10.1111/j.1574-6968.1997.tb10182.x 8997717

[B267] Qi H. Li S. Zhao S. Huang D. Xia M. Wen J. (2014). Model-driven redox pathway manipulation for improved isobutanol production in *Bacillus subtilis* complemented with experimental validation and metabolic profiling analysis. PLoS ONE 9:e93815. 10.1371/journal.pone.0093815 24705866 PMC3976320

[B268] Rahman M. M. Q. Zhao Q. Dou W. Zhiming R. Xu Z. (2012). Over-expression of NAD kinase in *Corynebacterium crenatum* and its impact on L-Arginine biosynthesis. Tropic. J. Pharm. Res. 11, 909–916. 10.4314/tjpr.v11i6.6

[B269] Rathnasingh C. Raj S. M. Lee Y. Catherine C. Ashok S. Park S. (2012). Production of 3-hydroxypropionic acid via malonyl-CoA pathway using recombinant *Escherichia coli* strains. J. Biotechnol. 157, 633–640. 10.1016/j.jbiotec.2011.06.008 21723339

[B270] Razquin P. Fillat M. F. Schmitz S. Stricker O. Bohme H. Gomez-Moreno C.. (1996). Expression of ferredoxin-NADP+ reductase in heterocysts from *Anabaena* sp. Biochem. J. 316(Pt 1), 157–160. 8645199 10.1042/bj3160157 PMC1217316

[B271] Reddy G. K. Lindner S. N. Wendisch V. F. (2015). Metabolic Engineering an ATP-neutral EMP pathway in *Corynebacterium glutamicum*: adaptive point mutation in NADH dehydrogenase restores growth. Appl. Environ. Microbiol. 81, 1996–2005. 10.1128/AEM.03116-14 25576602 PMC4345364

[B272] Reidl J. Schlor S. Kraiss A. Schmidt-Brauns J. Kemmer G. Soleva E. (2000). NADP and NAD utilization in *Haemophilus influenzae*. Mol. Microbiol. 35, 1573–1581. 10.1046/j.1365-2958.2000.01829.x 10760156

[B273] Richhardt J. Bringer S. Bott M. (2013). Role of the pentose phosphate pathway and the Entner-Doudoroff pathway in glucose metabolism of *Gluconobacter oxydans* 621H. Appl. Microbiol. Biotechnol. 97, 4315–4323. 10.1007/s00253-013-4707-2 23354449

[B274] Riedel C. Rittmann D. Dangel P. Mockel B. Petersen S. Sahm H.. (2001). Characterization of the phosphoenolpyruvate carboxykinase gene from *Corynebacterium glutamicum* and significance of the enzyme for growth and amino acid production. J. Mol. Microbiol. Biotechnol. 3, 573–583. 11565516

[B275] Poulsen B. R. Nohr J. Douthwaite S. Hansen L. V. Iversen J. J. L. Visser J.. (2005). Increased NADPH concentration obtained by metabolic engineering of the pentose phosphate pathway in *Aspergillus niger*. FEBS J. 272, 1313–1325. 10.1111/j.1742-4658.2005.04554.x 15752350

[B276] Rydzak T. Grigoryan M. Cunningham Z. J. Krokhin O. V. Ezzati P. Cicek N.. (2014). Insights into electron flux through manipulation of fermentation conditions and assessment of protein expression profiles in *Clostridium thermocellum*. Appl. Microbiol. Biotechnol. 98, 6497–6510. 10.1007/s00253-014-5798-0 24841118

[B277] Sahm H. Eggeling L. De Graaf A. A. (2000). Pathway analysis and metabolic engineering in *Corynebacterium glutamicum*. Biol. Chem. 381, 899–910. 10.1515/BC.2000.111 11076021

[B278] Sakuraba H. Kawakami R. Ohshima T. (2005). First Archaeal Inorganic Polyphosphate/ATP-Dependent NAD Kinase, from Hyperthermophilic Archaeon *Pyrococcus horikoshii*: cloning, expression, and characterization. Appl. Environ. Microbiol. 71, 4352–4358. 10.1128/AEM.71.8.4352-4358.2005 16085824 PMC1183369

[B279] Sakuraba H. Satomura T. Kawakami R. Yamamoto S. Kawarabayasi Y. Kikuchi H.. (2002). L-Aspartate oxidase is present in the anaerobic hyperthermophilic archaeon *Pyrococcus horikoshii* OT-3: characteristics and role in the *de novo* biosynthesis of nicotinamide adenine dinucleotide proposed by genome sequencing. Extremophiles 6, 275–281. 10.1007/s00792-001-0254-3 12215812

[B280] Saliola M. Tramonti A. Lanini C. Cialfi S. De Biase D. Falcone C. (2012). Intracellular NADPH levels affect the oligomeric state of the glucose 6-phosphate dehydrogenase. Eukaryot. Cell 11, 1503–1511. 10.1128/EC.00211-12 23064253 PMC3536282

[B281] Sanchez A. M. Andrews J. Hussein I. Bennett G. N. San K.-Y. (2006). Effect of overexpression of a soluble pyridine nucleotide transhydrogenase (UdhA) on the production of poly(3-hydroxybutyrate) in *Escherichia coli*. Biotechnol. Prog. 22, 420–425. 10.1021/bp050375u 16599556

[B282] Sancho J. (2006). Flavodoxins: sequence, folding, binding, function and beyond. Cell. Mol. Life Sci. 63, 855–864. 10.1007/s00018-005-5514-4 16465441 PMC11136378

[B283] Santangelo T. J. Cubonova L. Reeve J. N. (2011). Deletion of alternative pathways for reductant recycling in *Thermococcus kodakarensis* increases hydrogen production. Mol. Microbiol. 81, 897–911. 10.1111/j.1365-2958.2011.07734.x 21749486 PMC3179246

[B284] Sassetti C. M. Boyd D. H. Rubin E. J. (2003). Genes required for mycobacterial growth defined by high density mutagenesis. Mol. Microbiol. 48, 77–84. 10.1046/j.1365-2958.2003.03425.x 12657046

[B285] Sato T. Atomi H. (2011). Novel metabolic pathways in Archaea. Curr. Opin. Microbiol. 14, 307–314. 10.1016/j.mib.2011.04.014 21612976

[B286] Sato Y. Kameya M. Fushinobu S. Wakagi T. Arai H. Ishii M.. (2012). A novel enzymatic system against oxidative stress in the thermophilic hydrogen-oxidizing bacterium *Hydrogenobacter thermophilus*. PLoS ONE 7:e34825. 10.1371/journal.pone.0034825 22485188 PMC3317640

[B287] Satoh Y. Tajima K. Tannai H. Munekata M. (2003). Enzyme-catalyzed poly(3-hydroxybutyrate) synthesis from acetate with CoA recycling and NADPH regeneration *in vitro*. J. Biosci. Bioeng. 95, 335–341. 10.1016/S1389-1723(03)80064-6 16233416

[B288] Sauer U. Canonaco F. Heri S. Perrenoud A. Fischer E. (2004). The soluble and membrane-bound transhydrogenases UdhA and PntAB have divergent functions in NADPH metabolism of *Escherichia coli*. J. Biol. Chem. 279, 6613–6619. 10.1074/jbc.M311657200 14660605

[B289] Sauer U. Eikmanns B. J. (2005). The PEP–pyruvate–oxaloacetate node as the switch point for carbon flux distribution in bacteria. FEMS Microbiol. Rev. 29, 765–794. 10.1016/j.femsre.2004.11.002 16102602

[B290] Schäfer T. Schönheit P. (1993). Gluconeogenesis from pyruvate in the hyperthermophilic archaeon *Pyrococcus furiosus*: involvement of reactions of the Embden-Meyerhof pathway. Arch. Microbiol. 159, 354–363. 10.1007/BF00290918

[B291] Schmitz O. Boison G. Salzmann H. Bothe H. Schutz K. Wang S.-H.. (2002). HoxE–a subunit specific for the pentameric bidirectional hydrogenase complex (HoxEFUYH) of cyanobacteria. Biochim. Biophys. Acta 1554, 66–74. 10.1016/S0005-2728(02)00214-1 12034472

[B292] Schut G. J. Nixon W. J. Lipscomb G. L. Scott R. A. Adams M. W. W. (2012). Mutational analyses of the enzymes involved in the metabolism of hydrogen by the hyperthermophilic archaeon *Pyrococcus furiosus*. Front. Microbiol. 3:163. 10.3389/fmicb.2012.00163 22557999 PMC3341082

[B293] Seeber F. Aliverti A. Zanetti G. (2005). The plant-type ferredoxin-NADP+ reductase/ferredoxin redox system as a possible drug target against apicomplexan human parasites. Curr. Pharm. Des. 11, 3159–3172. 10.2174/1381612054864957 16178751

[B294] Seo D. Kamino K. Inoue K. Sakurai H. (2004). Purification and characterization of ferredoxin-NADP+ reductase encoded by *Bacillus subtilis* yumC. Arch. Microbiol. 182, 80–89. 10.1007/s00203-004-0701-5 15252706

[B295] Seo D. Sakurai H. (2002). Purification and characterization of ferredoxin-NAD(P)(+) reductase from the green sulfur bacterium *Chlorobium tepidum*. Biochim. Biophys. Acta 1597, 123–132. 10.1016/S0167-4838(02)00269-8 12009411

[B296] Shi A. Zhu X. Lu J. Zhang X. Ma Y. (2013). Activating transhydrogenase and NAD kinase in combination for improving isobutanol production. Metab. Eng. 16, 1–10. 10.1016/j.ymben.2012.11.008 23246519

[B297] Shi F. Huan X. Wang X. Ning J. (2012). Overexpression of NAD kinases improves the L-isoleucine biosynthesis in *Corynebacterium glutamicum* ssp. lactofermentum. Enzyme Microb. Technol. 51, 73–80. 10.1016/j.enzmictec.2012.04.003 22664190

[B298] Shi F. Li Y. Wang X. (2009). Molecular properties, functions, and potential applications of NAD kinases. Acta Biochim. Biophys. Sin. (Shanghai). 41, 352–361. 10.1093/abbs/gmp029 19430699

[B299] Shiba H. Kawasumi T. Igarashi Y. Kodama T. Minoda Y. (1985). The CO2 assimilation via the reductive tricarboxylic acid cycle in an obligately autotrophic, aerobic hydrogen-oxidizing bacterium, *Hydrogenobacter thermophilus*. Arch. Microbiol. 141, 198–203. 10.1007/BF00408058

[B300] Shikanai T. (2007). Cyclic electron transport around photosystem I: genetic approaches. Annu. Rev. Plant Biol. 58, 199–217. 10.1146/annurev.arplant.58.091406.110525 17201689

[B301] Shima S. Thauer R. K. (2007). A third type of hydrogenase catalyzing H2 activation. Chem. Rec. 7, 37–46. 10.1002/tcr.20111 17304591

[B302] Shin J.-A. Kwon Y. D. Kwon O.-H. Lee H. S. Kim P. (2007). 5-Aminolevulinic acid biosynthesis in *Escherichia coli* coexpressing NADP-dependent malic enzyme and 5-aminolevulinate synthase. J. Microbiol. Biotechnol. 17, 1579–1584. 18062242

[B303] Shin M. Arnon D. I. (1965). Enzymic mechanisms of pyridine nucleotide reduction in chloroplasts. J. Biol. Chem. 240, 1405–1411. 14284756

[B304] Siebers B. Schonheit P. (2005). Unusual pathways and enzymes of central carbohydrate metabolism in Archaea. Curr. Opin. Microbiol. 8, 695–705. 10.1016/j.mib.2005.10.014 16256419

[B305] Siedler S. Bringer S. Bott M. (2011). Increased NADPH availability in *Escherichia coli*: improvement of the product per glucose ratio in reductive whole-cell biotransformation. Appl. Microbiol. Biotechnol. 92, 929–937. 10.1007/s00253-011-3374-4 21670981

[B306] Silva P. J. Van Den Ban E. C. Wassink H. Haaker H. De Castro B. Robb F. T.. (2000). Enzymes of hydrogen metabolism in *Pyrococcus furiosus*. Eur. J. Biochem. 267, 6541–6551. 10.1046/j.1432-1327.2000.01745.x 11054105

[B307] Singh R. Beriault R. Middaugh J. Hamel R. Chenier D. Appanna V. D.. (2005). Aluminum-tolerant *Pseudomonas fluorescens*: ROS toxicity and enhanced NADPH production. Extremophiles 9, 367–373. 10.1007/s00792-005-0450-7 15970995

[B308] Singh R. Lemire J. Mailloux R. J. Appanna V. D. (2008). A novel strategy involved anti-oxidative defense: the conversion of NADH into NADPH by a metabolic network. PLoS ONE 3:e2682. 10.1371/annotation/5fac086b-3806-4aa9-a5c5-2611b3355f8f 18628998 PMC2443280

[B309] Singh R. Mailloux R. J. Puiseux-Dao S. Appanna V. D. (2007). Oxidative stress evokes a metabolic adaptation that favors increased NADPH synthesis and decreased NADH production in *Pseudomonas fluorescens*. J. Bacteriol. 189, 6665–6675. 10.1128/JB.00555-07 17573472 PMC2045160

[B310] Smith K. M. Cho K.-M. Liao J. C. (2010). Engineering *Corynebacterium glutamicum* for isobutanol production. Appl. Microbiol. Biotechnol. 87, 1045–1055. 10.1007/s00253-010-2522-6 20376637 PMC2886118

[B311] Sophos N. A. Pappa A. Ziegler T. L. Vasiliou V. (2001). Aldehyde dehydrogenase gene superfamily: the 2000 update. Chem. Biol. Interact. 130–132, 323–337. 10.1016/S0009-2797(00)00275-1 11306055

[B312] Sophos N. A. Vasiliou V. (2003). Aldehyde dehydrogenase gene superfamily: the 2002 update. Chem. Biol. Interact. 143–144, 5–22. 10.1016/S0009-2797(02)00163-1 12604184

[B313] Steen I. H. Lien T. Birkeland N. K. (1997). Biochemical and phylogenetic characterization of isocitrate dehydrogenase from a hyperthermophilic archaeon, *Archaeoglobus fulgidus*. Arch. Microbiol. 168, 412–420. 10.1007/s002030050516 9325430

[B314] Steen I. H. Madern D. Karlstrom M. Lien T. Ladenstein R. Birkeland N. K. (2001). Comparison of isocitrate dehydrogenase from three hyperthermophiles reveals differences in thermostability, cofactor specificity, oligomeric state, and phylogenetic affiliation. J. Biol. Chem. 276, 43924–43931. 10.1074/jbc.M105999200 11533060

[B315] Steuber J. Krebs W. Bott M. Dimroth P. (1999). A Membrane-Bound NAD(P)(+)-Reducing hydrogenase provides reduced pyridine nucleotides during citrate fermentation by *Klebsiella pneumoniae*. J. Bacteriol. 181, 241–245. 9864336 10.1128/jb.181.1.241-245.1999 PMC103555

[B316] Stokke R. Madern D. Fedoy A.-E. Karlsen S. Birkeland N.-K. Steen I. H. (2007). Biochemical characterization of isocitrate dehydrogenase from *Methylococcus capsulatus* reveals a unique NAD+-dependent homotetrameric enzyme. Arch. Microbiol. 187, 361–370. 10.1007/s00203-006-0200-y 17160675

[B317] Stols L. Donnelly M. I. (1997). Production of succinic acid through overexpression of NAD(+)-dependent malic enzyme in an *Escherichia coli* mutant. Appl. Environ. Microbiol. 63, 2695–2701. 9212416 10.1128/aem.63.7.2695-2701.1997 PMC168564

[B318] Stournaras C. Maurer P. Kurz G. (1983). 6-phospho-D-gluconate dehydrogenase from *Pseudomonas fluorescens*. Properties and subunit structure. Eur. J. Biochem. 130, 391–396. 10.1111/j.1432-1033.1983.tb07165.x 6402366

[B319] Strand M. K. Stuart G. R. Longley M. J. Graziewicz M. A. Dominick O. C. Copeland W. C. (2003). POS5 gene of *Saccharomyces cerevisiae* encodes a mitochondrial NADH kinase required for stability of mitochondrial DNA. Eukaryot. Cell 2, 809–820. 10.1128/EC.2.4.809-820.2003 12912900 PMC178377

[B320] Summers M. L. Wallis J. G. Campbell E. L. Meeks J. C. (1995). Genetic evidence of a major role for glucose-6-phosphate dehydrogenase in nitrogen fixation and dark growth of the *cyanobacterium Nostoc* sp. strain ATCC 29133. J. Bacteriol. 177, 6184–6194. 7592384 10.1128/jb.177.21.6184-6194.1995 PMC177459

[B321] Sun J. Hopkins R. C. Jenney F. E. Jr. Mcternan P. M. Adams M. W. W. (2010). Heterologous expression and maturation of an NADP-Dependent [NiFe]-hydrogenase: a key enzyme in biofuel production. PLoS ONE 5:e10526. 10.1371/journal.pone.0010526 20463892 PMC2865534

[B322] Sung J. Y. Lee Y. N. (2007). Isoforms of glucose 6-phosphate dehydrogenase in *Deinococcus radiophilus*. J. Microbiol. 45, 318–325. 17846585

[B323] Szaszák M. Steven P. Shima K. Orzekowsky-Schröder R. Hüttmann G. König I. R.. (2011). Fluorescence lifetime imaging unravels *C. trachomatis* metabolism and its crosstalk with the host cell. PLoS Pathog 7:e1002108. 10.1371/journal.ppat.1002108 21779161 PMC3136453

[B324] Szmant H. H. (1989). Organic Building Blocks of the Chemical Industry. New York, NY: John Wiley & Sons.

[B325] Takeno S. Murata R. Kobayashi R. Mitsuhashi S. Ikeda M. (2010). Engineering of *Corynebacterium glutamicum* with an NADPH-generating glycolytic pathway for L-lysine production. Appl. Environ. Microbiol. 76, 7154–7160. 10.1128/AEM.01464-10 20851994 PMC2976228

[B326] Talfournier F. Stines-Chaumeil C. Branlant G. (2011). Methylmalonate-semialdehyde dehydrogenase from *Bacillus subtilis*: substrate specificity and coenzyme A binding. J. Biol. Chem. 286, 21971–21981. 10.1074/jbc.M110.213280 21515690 PMC3121342

[B327] Tang K.-H. Yue H. Blankenship R. E. (2010). Energy metabolism of *Heliobacterium modesticaldum* during phototrophic and chemotrophic growth. BMC Microbiol. 10:150. 10.1186/1471-2180-10-150 20497547 PMC2887804

[B328] Thanassi J. A. Hartman-Neumann S. L. Dougherty T. J. Dougherty B. A. Pucci M. J. (2002). Identification of 113 conserved essential genes using a high-throughput gene disruption system in *Streptococcus pneumoniae*. Nucleic Acids Res. 30, 3152–3162. 10.1093/nar/gkf418 12136097 PMC135739

[B329] Thauer R. K. Jungermann K. Decker K. (1977). Energy conservation in chemotrophic anaerobic bacteria. Bacteriol. Rev. 41, 100–180. 860983 10.1128/br.41.1.100-180.1977 PMC413997

[B330] Trchounian A. Gary Sawers R. (2014). Novel insights into the bioenergetics of mixed-acid fermentation: can hydrogen and proton cycles combine to help maintain a proton motive force? IUBMB Life 66, 1–7. 10.1002/iub.1236 24501007

[B331] Trchounian K. Poladyan A. Vassilian A. Trchounian A. (2012). Multiple and reversible hydrogenases for hydrogen production by *Escherichia coli*: dependence on fermentation substrate, pH and the F(0)F(1)-ATPase. Crit. Rev. Biochem. Mol. Biol. 47, 236–249. 10.3109/10409238.2012.655375 22313414

[B332] Tseng Y.-M. Harris B. G. Jacobson M. K. (1979). Isolation and characterization of yeast nicotinamide adenine dinucleotide kinase. Biochim. Biophys. Acta 568, 205–214. 10.1016/0005-2744(79)90287-0 221029

[B333] Turner W. L. Waller J. C. Snedden W. A. (2005). Identification, molecular cloning and functional characterization of a novel NADH kinase from *Arabidopsis thaliana* (thale cress). Biochem. J. 385, 217–223. 10.1042/BJ20040292 15347288 PMC1134690

[B334] Uppada V. Bhaduri S. Noronha S. B. (2014). Cofactor regeneration–an important aspect of biocatalysis. Curr. Sci. 106, 946.

[B335] Van Der Donk W. A. Zhao H. (2003). Recent developments in pyridine nucleotide regeneration. Curr. Opin. Biotechnol. 14, 421–426. 10.1016/S0958-1669(03)00094-6 12943852

[B336] Van Haaster D. J. Silva P. J. Hagedoorn P.-L. Jongejan J. A. Hagen W. R. (2008). Reinvestigation of the steady-state kinetics and physiological function of the soluble NiFe-hydrogenase I of *Pyrococcus furiosus*. J. Bacteriol. 190, 1584–1587. 10.1128/JB.01562-07 18156274 PMC2258664

[B337] Vasiliou V. Pappa A. Petersen D. R. (2000). Role of aldehyde dehydrogenases in endogenous and xenobiotic metabolism. Chem. Biol. Interact. 129, 1–19. 10.1016/S0009-2797(00)00211-8 11154732

[B338] Veech R. L. Eggleston L. V. Krebs H. A. (1969). The redox state of free nicotinamide–adenine dinucleotide phosphate in the cytoplasm of rat liver. Biochem. J. 115, 609–619. 4391039 10.1042/bj1150609a PMC1185185

[B339] Velayudhan J. Kelly D. J. (2002). Analysis of gluconeogenic and anaplerotic enzymes in *Campylobacter jejuni*: an essential role for phosphoenolpyruvate carboxykinase. Microbiology 148, 685–694. 11882702 10.1099/00221287-148-3-685

[B340] Vignais P. M. Billoud B. (2007). Occurrence, classification, and biological function of hydrogenases: an overview. Chem. Rev. 107, 4206–4272. 10.1021/cr050196r 17927159

[B341] Vignais P. M. Billoud B. Meyer J. (2001). Classification and phylogeny of hydrogenases. FEMS Microbiol. Rev. 25, 455–501. 10.1111/j.1574-6976.2001.tb00587.x 11524134

[B342] Vignais P. M. Colbeau A. (2004). Molecular biology of microbial hydrogenases. Curr. Issues Mol. Biol. 6, 159–188. 15119826

[B343] Voegele R. T. Mitsch M. J. Finan T. M. (1999). Characterization of two members of a novel malic enzyme class. Biochim. Biophys. Acta 1432, 275–285. 10.1016/S0167-4838(99)00112-0 10407149

[B344] Voordouw G. Hagen W. R. Kruse-Wolters K. M. Van Berkel-Arts A. Veeger C. (1987). Purification and characterization of *Desulfovibrio vulgaris* (Hildenborough) hydrogenase expressed in *Escherichia coli*. Eur. J. Biochem. 162, 31–36. 10.1111/j.1432-1033.1987.tb10537.x 3028789

[B345] Voordouw G. van der Vies S. M. Themmen A. P. (1983). Why are two different types of pyridine nucleotide transhydrogenase found in living organisms? Eur. J. Biochem. 131, 527–533. 6840064 10.1111/j.1432-1033.1983.tb07293.x

[B346] Voordouw G. van der Vies S. Scholten J. W. Veeger C. (1980). Pyridine nucleotide transhydrogenase from *Azotobacter vinelandii*. Eur. J. Biochem. 107, 337–344. 10.1111/j.1432-1033.1980.tb06034.x 7398644

[B347] Voordouw G. Veeger C. Van Breemen J. F. L. Van Bruggen E. F. J. (1979). Structure of pyridine nucleotide transhydrogenase from *Azotobacter vinelandii*. Eur. J. Biochem. 98, 447–454. 10.1111/j.1432-1033.1979.tb13205.x 39756

[B348] Vu C. Q. Lu P. J. Ghen C. S. Jacobson M. K. (1996). 2′-phospho-cyclic ADP-ribose, a calcium-mobilizing agent derived from NADP. J. Biol. Chem. 271, 4747–4754. 10.1074/jbc.271.9.4747 8617741

[B349] Walsh K. Koshland D. E. (1985). Branch point control by the phosphorylation state of isocitrate dehydrogenase. a quantitative examination of fluxes during a regulatory transition. J. Biol. Chem. 260, 8430–8437. 2861202

[B350] Wandrey C. (2004). Biochemical reaction engineering for redox reactions. Chem. Rec. 4, 254–265. 10.1002/tcr.20016 15340910

[B351] Wang B. Wang P. Zheng E. Chen X. Zhao H. Song P.. (2011a). Biochemical properties and physiological roles of NADP-dependent malic enzyme in *Escherichia coli*. J. Microbiol. 49, 797–802. 10.1007/s12275-011-0487-5 22068497

[B352] Wang P. Jin M. Su R. Song P. Wang M. Zhu G. (2011b). Enzymatic characterization of isocitrate dehydrogenase from an emerging zoonotic pathogen *Streptococcus suis*. Biochimie 93, 1470–1475. 10.1016/j.biochi.2011.04.021 21586311

[B353] Wang P. Jin M. Zhu G. (2012). Biochemical and molecular characterization of NAD(+)-dependent isocitrate dehydrogenase from the ethanologenic bacterium *Zymomonas mobilis*. FEMS Microbiol. Lett. 327, 134–141. 10.1111/j.1574-6968.2011.02467.x 22117777

[B354] Wang P. Song P. Jin M. Zhu G. (2013a). Isocitrate dehydrogenase from *Streptococcus mutans*: biochemical properties and evaluation of a putative phosphorylation Site at Ser102. PLoS ONE 8:e58918. 10.1371/journal.pone.0058918 23484056 PMC3590139

[B355] Wang S. Huang H. Kahnt J. Mueller A. P. Kopke M. Thauer R. K. (2013b). NADP-specific electron-bifurcating [FeFe]-hydrogenase in a functional complex with formate dehydrogenase in *Clostridium autoethanogenum* grown on CO. J. Bacteriol. 195, 4373–4386. 10.1128/JB.00678-13 23893107 PMC3807470

[B356] Wang S. Huang H. Moll J. Thauer R. K. (2010). NADP+ reduction with reduced ferredoxin and NADP+ Reduction with NADH are coupled via an electron-bifurcating enzyme complex in *Clostridium kluyveri*. J. Bacteriol. 192, 5115–5123. 10.1128/JB.00612-10 20675474 PMC2944534

[B357] Wang Y. San K.-Y. Bennett G. N. (2013c). Improvement of NADPH bioavailability in *Escherichia coli* by replacing NAD(+)-dependent glyceraldehyde-3-phosphate dehydrogenase GapA with NADP (+)-dependent GapB from *Bacillus subtilis* and addition of NAD kinase. J. Ind. Microbiol. Biotechnol. 40, 1449–1460. 10.1007/s10295-013-1335-x 24048943

[B358] Wang Y. San K.-Y. Bennett G. N. (2013d). Improvement of NADPH bioavailability in *Escherichia coli* through the use of phosphofructokinase deficient strains. Appl. Microbiol. Biotechnol. 97, 6883–6893. 10.1007/s00253-013-4859-0 23558585

[B359] Watanabe S. Sasaki D. Tominaga T. Miki K. (2012). Structural basis of [NiFe] hydrogenase maturation by Hyp proteins. Biol. Chem. 393, 1089–1100. 10.1515/hsz-2012-0197 23096350

[B360] Weckbecker A. Hummel W. (2004). Improved synthesis of chiral alcohols with *Escherichia coli* cells co-expressing pyridine nucleotide transhydrogenase, NADP+-dependent alcohol dehydrogenase and NAD+-dependent formate dehydrogenase. Biotechnol. Lett. 26, 1739–1744. 10.1007/s10529-004-3746-2 15604828

[B361] Wells M. A. Mercer J. Mott R. A. Pereira-Medrano A. G. Burja A. M. Radianingtyas H.. (2011). Engineering a non-native hydrogen production pathway into *Escherichia coli* via a cyanobacterial [NiFe] hydrogenase. Metab. Eng. 13, 445–453. 10.1016/j.ymben.2011.01.004 21276867

[B362] Whitehead S. J. Iwaki M. Cotton N. P. J. Rich P. R. Jackson J. B. (2009). Inhibition of proton-transfer steps in transhydrogenase by transition metal ions. Biochim. Biophys. Acta 1787, 1276–1288. 10.1016/j.bbabio.2009.06.001 19505432

[B363] Whitehead S. J. Rossington K. E. Hafiz A. Cotton N. P. J. Jackson J. B. (2005). Zinc ions selectively inhibit steps associated with binding and release of NADP(H) during turnover of proton-translocating transhydrogenase. FEBS Lett. 579, 2863–2867. 10.1016/j.febslet.2005.04.026 15878164

[B364] Wichmann R. Vasic-Racki D. (2005). Cofactor regeneration at the lab scale. Adv. Biochem. Eng. Biotechnol. 92, 225–260. 10.1007/b98911 15791939

[B365] Xu Z. Jing K. Liu Y. Cen P. (2007). High-level expression of recombinant glucose dehydrogenase and its application in NADPH regeneration. J. Ind. Microbiol. Biotechnol. 34, 83–90. 10.1007/s10295-006-0168-2 16941118

[B366] Yamauchi Y. Hirasawa T. Nishii M. Furusawa C. Shimizu H. (2014). Enhanced acetic acid and succinic acid production under microaerobic conditions by *Corynebacterium glutamicum* harboring *Escherichia coli* transhydrogenase gene pntAB. J. Gen. Appl. Microbiol. 60, 112–118. 10.2323/jgam.60.112 25008167

[B367] Yamazaki M. Ichikawa Y. (1990). Some properties of the apoenzyme of NADPH-adreno-ferredoxin reductase from bovine adrenocortical mitochondria. Int. J. Biochem. 22, 1147–1152. 10.1016/0020-711X(90)90113-H 2289620

[B368] Yan Z. Nam Y.-W. Fushinobu S. Wakagi T. (2014). *Sulfolobus tokodaii* ST2133 is characterized as a thioredoxin reductase-like ferredoxin:NADP+ oxidoreductase. Extremophiles 18, 99–110. 10.1007/s00792-013-0601-1 24292509

[B369] Yang Z. Zhang H. Hung H.-C. Kuo C.-C. Tsai L.-C. Yuan H. S.. (2002). Structural studies of the pigeon cytosolic NADP(+)-dependent malic enzyme. Protein Sci. 11, 332–341. 10.1110/ps.38002 11790843 PMC2373443

[B370] Yeom J. Jeon C. O. Madsen E. L. Park W. (2009). *In vitro* and *in vivo* interactions of ferredoxin-NADP+ reductases in *Pseudomonas putida*. J. Biochem. 145, 481–491. 10.1093/jb/mvn185 19122206

[B371] Ying W. (2008). NAD+/NADH and NADP+/NADPH in cellular functions and cell death: regulation and biological consequences. Antioxid. Redox Signal. 10, 179–206. 10.1089/ars.2007.1672 18020963

[B372] Yokooji Y. Sato T. Fujiwara S. Imanaka T. Atomi H. (2013). Genetic examination of initial amino acid oxidation and glutamate catabolism in the hyperthermophilic archaeon *Thermococcus kodakarensis*. J. Bacteriol. 195, 1940–1948. 10.1128/JB.01979-12 23435976 PMC3624576

[B373] Yoon J.-J. Hattori T. Shimada M. (2003). Purification and characterization of NADP-linked isocitrate dehydrogenase from the copper-tolerant wood-rotting basidiomycete *Fomitopsis palustris*. Biosci. Biotechnol. Biochem. 67, 114–120. 10.1271/bbb.67.114 12619682

[B374] Yun H. Choi H.-L. Fadnavis N. W. Kim B.-G. (2005). Stereospecific synthesis of (R)-2-hydroxy carboxylic acids using recombinant *E. coli* BL21 overexpressing YiaE from *Escherichia coli* K12 and glucose dehydrogenase from *Bacillus subtilis*. Biotechnol. Prog. 21, 366–371. 10.1021/bp049694w 15801772

[B375] Zalacain M. Biswas S. Ingraham K. A. Ambrad J. Bryant A. Chalker A. F.. (2003). A global approach to identify novel broad-spectrum antibacterial targets among proteins of unknown function. J. Mol. Microbiol. Biotechnol. 6, 109–126. 10.1159/000076741 15044829

[B376] Zamboni N. Fischer E. Laudert D. Aymerich S. Hohmann H.-P. Sauer U. (2004). The *Bacillus subtilis* yqjI gene encodes the NADP+-dependent 6-P-gluconate dehydrogenase in the pentose phosphate pathway. J. Bacteriol. 186, 4528–4534. 10.1128/JB.186.14.4528-4534.2004 15231785 PMC438568

[B377] Zhang J.-D. Li A.-T. Yu H.-L. Imanaka T. Xu J.-H. (2011). Synthesis of optically pure S-sulfoxide by *Escherichia coli* transformant cells coexpressing the P450 monooxygenase and glucose dehydrogenase genes. J. Ind. Microbiol. Biotechnol. 38, 633–641. 10.1007/s10295-010-0809-3 20721599

[B378] Zhang J. Xu J. H. (2010). Biocatalysis, cofactor regeneration. Encyclopedia Ind. Biotechnol. 1–9. 10.1002/9780470054581.eib073

[B379] Zhao H. Van Der Donk W. A. (2003). Regeneration of cofactors for use in biocatalysis. Curr. Opin. Biotechnol. 14, 583–589. 10.1016/j.copbio.2003.09.007 14662386

[B380] Zhao H. Wang P. Huang E. Ge Y. Zhu G. (2008). Physiologic roles of soluble pyridine nucleotide transhydrogenase in *Escherichia coli* as determined by homologous recombination. Ann. Microbiol. 58, 275–280. 10.1007/BF03175329

[B381] Zhao J. Baba T. Mori H. Shimizu K. (2004a). Effect of zwf gene knockout on the metabolism of *Escherichia coli* grown on glucose or acetate. Metab. Eng. 6, 164–174. 10.1016/j.ymben.2004.02.004 15113569

[B382] Zhao J. Baba T. Mori H. Shimizu K. (2004b). Global metabolic response of *Escherichia coli* to gnd or zwf gene-knockout, based on 13C-labeling experiments and the measurement of enzyme activities. Appl. Microbiol. Biotechnol. 64, 91–98. 10.1007/s00253-003-1458-5 14661115

[B383] Zhao Z. Kuijvenhoven K. Van Gulik W. M. Heijnen J. J. Van Winden W. A. Verheijen P. J. T. (2011). Cytosolic NADPH balancing in *Penicillium chrysogenum* cultivated on mixtures of glucose and ethanol. Appl. Microbiol. Biotechnol. 89, 63–72. 10.1007/s00253-010-2851-5 20809073 PMC3016204

[B384] Zheng H. Ohno Y. Nakamori T. Suye S.-I. (2009). Production of L-malic acid with fixation of HCO3(-) by malic enzyme-catalyzed reaction based on regeneration of coenzyme on electrode modified by layer-by-layer self-assembly method. J. Biosci. Bioeng. 107, 16–20. 10.1016/j.jbiosc.2008.09.009 19147103

[B385] Zhou J. Olson D. G. Argyros D. A. Deng Y. Van Gulik W. M. Van Dijken J. P.. (2013). Atypical glycolysis in *Clostridium thermocellum*. Appl. Environ. Microbiol. 79, 3000–3008. 10.1128/AEM.04037-12 23435896 PMC3623140

[B386] Zhu G. Golding G. B. Dean A. M. (2005). The selective cause of an ancient adaptation. Science 307, 1279–1282. 10.1126/science.1106974 15653464

[B387] Ziegler M. (2000). New functions of a long-known molecule. Eur. J. Biochem. 267, 1550–1564. 10.1046/j.1432-1327.2000.01187.x 10712584

